# Protective Efficacy and Safety of Three Antimalarial Regimens for the Prevention of Malaria in Young Ugandan Children: A Randomized Controlled Trial

**DOI:** 10.1371/journal.pmed.1001689

**Published:** 2014-08-05

**Authors:** Victor Bigira, James Kapisi, Tamara D. Clark, Stephen Kinara, Florence Mwangwa, Mary K. Muhindo, Beth Osterbauer, Francesca T. Aweeka, Liusheng Huang, Jane Achan, Diane V. Havlir, Philip J. Rosenthal, Moses R. Kamya, Grant Dorsey

**Affiliations:** 1 Infectious Diseases Research Collaboration, Kampala, Uganda; 2 Department of Medicine, San Francisco General Hospital, University of California, San Francisco, California, United States of America; 3 Department of Pediatrics, Makerere University College of Health Sciences, Kampala, Uganda; 4 Department of Medicine, Makerere University College of Health Sciences, Kampala, Uganda; University of Melbourne, Australia

## Abstract

**Background:**

Chemoprevention offers a promising strategy for prevention of malaria in African children. However, the optimal chemoprevention drug and dosing strategy is unclear in areas of year-round transmission and resistance to many antimalarial drugs. To compare three available regimens, we conducted an open-label randomized controlled trial of chemoprevention in Ugandan children.

**Methods and Findings:**

This study was conducted between June 28, 2010, and September 25, 2013. 400 infants were enrolled and 393 randomized at 6 mo of age to no chemoprevention, monthly sulfadoxine-pyrimethamine (SP), daily trimethoprim-sulfamethoxazole (TS), or monthly dihydroartemisinin-piperaquine (DP). Study drugs were administered at home without supervision. Piperaquine (PQ) levels were used as a measure of compliance in the DP arm. Participants were given insecticide-treated bednets, and caregivers were encouraged to bring their child to a study clinic whenever they were ill. Chemoprevention was stopped at 24 mo of age, and participants followed-up an additional year. Primary outcome was the incidence of malaria during the intervention period. During the intervention, the incidence of malaria in the no chemoprevention arm was 6.95 episodes per person-year at risk. Protective efficacy was 58% (95% CI, 45%–67%, *p*<0.001) for DP, 28% (95% CI, 7%–44%, *p* = 0.01) for TS, and 7% for SP (95% CI, −19% to 28%, *p* = 0.57). PQ levels were below the detection limit 52% of the time when malaria was diagnosed in the DP arm, suggesting non-adherence. There were no differences between the study arms in the incidence of serious adverse events during the intervention and the incidence of malaria during the 1-y period after the intervention was stopped.

**Conclusions:**

For preventing malaria in children living in an area of high transmission intensity, monthly DP was the most efficacious and safe, although adherence may pose a problem. Monthly SP and daily TS may not be appropriate in areas with high transmission intensity and frequent resistance to antifolates.

**Trial registration:**

www.ClinicalTrials.gov NCT00948896

*Please see later in the article for the Editors' Summary*

## Abstract 2

Grant Dorsey and colleagues investigate the efficacy of three antimalarial drugs for preventing malaria in children living in Uganda, an area of high transmission intensity.

*Please see later in the article for the Editors' Summary*

## Editors' Summary

**Background:**

Malaria is a parasitic disease that kills more than 600,000 people (mainly young children living in sub-Saharan Africa) every year. Malaria parasites, which are transmitted to people through the bites of night-flying mosquitoes, cause a characteristic fever that needs to be treated promptly with antimalarial drugs to prevent anemia and organ damage. Prompt treatment also helps to reduce malaria transmission and is a component of the Global Malaria Action Plan, which aims to control and eventually eliminate malaria. Other components of this plan include the provision of insecticide-treated bednets for people to sleep under to avoid mosquito bites and indoor residual spraying with insecticides. Widespread deployment of these preventative tools and the increased availability of effective antimalarial drugs have greatly reduced malaria-related deaths worldwide over the past decade, but new strategies are still urgently needed to reduce the burden of malaria among those most at risk—young children living in Africa.

**Why Was This Study Done?:**

One promising strategy for the prevention of malaria in African children is the use of antimalarial drugs to prevent rather than treat malaria. In trials, giving infants sulfadoxine-pyrimethamine (SP) alongside routine vaccinations, for example, reduced the incidence of malaria (the number of new cases in the population in a year) by about 30% during the first year of life (a protective efficacy of 30%). However, the optimal chemoprevention drug and dosing strategy for children living in African regions where there is year-round transmission of malaria and where resistance to antimalarial drugs is common remains unclear. Here, the researchers undertake an open-label randomized controlled trial (RCT) of chemoprevention in infants in the Tororo District of eastern Uganda, an area with intense year-round malaria transmission. RCTs compare outcomes in groups of people chosen to receive different interventions through the play of chance; in open-label RCTs, both the researchers and the participants know which treatment is being administered.

**What Did the Researchers Do and Find?:**

The researchers assigned 393 six-month-old infants to receive no chemoprevention, monthly SP, daily trimethoprim-sulfamethoxazole (TS), or monthly dihydroartemisinin-piperaquine (DP) until they were 24 months old. SP and TS block the production of folic acid, which malaria needs for survival, whereas DP is a newer artemisinin-based combination therapy (ACT). All the drugs were given at home without supervision, and caregivers were asked to bring their children to a study clinic whenever they were ill. During the intervention, the incidence of malaria was 6.95 episodes per person-year at risk in the no chemoprevention arm but only three episodes per person-year at risk in the DP arm. That is, the protective efficacy of DP was 58%. By contrast, the protective efficacies of TS and SP were 28% and 7%, respectively. However, for SP the protective efficacy was not statistically different compared to the no chemoprevention arm. Notably, piperaquine levels on the day that malaria was diagnosed were below the detection limit in half of the malaria episodes in the DP arm, which suggests that a complete dose of DP had not been given to the infant in the previous month, despite caregivers reporting that they had administered virtually all the assigned doses. Finally, the incidence of serious adverse events was similar in all the study arms during the intervention, as was the incidence of malaria during the year after the intervention, which suggests that the chemoprevention strategies did not affect the development of naturally acquired immunity.

**What Do These Findings Mean?:**

These findings show that, for children living in an area of intense malaria transmission, monthly DP was the most efficacious strategy for malaria chemoprevention but that adherence to the strategy may have been a problem. These findings also suggest that monthly SP and daily TS may not be appropriate chemoprevention strategies in areas of high transmission intensity, particularly those where resistance to antifolate drugs is common. The accuracy of these findings may be affected by drug administration being self-reported and by the number of comparisons included in the trial, which may have increased the risk of false-positive results. Moreover, the results of this trial may not be generalizable to other regions of sub-Saharan Africa. Overall, however, these results suggest that monthly DP is a strategy worth considering in regions in need of improved malaria control measures, with the important caveat that widespread ACT use for chemoprevention could compromise the efficacy of ACT when used for treatment.

**Additional Information:**

Please access these websites via the online version of this summary at http://dx.doi.org/10.1371/journal.pmed.1001689.

- Information is available from the World Health Organization on malaria (in several languages), including information on malaria chemoprevention; the World Malaria Report 2013 provides details of the current global malaria situation, including information on malaria in Uganda
- The US Centers for Disease Control and Prevention provide information on malaria, including information on ways to reduce malaria cases and deaths; it also provides a selection of personal stories about malaria, including a story about malaria in a child in Africa
- Information is available from the Roll Back Malaria Partnership on the global control of malaria and on the Global Malaria Action Plan (in English and French); its website includes fact sheets about malaria in Africa and about children and malaria
- MedlinePlus provides links to additional information on malaria (in English and Spanish)
- More information about this trial is available

## Introduction

Malaria remains one of the most important infectious diseases worldwide, with an estimated 219 million cases and 660,000 deaths in 2010 [1]. Approximately 90% of deaths occurred in Africa, with children under 5 y of age the most severely affected [1]. Malaria control for African children largely relies on the use of insecticide-treated bednets (ITNs), indoor residual spraying of insecticide, and case management with artemisinin-based combination therapy (ACT). However, there are limitations to the effectiveness of all of these interventions, and new strategies are urgently needed to reduce the burden of malaria for those at greatest risk.

The use of antimalarial drugs for the prevention of malaria in children at high risk has recently received widespread attention. Most studied is intermittent preventive therapy in infants (IPTi) with sulfadoxine-pyrimethamine (SP) at the time of routine vaccinations. A pooled analysis of six randomized controlled trials reported that IPTi with SP was safe and was associated with a 30% (95% CI, 19%–39%) protective efficacy against malaria in the first year of life [2]. Daily chemoprophylaxis with another antifolate combination, trimethoprim-sulfamethoxazole (TS), has also been shown to be effective for the prevention of malaria in HIV-infected and -uninfected children, with protective efficacies of 39% (95% CI, 19%–54%) and 99% (95% CI, 96%–100%) in randomized trials from Uganda and Mali, respectively [3],[4]. In parts of west Africa where transmission is largely restricted to a few months each year and antifolate resistance is uncommon, seasonal malaria chemoprevention (SMC) at monthly intervals during the transmission season has been highly effective. In a systematic review and meta-analysis of 12 studies, SMC with regimens including SP primarily combined with amodiaquine (AQ) provided an overall protective efficacy of 82% (95% CI, 75%–87%) against malaria and 57% (95% CI, 24%–76%) against all-cause mortality [5]. However, alternative drugs and/or approaches may be needed in areas where the prevalence of resistance to antifolate drugs is high or malaria transmission occurs throughout the year.

To compare available drug regimens for the prevention of malaria in an area with high antifolate resistance and intense, year-round transmission, we conducted a randomized controlled trial evaluating three regimens for the prevention of malaria in young children in Tororo, Uganda: monthly SP, daily TS, and monthly dihydroartemisinin-piperaquine (DP), an ACT that is especially attractive because of its excellent treatment efficacy and prolonged post-treatment prophylactic effect [6]. All study participants were given an ITN at enrollment, and the primary objective was to compare the incidence of malaria between the three chemoprevention arms and a control group that received no chemoprevention.

## Methods

### Ethics Statement

Ethical approval was obtained from the Uganda National Council for Science and Technology, the Makerere University School of Medicine Research and Ethics Committee, and the University of California, San Francisco, Committee on Human Research.

### Study Design, Site, and Population

We performed a randomized, controlled, open-label trial from June 28, 2010, to September 25, 2013, comparing the efficacy and safety of three regimens versus no therapy for the prevention of malaria in Tororo District, eastern Uganda, an area with intense year-round malaria transmission and an entomological inoculation rate estimated at 562 infectious bites per person-year in 2002 [7].

Convenience sampling was used to enroll a cohort of 400 infants of 4–5 mo of age from the Tororo District Hospital Maternal and Child Health Clinic between June 28, 2010, and February 24, 2011. Eligibility criteria included the following: (1) born to HIV-uninfected mothers, (2) residency within 30 km of the study clinic with no intention of moving outside the study area, (3) agreement to come to the study clinic for any illness and to avoid medications outside the study protocol, (4) provision of informed consent by parent/guardian, (5) no history of allergy or sensitivity to any study drugs, (6) absence of active medical problem requiring in-patient evaluation or chronic medical conditions requiring frequent attention, and (7) absence of clinically significant electrocardiogram (ECG) abnormalities, family history of long QT syndrome, and current use of drugs that prolong the QTc interval. Only one eligible child was enrolled per household, and at enrollment each household was given two long-lasting ITNs. The purposes and benefits of sleeping under the ITNs were explained to the primary caregivers, and ITN use was encouraged throughout the study.

### Study Drugs and Treatment Allocation

A randomization list using permuted variable-sized blocks of 4, 8, and 12 was computer generated by a member of the study not directly involved in patient care. Study participants were randomized to their assigned treatment group at 6 mo of age using premade, consecutively numbered, sealed envelopes. Treatment allocation was performed by nurses not involved with patient care. Study drugs were dosed as follows: TS (co-trimoxazole, Kampala Pharmaceutical Industries, Uganda), single dose once daily; SP (Kamsidar, Kampala Pharmaceutical Industries, Uganda), single dose each month; and DP (Duo-Cotexin, Beijing Holley-Cotec Pharmaceuticals, China), once daily for three consecutive days each month; each drug was provided for administration at home according to weight-based guidelines. All study drugs were in compliance with local Good Manufacturing Practices. At the time of treatment allocation, parents/guardians were given a 2-mo supply of drugs and a diary with dates for dosing and check-offs to indicate administration. Parents/guardians were instructed to re-administer drugs if children vomited within 30 min of administration and to bring children to the clinic if they vomited again. During each visit to the study clinic, parent/guardians were questioned about study drug use and resupplied to maintain a 2-mo supply.

### Study Procedures

Participants received all of their medical care at a designated study clinic open every day. Parents/guardians were encouraged to bring their children to the clinic any time they were ill. Children who presented with a documented fever (tympanic temperature ≥38.0°C) or history of fever in the previous 24 h had blood obtained by finger prick for a thick blood smear. If the smear was positive, the patient was diagnosed with malaria, and a complete blood count and thin blood smear for parasite speciation were performed. Episodes of uncomplicated malaria were treated with artemether-lumefantrine (AL), the recommended first-line treatment in Uganda. AL was administered twice a day for 3 d, with the first daily dose given directly observed in the clinic and the second daily dose administered at home. Episodes of complicated malaria (severe malaria or danger signs) [8] or treatment failures occurring within 14 d of prior therapy were treated with quinine. Routine evaluations, including thick blood smears and assessment of use of ITNs and adherence to study drugs, were done monthly. Complete blood count and glucose and alanine aminotransferase levels were assessed every 4 mo. ECGs were performed monthly in every fifth study participant randomized to DP if they could be brought to the clinic on the day they took their third dose in a given month. Adverse events were assessed and graded according to severity (mild, moderate, severe, life-threatening) using standardized criteria at every clinic visit. A serious adverse event was defined as any adverse experience that resulted in death, life-threatening experience, participant hospitalization, persistent or significant disability or incapacity, or specific medical or surgical intervention to prevent serious outcome. Diagnosis of incident episodes of non-malarial illnesses, including diarrheal illnesses and respiratory tract infections, were based on a prespecified list of diagnostic criteria developed by the study team. Medications with antimalarial activity were avoided for the treatment of non-malarial illnesses when possible. Antihelmintics, iron sulfate, and vitamin A were prescribed following Integrated Management of Childhood Illnesses guidelines.

Chemoprevention was stopped at 24 mo of age, and study participants were followed-up one additional year until they reached 36 mo of age. Study participants were prematurely withdrawn from the study for (1) movement out of the study area, (2) failure to be seen in the study clinic for >60 consecutive days, (3) withdrawal of informed consent, or (4) inability to comply with the study schedule and procedures.

### Laboratory Procedures

Thick and thin blood smears were stained with 2% Giemsa for 30 min. Parasite density was estimated by counting the number of asexual parasites per 200 white blood cells and assuming a white blood cell count of 8,000 per microliter. A smear was deemed negative if no parasites were seen in 100 high-powered fields. Microscopy quality control included rereading all blood smears and resolution of any discrepancies by a third microscopist.

Piperaquine (PQ) drug levels were measured from capillary blood collected on filter paper on the day malaria was diagnosed among study participants randomized to monthly DP. Levels of drugs from the other treatment arms were not measured because of resource constraints. Briefly, dried blood spots were punched from Whatman cards and extracted with 100 µl of a 1∶1 mixture of 20% trichloroacetic acid and acetonitrile. Nevirapine-d_5_ was used as the internal standard because its retention time (1.17 min) was the closest to that of PQ (1.21 min) among several compounds screened. The extract (10 µl) was directly injected onto a liquid chromatography–tandem mass spectrometry system API5000. Separation was achieved on a PFP column (2.1×50 mm, 3 µm) eluted with aqueous 0.14% trifluoroacetic acid, 10 mM ammonium formate, and acetonitrile. The PQ assay demonstrated a lower limit of quantitation of 10 ng/ml with a calibration range of 10–100 ng/ml. Inter- and intra-day accuracy ranged from 97.6% to 106% and from 93.9% to 112%, respectively, and inter- and intra-day variation ranged from 7.4% to 12% and from 4.4% to 14%, respectively.

### Statistical Analysis

This study was part of a protocol that included two randomized controlled trials to evaluate the protective efficacy and safety of antimalarial chemopreventive regimens in distinct populations of HIV-unexposed (HIV-uninfected children born to HIV-uninfected mothers) and HIV-exposed (HIV-uninfected children born to HIV-infected mothers) children. Here we only present the findings from the trial conducted in HIV-unexposed children. The other results will be presented elsewhere. The study presented here was designed to test the hypotheses that chemoprevention lowers the incidence of malaria compared to no chemoprevention, and that the optimal chemoprevention regimen is DP. The sample size was calculated to detect at least a 32% lower incidence of malaria in the DP arm compared to that in the TS arm. We assumed that the incidence of malaria would be 1.85 episodes per person-year with TS chemoprevention based on a prior cohort study in the same area [3], and thus we calculated that we would need to enroll 100 participants in each arm to detect our targeted protective efficacy with 80% power at 95% significance (two-sided), allowing for 10% loss to follow-up.

Data were double-entered and verified in Microsoft Access, and statistical analyses were performed using Stata, version 12 (StataCorp). All analyses used a modified intention-to-treat approach, including all study participants randomized to therapy and including all follow-up time until the study participant reached a study end point or early study termination, regardless of whether the intervention was stopped due to an adverse event. Descriptive statistics included means and standard deviations for continuous variables and proportions for categorical variables. The primary outcome was the incidence of malaria, defined as the number of incident episodes per time at risk, during the period the intervention was given (6–24 mo of age). Treatments within 14 d of a prior episode were not considered incident events. Time at risk was from the day following the initiation of study drugs to the last day of observation, minus 14 d after each treatment for malaria. Secondary outcomes included the incidence of complicated malaria, all-cause hospitalizations, diarrheal illnesses, respiratory tract infections, and serious adverse events or adverse events of moderate or greater severity (grade 3–4); the prevalence of moderate–severe anemia (hemoglobin<8 gm/dl) measured at the time of each episode of malaria and at the time of routine testing done every 4 mo; and the prevalence of parasitemia and gametocytemia measured at the time of monthly routine blood smears in study participants who were asymptomatic. Response to antimalarial therapy among children diagnosed with symptomatic malaria was also a secondary outcome, and will be presented separately. To assess the impact of chemoprevention on the development of naturally acquired immunity, the incidence of malaria, complicated malaria, and hospitalizations was compared between treatment arms for children 24–36 mo of age after the intervention was stopped.

Incidence outcomes were compared using a negative binomial regression model, and prevalence outcomes and measure of use of ITNs at the time of monthly assessments were compared using generalized estimating equations with adjustment for repeated measures in the same study participant. For all analyses, only the assigned study arm was included as a covariate, with the exception of a multivariable analysis performed for the primary outcome of malaria incidence, which also included other covariates that were thought to be potential independent risk factors for malaria. Measures of association were expressed as protective efficacy (PE = 1 minus the incident rate ratio or prevalence ratio) during the intervention and incident rate ratios after the intervention was stopped. *p*<0.05 was considered statistically significant.

## Results

**Figure 1:**
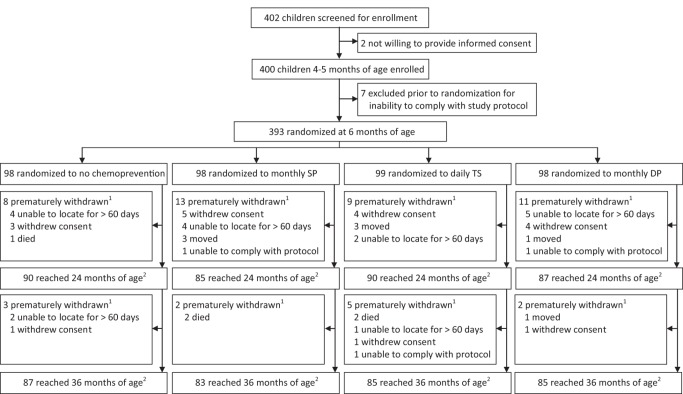
Trial profile. ^1^Only data that accrued until the study participant was prematurely withdrawn from the study were included in the modified intention-to-treat analysis. ^2^Complete data included in the modified intention-to-treat analysis.

400 infants were enrolled, 393 (98%) of whom reached 6 mo of age and were randomized to one of the four study arms (Figure 1). Baseline characteristics at enrollment were similar across all study arms (Table 1). At enrollment 24% of study participants had a positive thick blood smear (all asymptomatic parasitemia). Among the 393 infants randomized to therapy, 352 (89.6%) were followed up to 24 mo of age, and 340 (86.5%) were followed up to 36 mo of age, representing 93.6% and 91.4% of potential follow-up time, respectively (Figure 1). All families were given two ITNs at enrollment; 98.5% of study participants reported sleeping under an ITN at monthly assessments, without significant differences between study arms (*p* = 0.36).

**Table 1:** Baseline characteristics at enrollment of study participants randomized to the intervention.

Characteristic	Intervention Arm			
	Control (*n* = 98)	Monthly SP (*n* = 98)	Daily TS (*n* = 99)	Monthly DP (*n* = 98)
Age in months, mean (SD)	5.4 (0.5)	5.3 (0.5)	5.4 (0.5)	5.4 (0.5)
Female gender, *n* (percent)	48 (49.0%)	44 (44.9%)	48 (48.5%)	52 (53.1%)
Slept under any bednet prior night, *n* (percent)	41 (41.8%)	43 (43.9%)	40 (40.4%)	45 (45.9%)
Slept under ITN prior night, *n* (percent)	27 (27.6%)	30 (30.6%)	27 (27.3%)	29 (29.6%)
Stunted^a^, *n* (percent)	10 (10.2%)	11 (11.2%)	6 (6.1%)	4 (4.1%)
Wasted^b^, *n* (percent)	4 (4.1%)	7 (7.1%)	7 (7.1%)	5 (5.1%)
Hemoglobin (gm/dl), mean (SD)	9.7 (1.4)	9.6 (1.8)	9.9 (1.5)	9.5 (1.5)
Positive thick blood smear, *n* (percent)	22 (22.5%)	27 (27.6%)	17 (17.2%)	28 (28.6%)

a Length-for-age *z*-score<−2.

b Weight-for-age *z*-score<−2.

SD, standard deviation.

**Figure 2:**
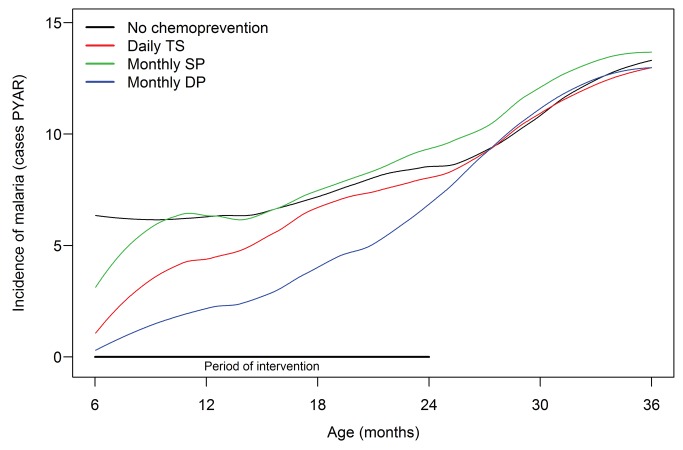
Incidence of malaria over age, stratified by assigned study arm. PYAR, person-years at risk.

During the intervention, the incidence of malaria in the no chemoprevention group was 6.95 episodes per person-year at risk (Table 2). Compared to the no chemoprevention group, monthly SP provided no significant protection against malaria (PE = 7%, 95% CI, −19% to 28%, *p* = 0.57), daily TS was moderately protective (PE = 28%, 95% CI, 7%–44%, *p* = 0.01), and monthly DP provided the greatest protection (PE = 58%, 95% CI, 45%–67%, *p*<0.001). Results were very similar when using multivariable analyses to adjust for household wealth index, level of education of primary caregiver, whether the child was parasitemic at enrollment, residence in village or town, nutritional status at enrollment (defined as length-for-age *z*-score), and type of materials used for house construction (Table 3). In all four arms the incidence of malaria increased with age (Figure 2), and the protective efficacies of all three interventions were greater for children at age 6–11 mo than at age 12–24 mo (Table 2). When compared to daily TS, the protective efficacy of monthly DP was 41% (95% CI, 21%–56%, *p*<0.001).

**Table 2:** Protective efficacy against incident episodes of malaria overall and stratified by age.

Study Arm	6–24 mo of Age					6–11 mo of Age			12–24 mo of Age		
	Number of Cases	PYAR	Incidence per PYAR	PE (95% CI)	*p*-Value	Incidence per PYAR	PE (95% CI)	*p*-Value	Incidence per PYAR	PE (95% CI)	*p*-Value
Control	760	109.3	6.95	Reference	—	6.41	Reference	—	7.24	Reference	—
Monthly SP	725	107.8	6.73	7% (−19% to 28%)	0.57	5.51	17% (−12% to 39%)	0.21	7.41	−3% (−34% to 20%)	0.81
Daily TS	609	116.8	5.21	28% (7% to 44%)	0.01	3.27	51% (33% to 64%)	<0.001	6.32	11% (−15% to 31%)	0.38
Monthly DP	366	121.3	3.02	58% (45% to 67%)	<0.001	1.49	78% (69% to 85%)	<0.001	3.88	45% (28% to 58%)	<0.001

PYAR, person-years at risk.

**Table 3:** Protective efficacy against incident episodes of malaria using multivariable analyses.

Study Arm	Number of Cases	PYAR	Incidence per PYAR	Adjusted PE (95% CI)^a^	*p*-Value
Control	760	109.3	6.95	Reference	—
Monthly SP	725	107.8	6.73	8% (−15% to 27%)	0.45
Daily TS	609	116.8	5.21	26% (6% to 41%)	0.01
Monthly DP	366	121.3	3.02	60% (49% to 69%)	<0.001

a Adjusted for household wealth index, level of education of primary caregiver, whether the child was parasitemic at enrollment, residence in village or town, type of materials used for house construction, and nutritional status at enrollment (defined as length-for-age *z*-score).

PYAR, person-years at risk.

**Figure 3:**
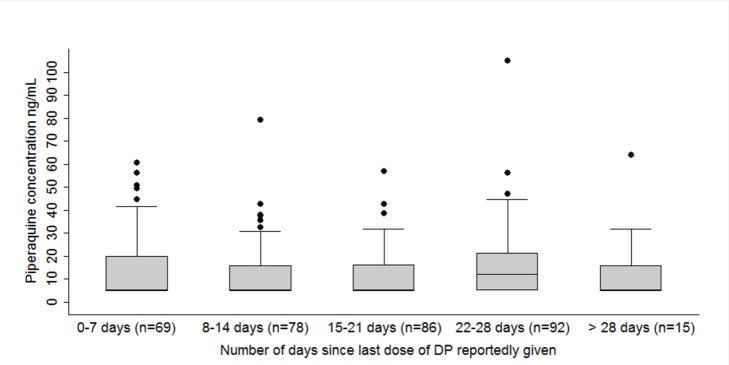
Vertical box plots of piperaquine concentrations at the time of each episode of malaria, stratified by number of days since last dose of DP reportedly given. 10 ng/ml represents the lower limit of detection.

The proportions of assigned doses reportedly administered at home based on diaries completed by primary caregivers were 99.2% for SP, 98.5% for TS, and 99.2% for DP. To further explore adherence among study participants randomized to monthly DP, PQ blood concentrations were measured at the time of each episode of malaria. Among 366 episodes, 348 samples were available for analysis. For ten episodes, PQ levels were >100 ng/ml, and the primary caregivers reported giving the DP within the prior 24 h, suggesting the coincidental onset of malaria just before the scheduled time for monthly DP dosing. For the remaining 338 samples there was no relationship between PQ levels and the number of days since the last dose of DP was reported given (Figure 3). In addition, PQ levels were below the level of detection (<10 ng/ml) on the day malaria was diagnosed in 52% of episodes, suggesting that a complete dose of DP was not administered in the previous month [9],[10]. Thus, despite caregiver reports to the contrary, our results suggest frequent non-adherence with study dosing schedules.

During the intervention, a total of 2,487 treatments were given for malaria (96.7% *Plasmodium falciparum*), including 2,460 for incident episodes and 27 for episodes occurring within 14 d of a prior treatment. A total of 21 (0.8%) treatments were for complicated malaria, 12 for danger signs (seven single convulsions, three lethargy, and two vomiting), and nine for severe malaria (five severe anemia, three multiple convulsions, and one cerebral malaria). A total of 58 hospitalizations occurred in 34 different children, most commonly for anemia without complicated malaria (16), complicated malaria (14), gastrointestinal illness (seven), respiratory tract infection (six), measles (four), and septicemia (four). There were no significant differences in the incidence of complicated malaria, hospitalization, diarrheal illnesses, and respiratory tract infections between any of the intervention arms and the no chemoprevention arm (Table 4). There was a single death, in an 8-mo-old child randomized to no chemoprevention, which occurred 2 d after the child was admitted for pneumonia. Compared to the no chemoprevention arm, the prevalence of moderate–severe anemia (hemoglobin<8 gm/dl) was significantly higher in the SP arm (PE = −70%, 95% CI, −184% to −2%, *p* = 0.04) and significantly lower in the DP arm (PE = 47%, 95% CI, 1% to 72%, *p* = 0.04) (Table 4). The mean change in hemoglobin level from enrollment to the end of the intervention period was higher in the TS and DP arms compared to the control arm, but none of these comparisons reached statistical significance: control, 0.84 gm/dl; SP, 0.82 gm/dl; TS, 0.97 gm/dl; and DP, 1.21 gm/dl. The prevalence of asymptomatic parasitemia and gametocytemia at the time of routine blood smears was also significantly lower in the DP arm compared to the no chemoprevention arm (PE = 76%, 95% CI, 59% to 86%, *p*<0.001, and PE = 89%, 95% CI, 48% to 98%, *p* = 0.006, respectively) (Table 4).

**Table 4:** Comparative effectiveness against secondary outcomes.

Outcome	Study Arm	Number of Cases or Prevalence (Percent)	PYAR	Incidence per PYAR	PE (95% CI)	*p*-Value
**Complicated malaria**	Control	4	138.3	0.029	Reference	—
	Monthly SP	6	135.5	0.044	−53% (−583% to 66%)	0.58
	Daily TS	6	140.1	0.043	−49% (−561% to 67%)	0.60
	Monthly DP	5	135.2	0.037	−29% (−501% to 72%)	0.74
**All-cause hospital admissions**	Control	8	138.5	0.058	Reference	—
	Monthly SP	25	135.8	0.184	−187% (−824% to 11%)	0.08
	Daily TS	12	140.3	0.086	−36% (−369% to 61%)	0.63
	Monthly DP	13	135.4	0.096	−58% (−441% to 54%)	0.47
**Diarrheal illnesses**	Control	241	138.5	1.74	Reference	—
	Monthly SP	245	135.8	1.80	−6% (−37% to 18%)	0.65
	Daily TS	271	140.3	1.93	−11% (−43% to 14%)	0.42
	Monthly DP	275	135.4	2.03	−16% (−50% to 10%)	0.24
**Respiratory tract infections**	Control	476	138.5	3.44	Reference	—
	Monthly SP	419	135.8	3.09	9% (−11% to 26%)	0.35
	Daily TS	461	140.3	3.29	4% (−18% to 22%)	0.72
	Monthly DP	505	135.4	3.73	−8% (−33% to 12%)	0.46
**Moderate–severe anemia (hemoglobin < 8 gm/dl)**	Control	66/1,112 (5.9%)			Reference	—
	Monthly SP	145/1,113 (13.0%)			−70% (−184% to −2%)	0.04
	Daily TS	85/1,049 (8.1%)			−21% (−107% to 29%)	0.49
	Monthly DP	25/899 (2.8%)			47% (1% to 72%)	0.04
**Asymptomatic parasitemia**	Control	60/528 (11.4%)			Reference	—
	Monthly SP	59/500 (11.8%)			−3% (−65% to 35%)	0.89
	Daily TS	85/670 (12.7%)			−10% (−70% to 29%)	0.68
	Monthly DP	24/849 (2.8%)			76% (59% to 86%)	<0.001
**Gametocytemia**	Control	11/528 (2.1%)			reference	—
	Monthly SP	18/500 (3.6%)			−70% (−349% to 36%)	0.29
	Daily TS	32/670 (4.8%)			−106% (−423% to 19%)	0.13
	Monthly DP	2/849 (0.2%)			89% (48% to 98%)	0.006

PYAR, person-years at risk.

Considering grade 3–4 adverse events regardless of relationship to study drugs, the overall incidence and the incidence of elevated temperature, anemia, and thrombocytopenia were significantly lower in the DP arm, but not the SP or TS arms, compared to the no chemoprevention arm (Table 5). Only 19 of 424 (4.5%) grade 3–4 adverse events were classified as possibly related to study drugs, with no significant differences between the three intervention arms. Study drugs were withheld for safety concerns on 13 occasions (six TS, four SP, and three DP) in 12 different participants; in each instance the adverse events resolved, and study drugs were restarted. There were no significant differences in the incidence of serious adverse events between the no chemoprevention and intervention arms; however, the incidence of serious adverse events was significantly lower in the DP arm compared to the SP arm (Table 5). ECGs were performed on 145 occasions in 19 different children on the day they received their third dose of DP within a given month; all QTc intervals were within normal limits.

**Table 5:** Comparative safety outcomes.

Outcome	Number of Events (Incidence per PYAR) by Study Arm			
	Control	Monthly SP	Daily TS	Monthly DP
**All grade 3–4 adverse events**	169 (1.159)	202 (1.415)	135 (0.914)	87 (0.611)***
**Individual grade 3–4 adverse events** a				
Elevated temperature	79 (0.542)	78 (0.546)	58 (0.393)	46 (0.323)**
Anemia	56 (0.384)	86 (0.602)	47 (0.318)	24 (0.168)**
Thrombocytopenia	18 (0.123)	17 (0.119)	9 (0.061)	5 (0.035)*
Elevated aspartate aminotransferase	7 (0.048)	8 (0.056)	6 (0.041)	3 (0.021)
Elevated alanine aminotransferase	4 (0.027)	4 (0.028)	4 (0.027)	3 (0.021)
Neutropenia	3 (0.021)	6 (0.042)	2 (0.014)	1 (0.007)
**Grade 3–4 adverse events possibly related to study drugs**	N/A	8 (0.056)	8 (0.054)	3 (0.021)
**All serious adverse events**	26 (0.178)	52 (0.364)	29 (0.196)	13 (0.091)

a Includes only those categories with at least five total events.

*p<0.05 compared to control group;

**p<0.01 compared to control group;

***p<0.001 compared to control group.

N/A, not applicable; PYAR, person-years at risk.

In the year after the intervention was stopped, the incidence of malaria in the no chemoprevention arm was 10.85 episodes per person-year at risk (Table 6) and continued to increase with age (Figure 2). There were no significant differences during the year after the intervention in the incidence of malaria between any of the groups that received chemoprevention and the no chemoprevention arm (Table 6). A total of 2,731 treatments were given for malaria in the year after the intervention, including 2,696 for incident episodes and 35 for episodes occurring within 14 d of a prior treatment. A total of 19 (0.7%) treatments were for complicated malaria, ten for danger signs (eight single convulsions, one lethargy, and one vomiting), and nine for severe malaria (all severe anemia). There were no significant differences in the incidence of complicated malaria between any of the groups that received prior chemopreventive therapy and the no chemoprevention arm (Table 6). A total of 52 hospitalizations occurred in 20 different children, most commonly for anemia without complicated malaria (29), complicated malaria (14), gastrointestinal illness (two), respiratory tract infection (two), and convulsions without malaria (two). The incidence of hospitalization was significantly higher in the group that had received monthly SP compared to the no chemoprevention arm (incidence rate ratio = 10.7, 95% CI, 2.17–53.2, *p* = 0.004) (Table 6). There were four deaths after chemoprevention was stopped (two in the monthly SP arm and two in the daily TS arm); three died of severe anemia without malaria and one died of severe malnutrition.

**Table 6:** Comparative outcomes after intervention was stopped (24–36 mo of age).

Outcome	Study Arm	Number of Cases	PYAR	Incidence per PYAR	IRR (95% CI)	*p*-Value
**All incident episodes of malaria**	No chemoprevention	670	61.8	10.85	1.00 (reference)	—
	Prior therapy with monthly SP	689	57.5	11.98	1.11 (0.88–1.40)	0.37
	Prior therapy with daily TS	678	62.2	10.90	1.01 (0.80–1.27)	0.95
	Prior therapy with monthly DP	659	61.2	10.77	0.99 (0.79–1.25)	0.96
**Complicated malaria**	No chemoprevention	4	87.4	0.046	1.00 (reference)	—
	Prior therapy with monthly SP	11	83.6	0.132	3.25 (0.38–27.4)	0.28
	Prior therapy with daily TS	4	87.8	0.046	0.99 (0.10–9.44)	0.99
	Prior therapy with monthly DP	0	86.6	0	Unable to estimate	—
**All-cause hospital admissions**	No chemoprevention	4	87.6	0.046	1.00 (reference)	—
	Prior therapy with monthly SP	38	84.0	0.452	10.7 (2.17–53.2)	0.004
	Prior therapy with daily TS	8	87.9	0.091	1.99 (0.36–11.1)	0.43
	Prior therapy with monthly DP	2	86.6	0.023	0.51 (0.06–4.14)	0.52

IRR, incidence rate ratio; PYAR, person-years at risk.

## Discussion

We evaluated the antimalarial protective efficacy of monthly SP, daily TS, and monthly DP in a high transmission region of Uganda where, despite use of ITNs, the incidence of malaria in those not receiving chemoprevention was almost seven episodes per person-year at risk for children 6–24 mo of age and almost 11 episodes per person-year at risk for children 24–36 mo of age. This finding underlines the fact that ITNs are not a panacea for the control of malaria in high transmission areas. Monthly SP had no protective efficacy against malaria and was associated with an increased prevalence of moderate–severe anemia. Daily TS reduced the incidence of malaria by 28% (95% CI, 1%–44%), but offered no protection against moderate–severe anemia. In contrast, monthly DP was highly effective, reducing the incidence of malaria by 58% (95% CI, 45%–67%), the prevalence of moderate–severe anemia by 47% (95% CI, 1%–72%), the prevalence of asymptomatic parasitemia by 76% (95% CI, 59%–86%), and the prevalence of gametocytemia by 89% (95% CI, 48%–98%). In terms of measures of impact, approximately 3 mo of chemopreventive therapy with monthly DP was needed for each episode of malaria prevented. There were no differences between the study arms in the incidence of malaria during the 1-y period after the intervention was stopped, suggesting that none of the chemoprevention regimens had a significant impact on the development of naturally acquired immunity.

The regular use of drugs for the prevention of malaria has been studied in African children for over 60 y. Earlier studies focused on continuous chemoprophylaxis, the frequent sub-therapeutic dosing of drugs to prevent infection or its clinical manifestations. In several randomized controlled trials chemoprophylaxis reduced the incidence of malaria and increased hemoglobin concentrations [11],[12]. However, this strategy was never widely adopted because of logistical constraints and the possibility that such chemoprophylaxis could hinder the development of antimalarial immunity and select for drug-resistant parasites [11],[12]. More recently, attention has focused on intermittent preventive therapy (IPT), the administration of therapeutic doses of an antimalarial drug at predefined times regardless of an individual's infection status, an approach that is widely recommended for pregnant women, but of less certain value for children [13]. Indeed, many years after the concept of chemoprevention for high risk groups in endemic areas was abandoned, this concept needs to be revisited, particularly in light of the renewed interest in malaria elimination.

The most studied strategy for IPT in African children is the administration of SP at the time of routine vaccinations, usually three treatments in the first year of life (IPTi). In a pooled analysis of six randomized controlled trials, IPTi with SP was associated with a protective efficacy of 30% against malaria, 21% against anemia, and 23% against all-cause hospitalizations [2]. However, a subsequent study conducted from 2004 to 2008 in Tanzania reported no protective efficacy of IPTi with SP [14]. Considering these results and concerns regarding drug resistance, in 2010 the WHO recommended IPTi with SP only in areas with moderate-to-high malaria transmission and limited parasite resistance to SP, defined as a prevalence of the *pfdhps* K540E mutation of ≤50% [15]. Of note, following these guidelines, IPTi would not be recommended in most of east Africa, including Uganda, where the prevalence of the key *pfdhps* mutation is very high [16]. Other drugs have also been studied for IPTi, with protective efficacies against falciparum malaria of 38% for mefloquine in Tanzania (although this drug was not well tolerated) [14], 26% for both SP+artesunate (AS) and AQ+AS in Kenya [17], and 35% for SP+AQ and 31% for SP+AS in Papua New Guinea [18].

In parts of west Africa—where malaria is seasonally restricted, the main burden is in older children, and the prevalence of SP resistance is low—a different approach, SMC, has been studied [5],[16]. In a number of studies, generally utilizing monthly SP+AQ in children under 5 y of age, protective efficacies against malaria ranged from 31% to 93% [5]. SMC has recently been recommended as a malaria control strategy for the Sahel sub-region by the WHO [19], and delivery by trained community health workers has been successful in several countries [12]. However, as noted above, SMC with SP+AQ is not appropriate for most of east Africa, and in these regions it is not clear what alternative chemoprevention strategies are appropriate. Indeed, the current armamentarium of drugs appropriate for chemoprevention is limited and dependent on compounds developed for treatment rather than prevention. Drug development programs should place a greater emphasis on drugs for chemoprevention, considering pharmacokinetic and pharmacodynamic properties unique to this indication.

In this study, chemoprevention interventions were tailored to an area of east Africa with intense, year-round malaria transmission, with regular administration from 6 to 24 mo of age. Two intensive antifolate regimens were studied, monthly SP and daily TS, providing much more consistent drug exposure than the infrequent IPTi regimens previously studied in east Africa. Despite the more intensive dosing, SP offered no significant protection against malaria, but an excess risk of moderate–severe anemia, and TS was associated with only 28% protective efficacy. Although noncompliance with study drugs likely contributed to a reduction in the protective efficacy, these results and others [16] suggest that alternatives to antifolates are needed for malaria chemoprevention, especially in east Africa. Indeed, in a recent study from the same area of Uganda, over 90% of children with malaria were infected with parasites containing five *pfdhfr/pfdhps* mutations that together have been strongly correlated with antifolate resistance [3]. The finding of an excess risk of anemia in the monthly SP arm raises concerns that in areas of high antifolate resistance this regimen may actually be harmful. In studies from areas with high antifolate resistance in Tanzania, IPTi with SP was associated with a trend towards an excess risk of anemia in infants [14], and IPT in pregnancy with SP was associated with an excess risk of fetal anemia [20]. In contrast, monthly DP was safe and highly protective against malaria, moderate–severe anemia, asymptomatic parasitemia, and gametocytemia. Of particular note was the lack of evidence of cardio-toxicity despite repeated dosing with DP, confirming results of a previous study from Thailand and contradicting current recommendations of the European Regulatory Agency warning of the risk of prolonged QTc intervals with DP [21]. These results are not surprising given the well-demonstrated excellent treatment efficacy and extended post-treatment prophylactic effect of DP, which benefits from the long half-life of PQ [22]. Chemoprevention with DP has previously shown promising results in two studies. In Senegal, monthly DP during the transmission season was well tolerated and as effective as SP+AQ for the prevention of malaria in children, although protective efficacy could not be estimated because of the lack of a control group [23]. In Thai adults, the protective efficacy of DP was 98% when given monthly and 86% when given every 2 mo [24]. The most important determinant of protective efficacy in this study was the trough plasma concentration of PQ, and it was concluded that for chemoprevention the drug should be administered monthly.

The impact of chemoprevention on the development of naturally acquired immunity to malaria was also of interest in this study. Results from prior studies of the impact of chemoprevention on the incidence of malaria in the period after the intervention was stopped have been mixed. In a study from Tanzania, chemoprophylaxis with weekly pyrimethamine plus dapsone in children 8–48 wk of age was highly efficacious, but associated with a “rebound” effect leading to an increased incidence of malaria in the year after the intervention was stopped, suggesting that continuous chemoprophylaxis impaired the development of naturally acquired immunity [25],[26]. In contrast, in a pooled analysis of six randomized controlled trials, IPTi with SP was not associated with a rebound in the incidence of malaria in the 5 mo following the intervention [2]. Indeed, in the first study of IPTi with SP in Tanzania, SP showed the highest protective efficacy during the intervention, and there was a sustained reduction in the incidence of malaria in the year after the intervention was stopped, suggesting that IPTi could actually enhance the development of naturally acquired immunity [27]. In a more recent study from Mozambique, chemoprevention with monthly SP+AS between 2.5 and 4.5 mo of age or between 5.5 and 9.5 mo of age was highly effective and was associated with a trend towards a higher risk of malaria in the second year of life compared to children who had not received chemoprevention, but the differences were not statistically significant [28]. In this study, the incidence of malaria rose from 6 to 36 mo of age in the no chemoprevention arm, reaching a remarkably high level, suggesting the lack of acquisition of clinically significant naturally acquired immunity and/or a temporal increase in exposure. In the year following the discontinuation of study drugs, the incidence of malaria for children randomized to the three chemopreventive regimens was similar to that of children who had not received prior chemoprevention. The results suggest that in this high transmission setting, the use of chemopreventive regimens with a range of protective efficacies had little impact on the development of naturally acquired immunity.

There were several limitations of this study. Administration of study drugs was not directly observed. Rather, study drugs were administered at home in a way that could be standardized across study drug regimens. Although attempts were made to measure compliance through the use of adherence calendars, pharmacokinetic data from the DP arm suggested that compliance was much lower than what was reported by parents/guardians. The study also had multiple study arms with multiple primary and secondary outcomes. Given the number of comparisons made, caution should be taken in interpretation of significance because of the increased risk of type-1 errors. Finally, this study was conducted in an area of high transmission intensity and widespread antifolate resistance; therefore, results may not be generalizable to other settings.

Our results lead to some key questions. First, considering the long exposure to PQ after monthly dosing [10], the outstanding efficacy demonstrated in Thailand [24], and the lack of evidence of resistance to dihydroartemisinin or PQ in Uganda [29], why wasn't the preventive efficacy of DP even higher than 58%? The most likely explanation is non-adherence to a regimen that was administered at home without supervision, and the observed decreasing efficacy over time might be explained by decreasing adherence over the course of the study. In addition, pharmacokinetic factors may have played a role as PQ exposure has been shown to be lower in young children, and an increased dose regimen may be required to achieve the same post-treatment prophylactic effect as in older age groups [10]. Second, did monthly DP select for parasites resistant to the components of the drug? Studies to answer this question are underway; preliminary results suggest that the ex vivo sensitivities of parasites isolated from children receiving DP were not different from those of parasites isolated from children not receiving chemoprevention (P. J. Rosenthal, unpublished data). Further, in Burkina Faso prior therapy with DP did not select for parasite polymorphisms selected by the related aminoquinolines chloroquine and AQ [30], suggesting that, despite a reported history of resistance in China [31], PQ may not readily select for resistant parasites. Lastly, are our results demonstrating strong protective efficacy for DP relevant to other areas? In a 2009 malaria indicator survey, parasite prevalence was 38% in children under 5 y in the eastern region of Uganda, including Tororo, similar to the national average of 43% [32]. Indeed, the most recent data from the Malaria Atlas Project from 2010 classifies a majority of Uganda (including Tororo) as being in the highest level of endemicity, defined as a parasite rate of >40% among children 2–10 y of age [33]. Thus, our results are likely representative of those to be expected in most of Uganda. Indeed, despite well-documented successes in reducing the burden of malaria in several African countries [1], the burden remains remarkably high in Uganda, and new control interventions are urgently needed [34].

The excellent efficacy of DP for the treatment of malaria in multiple countries [22] and for chemoprevention in our high transmission area suggest that this regimen will offer benefit in many regions in need of improved control measures. However, some caveats should be pointed out when considering DP for chemoprevention. Monthly dosing of a drug given over 3 d would pose considerable challenges in implementation and cost. For example, the cost of a 1-y supply of study drugs for the average size child during the intervention in this study was US$0.47 for monthly SP, US$2.16 for daily TS, and US$12.00 for monthly DP (based on local market prices). Additionally, widespread use of an ACT for chemoprevention could compromise the efficacy of the drug when used for treatment. One strategy would be the use of ACTs with different partner drugs for prevention and treatment, in particular utilizing drugs that select for different resistance determinants. For example, in a recent longitudinal randomized trial in Uganda, recent AL treatment selected for *pfmdr1* genotypes associated with decreased sensitivity to AL, while DP selected for opposite genotypes [35]. In light of these considerations, future studies are needed to evaluate the preventive efficacy of DP in other areas, to maintain surveillance for potential selection of drug-resistant parasites, and to evaluate the role of chemoprevention in the context of other available malaria control interventions such as the scale-up of ITNs and use of indoor residual spraying of insecticides. In addition, there are numerous programmatic barriers that will need to be addressed such as developing appropriate delivery systems (e.g., use of community health workers), ensuring compliance and proper dosing, and, perhaps most challenging, providing adequate funding in an environment where resources are already scarce.

## Supporting Information

Checklist S1 CONSORT checklist.
(DOC)

Protocol S1 Promote–Chemoprevention protocol.
(PDF)

Text S1 Institutional review board approval from Makerere University School of Medicine Research and Ethics Committee.
(PDF)

Text S2 Institutional review board approval from the Uganda National Council for Science and Technology.
(PDF)

Text S3 Institutional review board approval from the University of California, San Francisco.
(PDF)

## Abbreviations

ACT: artemisinin-based combination therapy
AL: artemether-lumefantrine
AQ: amodiaquine
AS: artesunate
DP: dihydroartemisinin-piperaquine
ECG: electrocardiogram
IPT: intermittent preventive therapy
IPTi: intermittent preventive therapy in infants
ITN: insecticide-treated bednet
PE: protective efficacy
PQ: piperaquine
SMC: seasonal malaria chemoprevention
SP: sulfadoxine-pyrimethamine
TS: trimethoprim-sulfamethoxazole
