# Supplementary material for: Protective Efficacy and Safety of Three Antimalarial Regimens for the Prevention of Malaria in Young Ugandan Children: A Randomized Controlled Trial
Source: PLoS Med. 2014 Aug 5;11(8):e1001689. doi: 10.1371/journal.pmed.1001689 (PMC4122345; doi:10.1371/journal.pmed.1001689)
Supplement: Protocol S1 — Promote–Chemoprevention protocol. (PDF) [file pmed.1001689.s002.pdf]

“PREVENTION OF MALARIA AND HIV DISEASE IN TORORO” (PROMOTE)

A UCSF/ MAKERERE UNIVERSITY COLLABORATION

“PROMOTE – CHEMOPREVENTION”

A RANDOMIZED CONTROLLED TRIAL OF MONTHLY DIHYDROARTEMISININ-PIPERAQUINE VERSUS MONTHLY SULFADOXINE-PYRIMETHAMINE VERSUS DAILY TRIMETHOPRIM-SULFAMETHOXAZOLE VERSUS NO THERAPY FOR THE PREVENTION OF MALARIA

VERSION 6.0

Sponsored by:

The National Institute of Child Health and Human Development

Principal Investigators:	Grant Dorsey, MD, PhD<br>University California San Francisco
	Moses Kamya, MBChB, MMed, MPH<br>Makerere University
Co-investigator:	Philip Rosenthal, MD<br>University California San Francisco

Protocol Version 6.0, March 6, 2012

TABLE OF CONTENTS

PROTOCOL TEAM ROSTER 6

GLOSSARY 9

SCHEMA 10

1. INTRODUCTION 11

1.1. Background 11

Table 1. Summary of studies evaluating chemoprophylaxis in infants and children 14

Table 2. Summary of studies evaluating IPTi with SP given at the time of routine immunizations 16

1.2. Preliminary studies 16

Table 3. Results of MU-UCSF antimalarial drug efficacy trials 17

Figure 1. Risk of recurrent parasitemia following therapy with AL vs. DP 17

Figure 2. Prevalence of key antifolate resistance-conferring mutations over time 19

Figure 3. Age distribution of patients < 5 years of age with malaria in Tororo 20

1.3. Rationale 21

2. STUDY OBJECTIVES 22

2.1. Objective 1 22

2.2. Objective 2 23

2.3. Objective 3 23

3. STUDY DESIGN 23

Figure 4. Study profile 25

4. SELECTION AND ENROLLMENT OF SUBJECTS 25

4.1. Inclusion Criteria 25

4.2. Exclusion Criteria 26

4.3. Initial Screening 26

4.4. Study Enrollment Procedures and Baseline Evaluation 26

5. STUDY TREATMENT 27

5.1. Treatment Group Assignments 27

5.2. Treatment Allocation 28

5.3. **Study Drug Dosing and Formulations** ... 28
Table 5. Drug formulation and labeling ... 29
5.4. **Study Drug Administration and Duration** ... 29
5.5. **Study Drug Supply, Distribution, and Pharmacy** ... 29
5.6. **Study Drug Accountability** ... 30
5.7. **Additional Medications** ... 30

6. SUBJECT MANAGEMENT ... 30

6.1. **Subject Follow-up** ... 30
6.2. **New Visits for Medical Problems** ... 31
6.3. **Diagnosis of Malaria** ... 31
6.4. **Management of Uncomplicated Malaria** ... 31
6.5. **Management of Complicated Malaria** ... 32
Table 6. Follow-up schedule for malaria treatment episodes up to two years of age ... 32
Table 6a. Follow-up schedule for malaria treatment episodes after two years of age ... 33
6.6. **Malaria Treatment Outcome Classification System** ... 33
6.7. **Management of Clinical Treatment Failures** ... 33
6.8. **Management of Non-Malaria Illnesses** ... 34
6.9. **After Hours Visits** ... 34
6.10. **Routine Assessments** ... 34
Table 7. Follow-up schedule for routine assessments ... 36
6.11. **Medical Care Outside the Study Clinic** ... 36
6.12. **Criteria for premature subject discontinuation** ... 36
6.13. **Diagnostic and Laboratory Testing** ... 37
6.13.1. **Microscopy** ... 37
6.13.2. **Clinical Laboratory Studies** ... 37
6.13.3. **Molecular, Parasitology, Immunology, and PK Studies** ... 37
6.13.4. **Planned tests on banked specimens** ... 39
6.14. **Nutritional Visits ... 39**
6.15. **Household questionnaire ... 39**

7. MANAGEMENT OF TOXICITIES RELATED TO STUDY MEDICATIONS ... 40

7.1. **Grade 1 or 2 Toxicities** ... 40
7.2. **Grade 3 or 4 Toxicities** ... 40

7.3 ECG Related Toxicities 41

8. MONITORING OF ADVERSE EVENTS AND MANAGEMENT 41

8.1. Monitoring and Reporting of Adverse Events 41

8.1.1. Definitions 41
8.1.2. Identification of Adverse Events 41
8.1.3. Reporting of Adverse Events 42
Table 8. Guidelines for reporting adverse events 43

9. STATISTICAL CONSIDERATIONS 43

9.1. Hypothesis 1 43

9.1.1. Primary Outcome 44
9.1.2. Secondary Outcomes 44
Table 9. Secondary outcomes 44
9.1.3. Analyses 44
9.1.4. Sample Size and Accrual 45

9.2. Hypothesis 2 46

9.2.1. Primary Outcome 46
9.2.2. Secondary Outcomes 46
9.2.3. Analyses 47
9.2.4. Sample Size and Accrual 47
Table 10. Relative differences in incidence of adverse events detectable 47

9.3. Hypothesis 3 47

9.3.1. Primary Outcome 48
9.3.2. Secondary Outcomes 48
9.3.3. Analyses 48
9.3.4. Sample Size and Accrual 48

9.4. Data and Safety Monitoring Plan 49

9.4.1. Data and Safety Monitoring Board 49
9.4.2. Interim Analyses and Stopping Guidelines 49
Table 11. Schedule of interim analyses and boundaries to monitor study outcome 49

9.5. Rationale, Sample Size, and Analysis Plan for Parasite Culturing and Post-Malaria Phlebotomy 50

10. DATA COLLECTION AND MONITORING 51

10.1. Record Keeping 51

10.2. Data Quality Assurance and Monitoring 52

11. HUMAN SUBJECTS 52

11.1. Subject Selection Criteria 52

11.2. Risks and Discomforts 52

11.2.1. **Privacy** ... 52
11.2.2. **Finger Pricks, Heel Sticks, and Venipuncture** ... 53
11.2.3. **Risks of Study Medications** ... 53

11.3. **Treatment and Compensation for Injury** ... 55

11.4. **Costs to the Subjects** ... 56

11.5. **Reimbursement of Subjects** ... 56

11.6. **Institutional Review Board (IRB) Review and Informed Consent** ... 56

11.7 **Definition of Parent/Guardianship.** ... 56

11.8 **Study Discontinuation** ... 57

12. PUBLICATION OF RESEARCH FINDINGS ... 57

13. BIOHAZARD CONTAINMENT ... 57

14. REFERENCES ... 57

15. APPENDICES ... 63

Appendix A. Information Sheet ... 63

Appendix B. Research Participant Main Informed Consent ... 64

Appendix C. Informed Consent for Future Use of Biological Specimens ... 74

Appendix D. Weight-based Administration of Study Drugs ... 77

Appendix E. WHO Criteria for Severe Malaria and Danger Signs ... 78

Appendix F. WHO Antimalarial Treatment Outcome Classification System ... 79

Appendix G. Schedule of Clinical and Laboratory Evaluations ... 80

Appendix H. Guidelines for Adverse Event Grading (DAIDS AE grading table for symptoms and DMID AE grading table for laboratory tests) ... 81

Appendix I. List of drugs used in children and available in Uganda known to cause prolongation of the QT interval ... 87

Appendix J. Food insecurity and water and sanitation questions for Nutrition Interview (version March 31, 2011) ... 88

Appendix K. Nutrition questions for focus group discussion or open-ended interviews (Version March 31, 2011) ... 91

Appendix L. Informed Consent to Participate in a Nutrition Interview or Focus Group 93

Appendix M. Chemoprevention Acceptability Questionnaire ... 96

Appendix N. Informed Consent to Participate in Chemoprevention Acceptability Questionnaire ... 97

Appendix O. Household Questionnaire ... 100

Appendix P. Household Questionnaire Verbal Consent script ... 109

PROTOCOL TEAM ROSTER

Investigators

Grant Dorsey, MD, PhD University of California, San Francisco
Address: UCSF, Parnassus Avenue Box 0811, San Francisco, CA 94143, U.S.A.
Phone Number: 01-415-206-4680
Fax Number: 01-415-648-8425
Email: gdorsey@medsfgh.ucsf.edu

Philip Rosenthal, MD, University of California, San Francisco
Address: UCSF, Parnassus Avenue Box 0811, San Francisco, CA 94143, U.S.A.
Phone Number: 01-415-206-8845
Fax Number: 01-415-648-8425
Email: prosenthal@medsfgh.ucsf.edu

Jane Achan, MBChB, MPed, Makerere University, Kampala, Uganda
Address: MU-UCSF Research Collaboration,
P.O. Box 7475, Kampala, Uganda
Phone Number: +256-414-530692
Fax Number: +256-414-540524
Email: achanj@yahoo.co.uk

Bryan Greenhouse, MD, University of California, San Francisco
Address: UCSF, Parnassus Avenue Box 0811, San Francisco, CA 94143, U.S.A.
Phone Number: 01-415-206-8844
Fax Number: 01-415-648-8425
Email: bgreenhouse@medsfgh.ucsf.edu

Maggie Feeney, MD, University of California, San Francisco
Address: UCSF, Parnassus Avenue Box 1234, San Francisco, CA 94143, U.S.A.
Phone Number: 415-206-8218
Email: feeneym@medsfgh.ucsf.edu

Sera Young, PhD
Address: Cornell University, Division of Nutritional Sciences, Savage Hall, Ithaca, NY 14853
Phone Number: 607 351 0172
Fax Number: 607 255 1033
Email: sly3@cornell.edu

OTHER PERSONNEL

Name	Title	Institution
Dr. Victor Birgira	Senior Study Coordinator	IDRC, Uganda
Dr. James Kapisi	Junior Study Coordinator	IDRC, Uganda
Dr. Bridget Nzarubara	Regulatory Affairs Manager	IDRC, Uganda
Beth Osterbauer	Project Coordinator	UCSF
Barnabas Natamba	Nutritionist	Cornell University
Dr. Rebecca Stoltzfus	Nutritionist	Cornell University

Core Directors, Internal Scientific Steering Committee

Diane V. Havlir, MD, University of California, San Francisco
Address: San Francisco General Hospital, 995 Potrero Avenue,
Building 80, Ward 84, UCSF Box 0874, San Francisco, CA 94110, U.S.A.
Phone Number: 01-415-476-4082, ext 400
Fax Number: 01-415-502-2992
Email: dhavlir@php.ucsf.edu

Moses R. Kamya, MBChB, MMed, MPH, Makerere University, Kampala, Uganda
Address: Department of Medicine, P.O. Box 7072, Kampala, Uganda
Phone Number: +256-414-533200
Fax Number: +256-414-540524
Email: mkamya@infocom.co.ug

Deborah Cohan, MD MPH, University California San Francisco
Address: San Francisco General Hospital, 995 Potrero Avenue, Box SFGH ,
SFGH 6D22, San Francisco, CA. 94143 - SFGH
Phone Number: 01-415-206-3658
Fax Number: 01-415-206-3112
Email: cohand@obgyn.ucsf.edu

Pius Okong, MBChB**,** Dip Obs, MMed, PhD, San Raphael of St. Francis Hospital
Nsambya, Uganda
Address: San Raphael of St. Francis Hospital Nsambya, P.O.Box 7146, Kampala,
Uganda
Phone Number: +256 414-266350
Fax Number: +256 414-267870
Email: pio_okong@yahoo.co.uk

Edwin Charlebois, PhD, University of California, San Francisco
Address: 50 Beale St., 13th Floor, San Francisco, CA 94105, U.S.A.
Phone Number: 01-415-597-9301
Fax Number: 01-415-597-9213
Email: edwin.charlebois@ucsf.edu

Theodore Ruel, MD, Fellow, Division of Infectious Disease, Department of Pediatrics
Address: University of California, San Francisco, 500 Parnassus Avenue, MU4E
Campus Box 0136, San Francisco, CA 94143-0136
Phone Number: 01-415 476 9198
Fax Number: 01-415 476 1343
Email: ruelt@peds.ucsf.edu

Tamara Clark, MPH, University of California, San Francisco
Address: UCSF, Parnassus Avenue Box 0811, San Francisco, CA 94143, U.S.A.
Phone Number: 01-415-206-8790
Fax Number: 01-415-648-8425
Email: tclark@medsfgh.ucsf.edu

GLOSSARY

AE	Adverse event
AL	Artemether-lumefantrine
ALT	Alanine transaminase (SGPT)
CAB	Community advisory board
CBC	Complete blood cell count
CDC	Center for Disease Control and Prevention
CRF	Case report form
DP	Dihydroartemisinin-piperaquine
DSMB	Data and Safety Monitoring Board
IRB	Institutional review board
IRS	Indoor residual spraying
ITN	Insecticide treated net
MOH	Ministry of Health
MU	Makerere University
NICHD	National Institute of Child Health and Human Development
NIH	National Institute of Health
SAE	Serious adverse event
SP	Sulfadoxine-pyrimethamine
TASO	The AIDS Support Organization
TDH	Tororo District Hospital
TS	Trimethoprim-sulfamethoxazole
UCSF	University California San Francisco
WHO	World Health Organization

SCHEMA

**Title**	A randomized controlled trial of monthly dihydroartemisinin-piperaquine versus monthly sulfadoxine-pyrimethamine versus daily trimethoprim-sulfamethoxazole versus no therapy for the prevention of malaria
**Description**	Open label, randomized controlled trial
**Study Objectives**	1. To compare the incidence of malaria among infants enrolled at 4-5 months of age and randomized to receive no chemoprevention, daily TS, monthly SP, or monthly DP until they reach the age of 24 months<br>2. To compare the incidence of adverse events among infants enrolled at 4-5 months of age and randomized to receive no chemoprevention, daily TS, monthly SP, or monthly DP until they reach the age of 24 months<br>3. To compare the incidence of malaria among children for 1 year following intervention with no chemoprevention, daily TS, monthly SP, or monthly DP.
**Participants and Sample Size**	200 HIV-exposed infants born to HIV-infected mothers and 400 HIV-unexposed infants born to HIV-uninfected mothers living in an area of high malaria transmission intensity (600 total study participants).
**Clinical Site**	The study will be conducted in Tororo, Uganda. A designated study clinic will be located within the Tororo District Hospital Complex. The study clinic will be open daily from 8:00 am to 5:00 pm and after-hours care will be available at Tororo District Hospital.
**Selection Criteria**	<u>Inclusion Criteria</u><br>1. Age 4-5 months<br>2. Confirmed HIV status of biological mother<br>3. Negative HIV DNA PCR test at time of enrollment for infants born to HIV-infected mothers<br>4. Infants born to HIV-infected mothers must be breastfeeding<br>5. Residency within 30km of the study clinic<br>6. Agreement to come to the study clinic for any febrile episode or other illness and avoid medications given outside the study protocol<br>7. Provision of informed consent by parent or guardian<br><u>Exclusion Criteria:</u><br>1. History of allergy or sensitivity to TS, SP, or DP<br>2. Active medical problem requiring in-patient evaluation at the time of screening<br>3. Intention of moving more that 30km from the study clinic during the follow-up period<br>4. Chronic medical condition (i.e. malignancy) requiring frequent medical attention<br>5. Living in the same household as a previously enrolled study participant<br>6. QTc interval > 450 msec<br>7. Other clinically significant ECG abnormalities such as arrhythmia, ischemia, or evidence of heart failure<br>8. Family history of Long QT syndrome<br>9. Current use of drugs that prolong the QT interval
**Treatment assignment**	HIV-exposed children will be randomized to chemopreventive therapy at the time they have completed breastfeeding and confirmed to have remained HIV-uninfected. HIV-unexposed children will be randomized to chemopreventive therapy at the time they reach 6 months of age. Chemopreventive therapy will be continued until all children reach 24 months of age.
**Treatment arms**	Daily trimethoprim-sulfamethoxazole<br>Monthly sulfadoxine-pyrimethamine (single dose)<br>Monthly dihydroartemisinin-piperaquine (once a day for 3 days dosing)<br>No chemopreventive therapy
**Follow-up and Diagnosis of Malaria**	Study participants will be followed for all of their outpatient medical care in our study clinic until they reach 36 months of age. Routine assessments will be performed in the study clinic for all study participants approximately once every 30 days. Patients presenting with a new episode of fever will undergo standard evaluation for the diagnosis of malaria.
**Malaria Case Definitions**	**Uncomplicated malaria (all of the following):**<br>1. Documented fever or history of fever in the prior 24 hours<br>2. Positive thick blood smear<br>3. Absence of complicated malaria<br>**Complicated malaria (any of the following):**<br>1. Evidence of severe disease with a positive thick blood smear<br>2. Danger signs with a positive thick blood smear<br>3. Parasite density $\geq$ 500,000/ul

1. INTRODUCTION

1.1. Background

Malaria is Africa's leading cause of mortality in children under five years of age. There are several reasons why Africa bears a large proportion of the world's malaria burden. First, most malaria infections in sub-Saharan Africa are due to *Plasmodium falciparum*, the cause of the most difficult to treat and severe form of the disease. Second, this region is also home to the most efficient malaria mosquito vectors. Third, most African countries are "the poorest of the poor", lacking the basic infrastructure and resources necessary to mount sustainable malaria control efforts. Uganda is emblematic of the immense problem that malaria poses for Africa countries. Malaria is endemic in over 95% of the country, with the highest malaria transmission intensities reported in the world [1]. According to a recent report from the World Health Organization, Uganda has the world's highest malaria incidence, with a rate of 478 cases per 1000 population per year [2]. Malaria is the leading cause of morbidity and mortality in Uganda and is responsible for up to 40% of all outpatient visits, 25% of all hospital admissions and 14% of all hospital deaths (Uganda Ministry of Health, unpublished). The overall malaria-specific mortality is estimated to be between 70,000 and 100,000 child deaths annually in Uganda, a death toll that far exceeds that of HIV/AIDS [3]. A 1995 Burden of Disease study indicated that 15% of life years lost to premature death was due to malaria and that families spend 25% of their income on malaria (Uganda Ministry of Health, unpublished). Poor school performance and absenteeism due to malaria reduce chances of escaping from poverty [4]. Poor people tend to live in environments conducive to mosquito breeding and malaria transmission. Thus malaria enhances poverty, which in turn causes poor disease management, locking people in a malaria-poverty trap [5]. Despite the overwhelming burden imposed by malaria in Africa, there is increasing optimism that the tide can be turned through the establishment of several recent large-scale initiatives. The United States government has recently launched the President's Malaria Initiative (PMI), with the goal of reducing malaria-related deaths in selected countries, including Uganda, by 50% within five years. Through PMI and other large funding sources, such as the Global Fund to Fight AIDS, Tuberculosis and Malaria, Uganda and other Africa countries now have an unprecedented opportunity to reduce malaria associated morbidity and mortality on a national scale. Innovative approaches to malaria control targeting high risk populations are urgently needed to achieve these goals.

Malaria in sub-Saharan Africa is characterized by high rates of infection and the development of partial immunity after repeated exposure. Transmission intensity ranges from under ten to several hundred infective bites per person year in most endemic areas, but the majority of these infections do not lead to clinical illness. The risk of developing symptomatic disease is inversely proportionate to the level of acquired immunity. Partial immunity develops through repeated exposure, leading first to protection against severe forms of disease, followed by protection against symptomatic illness [6]. The result of this phenomenon is that the burden of malaria in Africa is heavily borne by young children. Newborns are protected during the first few months of life due to the transplacental acquisition of maternal antibodies and relatively high hemoglobin F content in the infants' erythrocytes [7]. After about 3-4 months of age, protection from

these factors wanes, and the infant becomes much more vulnerable to malaria, with an estimated 75% of malaria deaths occurring in African children under the age of 5 years [8]. However, the age at which malaria risk peaks in endemic areas of Africa varies from 1-2 years of age in areas of high transmission intensity to approximately 5 years of age in areas of low to moderate transmission intensity [9]. When studying malaria incidence and interventions to prevent malaria, it is essential to have a thorough understanding of the local epidemiology of the disease. Our proposed studies will benefit from the extensive experience we have gained studying malaria in Uganda. Lessons learned in Uganda will also be relevant for other African nations.

Proven effective malaria control interventions currently available for children living in Africa include insecticide-treated bed nets (ITNs), indoor residual spraying (IRS) of insecticide, and effective case management. In controlled trials, ITNs have been associated with a 17% reduction in child mortality and a 50% reduction in the incidence of clinical malaria [10]. One challenge for African countries is to increase ITN coverage, especially amongst high risk populations. According to the Ugandan 2006 Demographic and Health survey only 10% of children under the age of 5 years were sleeping under an ITN. Currently, large scale efforts are underway to increase ITN coverage in Uganda, with a goal of reaching 85% of children under 5 years by 2010 (PMI Malaria Operational Plan 2007). Uganda, like many other African countries, has renewed efforts to provide vector control through the use of IRS of insecticide. In 2006, Uganda began a successful IRS program in low endemicity highland areas of the country; however these areas represent less than 5% of the Ugandan population. Recently the IRS program was expanded to one high transmission district, as well as internally displaced peoples' camps in northern Uganda. However, it is unknown whether IRS is effective or sustainable in high transmission settings. The cornerstone of childhood malaria control for much of Africa has been the provision of prompt and effective antimalarial therapy (case management). However, the spread of resistance to inexpensive drugs has seriously compromised this strategy. Newer combination therapies have been shown to be highly effective and have been adopted in most African countries, but these drugs are considerably more expensive than older therapies, and their availability remains limited. As these newer therapies become more widely available, it is important to use them in a rational way to prevent the further spread of drug resistance. Progress in malaria vaccine research has been substantial, with recent reports from Mozambique of a candidate vaccine that provided modest protection in infants and children [11, 12]. However, large scale phase III trials have yet to be initiated, and it is likely that a vaccine for routine use is at least 5-10 years away. Even if implemented, available vaccines will likely only offer partial protection against malaria. Based on the considerations noted above, there is no clear single path for improved malaria control in Africa. Rather, improvements in control will likely come from incremental advances involving better and more widespread use of proven interventions. For the first time, resources are becoming available to fund the expansion of existing interventions and implement new strategies previously thought to be prohibitively expensive. One such strategy is the use of antimalarial drugs for the prevention of malaria in high risk populations, in particular infants and young children. Research is urgently needed to guide policy regarding which chemopreventive strategies are optimal across the wide spectrum of malarial epidemiology.

Antimalarial drugs have long been used to prevent illness and reduce transmission. With evolution of preventive strategies, the balance of efficacy and long-term feasibility has led to progressively more focused use of drugs. Thus, mass drug administration and broad use of chemoprophylaxis in a population have been reduced to targeted drug administration to maximize benefits and to reduce costs, poor adherence, and potential selection of drug resistance. Recently, there has been renewed interest in chemopreventive therapy in infants and young children in Africa, the group that suffers the greatest burden of malaria. Chemoprevention can be divided into two categories, chemoprophylaxis and intermittent preventive therapy (IPT). Chemoprophylaxis is the use of sub-therapeutic doses of drug at frequent intervals. The goal of chemoprophylaxis is to provide sustained drug levels to prevent the development of disease for the duration that the drug is given. IPT is defined as the use of full treatment doses of antimalarial drugs at a few pre-specified time points not linked to symptoms or documentation of infection. The goal of IPT is to reduce the burden of disease in targeted high risk populations. Unlike chemoprophylaxis, with IPT the intervals between doses are typically longer than the time to clear the drug from the blood, allowing for the possibility of infection between doses. It is suggested that this strategy allows the development of protective immunity despite a decrease in malarial incidence.

A number of randomized trials have evaluated the protective efficacy of chemoprophylaxis in African infants and children over the last 20 years (Table 1). In a study from Ethiopia, weekly prophylaxis with CQ was not protective against malaria, likely due to resistance to this drug [13]. Several studies examining prophylaxis with the antifolate class of drugs (pyrimethamine-dapsone or chlorproguanil) reported mixed results, with protective efficacy against clinical malaria ranging from 42-87% [14-17]. A more recent study reported excellent preventative efficacy (97%) using daily atovaquone-proguanil, however, this drug is considered too expensive for routine use in African children and the study was primarily designed to examine the effect of chemoprophylaxis on the immunogenicity of common vaccines used in travelers [18]. Despite positive results from some of these studies, routine chemoprophylaxis has never been widely implemented in African populations due to logistical and economic constraints, as well as the concern for “rebound” of malaria incidence following chemoprophylaxis. As evidence supporting this last concern, in studies from Tanzania and Gambia, children receiving pyrimethamine-dapsone chemoprophylaxis had an 80% and 52% higher incidence of clinical malaria, respectively, compared to placebo arms in the year following the intervention [16, 19].

More recently, several studies have documented the benefits of chemoprophylaxis with TS among African patients infected with HIV. In 1999, results from clinical trials in Cote d’Ivoire showed that TS reduced mortality in adults with pulmonary tuberculosis and lowered hospital admission rates in adults with high CD4 counts without tuberculosis [20, 21]. In an area of Zambia with high levels of bacterial resistance to TS, prophylaxis was associated with a significant decrease in mortality in children across all CD4 strata [22]. In a rural area of Uganda, TS prophylaxis in children and adults was associated with reduced morbidity and mortality and had beneficial effects on CD4 counts and HIV viral loads [23]. Although TS chemoprophylaxis in HIV-infected patients was initially studied as a method of preventing a wide range of opportunistic infections, it is now clear that this intervention is highly effective for the prevention of malaria. In the study from rural

Uganda (the same area as this proposal), the incidence of malaria was approximately 5-fold lower in children and adults taking TS prophylaxis compared to controls [23]. Our group has reported similar protective efficacy of TS prophylaxis against malaria among HIV-infected children living in an urban area in Uganda [24]. TS chemoprophylaxis is now widely recommended for HIV-infected Africans, and as a result HIV-infected patients using this intervention are at much lower risk of malaria compared to their HIV-uninfected counterparts not receiving chemoprophylaxis. In the only study to examine TS chemoprophylaxis among HIV-uninfected patients, this intervention was associated with a 99% protective efficacy against clinical malaria in a cohort of children during seasonal transmission in Mali [25].

Table 1. Summary of studies evaluating chemoprophylaxis in infants and children

Study parameter	Country and year(s) of recruitment					
	Ethiopia 1988 [13]	Mozambique 1989 [15]	Gambia 1983 [14]	Kenya 1986 [17]	Tanzania 1995 [16]	Gabon 2000 [18]
Transmission	perennial	seasonal	seasonal	perennial	perennial	seasonal
Age at enrollment (years)	1-14	7-12	0.3-5	6-10	0.15	4-16
Intervention drug*	CQ	PD	PD	CP	PD	AP
Dosage	weekly	weekly	every 2 wks	weekly	weekly	daily
Duration of intervention	10 weeks	1 year	3 years	20 weeks	40 weeks	12 weeks
Incidence in placebo group†	43%‡	0.28	0.06	3.60	1.34	1.01
# enrolled, placebo/intervention	998/999	197/195	300/300	37/78	411/421	165/165
Preventive efficacy, % (95% CI)						
Clinical malaria	4 (-7-13)	87 (72-95)	80 (31-97)	42 (5-64)	61 (48-70)	97 (79-100)
Hospital admission	not reported	not reported	not reported	not reported	39 (25-49)	not reported
Anemia	not reported	not reported	48 (14-68)	not reported	57 (43-68)	not reported

* CQ=chloroquine; PD=pyrimethamine/dapsone; CP=chlorproguanil; AP=atovaquone/proguanil
† episodes of clinical malaria per person year; ‡ Prevalence of at least one episode of clinical malaria

The most established use of IPT is the provision of SP linked to routine antenatal visits during pregnancy. This intervention has been shown to reduce maternal anemia, placental malaria, and low birth weight, and has been widely adopted in many African countries [26]. The concept of targeted use of antimalarial therapy linked to routine health-care visits led to the evaluation of IPT in infants, who have limited immunity to malaria and are at great risk of anemia and rapid progression to severe disease and death. Initial studies of IPT in infants (IPTi) have focused on the use of SP at the time of routine vaccinations through WHO's Expanded Program on Immunization. Several centers of malaria research formed the IPTi Consortium to conduct a robust and comprehensive assessment of this promising new intervention in order to guide future research and policy development (http://www.ipti-malaria.org/). To date 6 randomized controlled trials have been published evaluating the use of IPTi with SP at the time of routine immunizations (Table 2). The preventive efficacy of IPTi with SP against the incidence of clinical malaria ranged from 17-62%, with statistically significant reductions in 5 of 6 trials. The risk of hospitalization was reduced by 9-30%, with significant reductions in 3 of the 5 studies, and the risk of anemia was reduced by 7-50% with significant reductions in 3 of the 6 studies. In a pooled analysis of these 6 trials, approximately 4,000 infants have received 12,000 doses of IPTi with SP (http://www.ipti-malaria.org/). The drug was

found to be well tolerated and safe, with significantly fewer serious adverse events in the SP group compared to the placebo group. Data from two of the trials have shown that IPTi with SP does not have an adverse impact on the serological responses to common childhood vaccinations [27]. There was no significant rebound in episodes of clinical malaria, anemia, or hospital admissions with malarial parasitemia in the pooled analysis for the 5 month period after the IPTi schedule was finished. However, in individual studies there was rebound in high density parasitemia [28], anemia [29], and severe malaria and severe anemia [30]. In the trial from Tanzania there was sustained protection against clinical malaria into the second year of life, after the conclusion of IPTi, with infants receiving SP having 36% less malaria than untreated controls in the second year of life.

Despite these encouraging findings, major questions remain about the optimal strategy for IPT in infants and young children. One concern is the heterogeneity of results from the 6 published studies of IPTi using SP during routine immunizations. Following the first study from Tanzania, subsequent studies failed to find the high level of protection observed in the first year of life and the persistence of protection observed in the second year of life. A major difficulty in trying to address this discrepancy is that it is not known how IPTi with SP achieves its protective effect. It has been argued that the benefit of IPTi is dependent more on the prophylactic effect of this long-acting drug than its intermittent eradication of existing infections [7]. The duration of a drug's prophylactic effect is dependent both on the drug's pharmacokinetics and pharmacodynamics. Sulfadoxine and pyrimethamine have half-lives of 7 and 3 days, respectively [32]. The antimalarial effect of SP depends on synergy between the two components, but the effect from one treatment dose can last as long as 60 days with fully sensitive *P. falciparum* [32]. However, as parasite resistance increases, the duration of a post treatment prophylactic effect is presumed to decrease. It has been argued that, for maximal antimalarial protective efficacy, the interval between IPT doses should not be more than one week longer than the time for plasma concentrations to fall from peak to minimum inhibitory concentrations [7]. With an alarming decline in SP antimalarial efficacy in Africa due to the spread of resistant parasites [33], there are serious concerns about the long-term preventive efficacy of established IPTi regimens. These concerns are accentuated in settings of high transmission intensity. Indeed, in the published studies of IPTi with SP the greatest benefits were seen during the first month following dosing, and in areas with lower transmission intensity and the highest coverage with ITNs [34].

Key research priorities for IPTi include the investigation of new drugs and new dosing strategies (http://www.ipti-malaria.org/). In a study from a high transmission area in Tanzania the use of amodiaquine given every 2 months at 3, 5, and 7 months of age was associated with a 65% (95% CI 42-77%) reduction in the incidence of malaria at 9 months of age [35]. In a study from Senegal the use of a combination of SP plus artesunate given monthly over 3 months during the local malaria transmission season in children aged 2-59 months was associated with an 86% (95% CI 80-90%) reduction in the incidence of malaria [36]. These results suggest that more frequent dosing of IPTi, more effective drugs, or perhaps continuous chemoprophylaxis will improve the preventive efficacy, although with increasing efficacy against infection may come increasing risks of drug toxicity or malarial rebound after the completion of

chemoprevention. Clearly, more research is urgently needed to critically assess rational new regimens for chemoprophylaxis and IPTi.

Table 2. Summary of studies evaluating IPTi with SP given at the time of routine immunizations

Study parameter	Country and year(s) of recruitment					
	Tanzania 1999-00 [37]	Ghana 2000-02 [28]	Mozambique 2002-04 [38]	Ghana 2003 [30]	Ghana 2004 [29]	Gabon 2002-05 [39]
EIR*/year	29	418	38	not reported	400	50
Transmission	perennial	seasonal	perennial	perennial	perennial	perennial
Age at dosing, months	2, 3, 9	3, 4, 9, 12	3, 4, 9	3, 9, 15	3, 9, 15	3, 9, 15
Incidence in placebo group†	0.36	1.02	0.43	1.16	1.20	0.16
# enrolled, placebo/intervention	351/350	1242/1243	755/748	600/600	535/535	595/594
Preventive efficacy, % (95% CI)	at 12 mo.	at 15 mo.	at 12 mo.	at 18 mo.	at 18 mo.	At 18 mo.
Clinical malaria	62 (44-75)	25 (14-34)	23 (2-39)	23 (12-32)	20 (11-29)	17 (-24-44)
Hospital admission	30 (8-47)	13 (-5-27)	19 (4-31)	31 (3-51)	9 (-23-34)	not reported
Anemia	50 (8-73)	36 (11-53)	13 (-17-35)	24 (4-39)	7 (-8-20)	22 (-1-40)

* Entomological inoculation rate; † Episodes of clinical malaria per person year

1.2. Preliminary studies

Our collaboration has been one of the most active malaria therapy research groups in the world. Seventeen clinical trials have been completed, involving over 7,500 patients, 9 different treatment regimens, and 8 sites around Uganda (Table 3) [40-48]. These studies have been influential in the rapidly changing approach to antimalarial therapy in Africa. Our collaboration has extensive experience in the standardized assessment of antimalarial drug efficacy and the monitoring of adverse events based on WHO and NIH guidelines. Early clinical trials by our group in Uganda documented high failure rates to widely used monotherapies including chloroquine (CQ), amodiaquine (AQ), and SP. More recent studies have focused on combination antimalarial therapy. These results document a very high failure rate with the combination of CQ + SP, the previously recommended first-line treatment for Uganda. Alternative combination therapies were much more effective, and treatment failure due to drug resistance was uncommon after artemisinin-based combination therapies. However, results from Tororo (the study site for this proposal) showed that even treatment with artemether-lumefantrine (AL), the new recommended first-line treatment for malaria in Uganda, was associated with a 52% risk of recurrent malaria within 4 weeks following therapy. These results highlight the extremely high incidence of malaria among children living in high transmission areas and the failure of effective case management to prevent recurrent disease in high risk populations.

Table 3. Results of MU-UCSF antimalarial drug efficacy trials

Treatment*	Number of studies	Total enrolled	Risk of recurrent malaria median (range)	Risk of recrudescence median (range)†
CQ	2	352	80% (80-89%)	Not available
SP	4	604	46% (30-60%)	29%
AQ	1	147	32%	Not available
CQ+SP	9	1534	69% (17-88%)	41% (25-64%)
AQ+SP	12	2133	32% (1-55%)	14% (1-32%)
SP+AS	1	198	29%	19%
AQ+AS	7	1301	43% (17-75%)	7% (3-13%)
AL	4	815	36% (7-53%)	8% (1-16%)
DP	2	426	28% (12-43%)	5% (2-7%)

* CQ=chloroquine; SP=sulfadoxine-pyrimethamine; AQ=amodiaquine; AS=artesunate; AL=artemether-lumefantrine; DP=dihydroartemisinin-piperaquine

†Genotyping was performed to distinguish recrudescences from new infection.

Clinical trials of DP. DP is a new co-formulated ACT that was approved for use in Uganda in 2005. We have completed two clinical trials in Uganda comparing DP to AL, [47, 49]. These trials were conducted at sites with both low (Kanungu) and high (Apac) malaria transmission intensity, enrolling 835 patients, including 741 children under the age of 5 years. Both drugs were highly efficacious, with a low risk of recrudescence after therapy. Relevant to this proposal is the drugs' post-treatment prophylactic effect up to one month after therapy, as this is the dosing interval we are studying for DP as a chemopreventive therapeutic agent. One month after therapy, risks of recurrent parasitemia with DP were 4% and 11% at the low and high transmission sites, respectively, compared to risks of 18% and 29% with AL ($p<0.0001$ for both comparisons) (Figure 1). In addition, DP was better tolerated than AL at both sites, with no serious adverse events related to DP. Given the excellent efficacy and safety of DP, as well as its extended post-treatment prophylactic effect, we believe it may be the ideal therapy available for the chemoprevention of malaria in high-transmission areas.

Figure 1. Risk of recurrent parasitemia following therapy with AL vs. DP

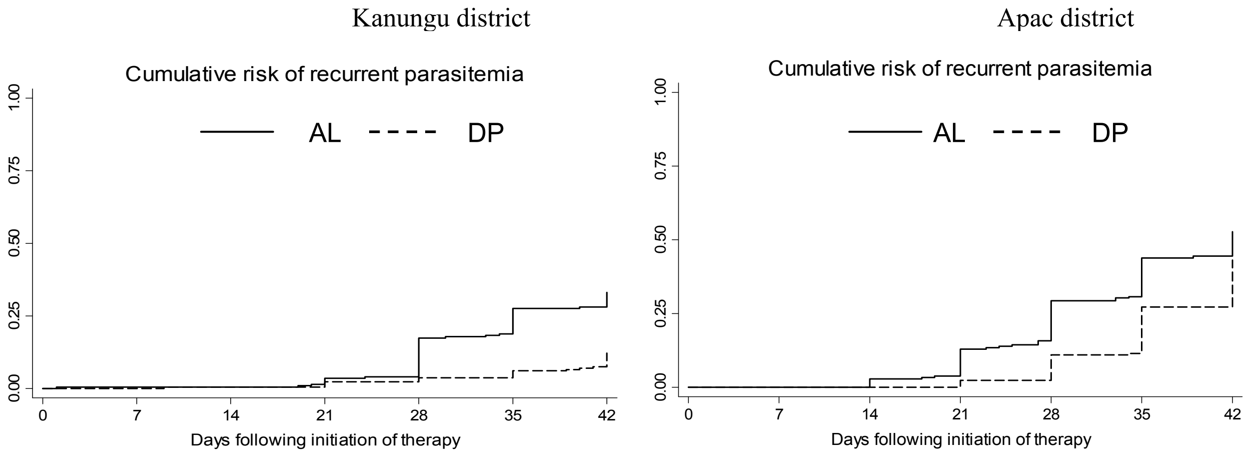

Longitudinal Studies of Malaria Incidence in Uganda. Malaria can strike at any time and must be diagnosed and treated promptly to prevent progression to severe disease. In

Africa, it is common for febrile illnesses to be treated presumptively for malaria without laboratory confirmation. In a research setting, the accurate measurement of malaria incidence requires expertise and a large commitment of time and resources. Our collaboration has been an innovative leader in longitudinal studies of malaria incidence and response to antimalarial therapy in cohorts of children. Valuable lessons that we have learned from these studies include the following. 1) To accurately measure malaria incidence, one needs to provide a setting where study participants can be followed for all of their health care. We have done this by establishing study clinics open 7 days a week with access to care during evening hours. In addition, all medical care is provided free of charge and transportation costs are reimbursed. This system provides a strong incentive for study participants to avoid the use of outside medications (which could interfere with diagnosing malaria) and to seek care in our study clinics for any illness that may be malaria. 2) The study must have the capacity to rapidly and accurately diagnose malaria in all study participants that present with fever. 3) The importance of developing diagnostic and treatment algorithms for common illnesses that allow for standardized approaches to patient care and data collection. 4) The importance of developing quality control measures to maximize diagnostic accuracy and minimize protocol violations. Our studies have been characterized by excellent follow-up rates, with less than 5% loss of person time per year, diagnostic accuracy using light microscopy with a sensitivity and specificity of 99% and 100% respectively [50], and excellent compliance with study protocols, providing prompt malaria diagnosis and treatment and very infrequent self reports of outside antimalarial therapy. The infrastructure we have developed and the experience we have gained will be critical for the successful implementation of the research plan outlined in this protocol.

Antifolate Resistance in Uganda. Surveillance of key mutations in *P. falciparum dhfr* and *dhps* genes, which encode the target enzymes of SP and TS, has been proposed as a means of monitoring antifolate drug resistance in Africa. We have studied the association between the five key mutations commonly reported in Africa and clinical treatment failure in children treated with SP for uncomplicated malaria in Kampala [51, 52]. The prevalences of the *dhfr* 108N (98%) and 51I (95%) mutations were very high, and therefore these mutations were not useful independent predictors of treatment outcome. Considering combinations of mutations, there was generally a "dose response" relationship, with an increasing number of mutations resulting in stronger associations with treatment failure. Infections with parasites containing the quintuple mutant (*dhfr* 108N + 51I +59R; *dhps* 437G + 540E) was associated with over 10 times the odds of treatment failure compared to infections with parasites containing only the 108N and 51I mutations (OR = 10.7, 95%CI 1.8-64.4, p = 0.009).

We have measured the prevalence of key antifolate resistance-conferring mutations from subjects living in Tororo (the site of this proposal) over a 4 year period. From 2002-2006 the prevalence of the *dhfr* triple mutant increased from 40% to almost 80% and the prevalence of the *dhps* triple mutant increased from 60% to almost 100% (Figure 2). This temporal increase in the prevalence of molecular markers of antifolate resistance corresponds to the 2001 implementation of a national policy change from CQ to CQ+SP as the recommended first-line treatment for malaria in Uganda [53]. We have also measured the prevalence of key antifolate resistance-conferring mutations from the 9

children in a cohort of HIV-infected children who developed symptomatic malaria while taking TS prophylaxis [24]. All of these samples contained the *dhfr/dhps* quintuple mutant and one sample contained an additional mutation (*dhfr* 164L) associated with high-level antifolate resistance (probably leading to complete loss of antimalarial activity of antifolates) that is rare in Africa [54] and was only very rarely detected in hundreds of prior samples from Uganda evaluated by our group. These data suggest that TS and SP, currently leading candidates for malaria chemoprevention in Africa, may be faced with diminishing protective efficacy in Uganda. Thus, alternative regimens and strategies, such as DP as proposed in our protocol, should be considered. In addition, our group is positioned to characterize drug resistance in parasites obtained from study participants, providing a more comprehensive picture of current antimalarial drug resistance to guide policy for malaria treatment and prevention.

Figure 2. Prevalence of key antifolate resistance-conferring mutations over time

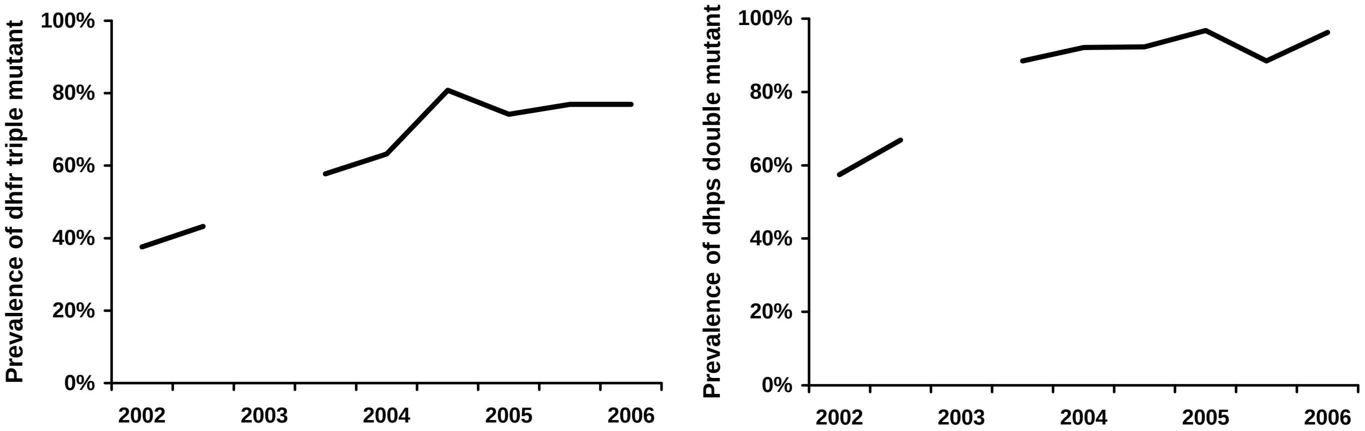

Malaria Studies in Tororo. We have selected Tororo as the site for the studies described in this protocol for several reasons. Tororo is a rural area in Eastern Uganda where malaria is highly endemic and occurs throughout the year. In contrast to our study sites in Kampala, where the number of infective mosquito bites per person year has been estimated to be 4 (MU-UCSF unpublished data), in Tororo the number of infective bites per person year was recently estimated to be 562 [1]. In such high transmission intensity settings, the burden of malaria is concentrated among infants and young children, and if a child survives to the age of 5 years they have generally developed a sufficient degree of immunity such that malaria risk is greatly reduced [6]. This is illustrated by the age distribution of children between the ages of 6 months and 5 years who presented with malaria at a government health clinic in Tororo and have been enrolled in our antimalarial drug efficacy trials. Among over 800 children studied in this age range, almost 80% were less than 2.5 years of age, with a peak in the 1-1.5 year age range (Figure 3). These results differ from those in Kampala, where the incidence of malaria is fairly constant for children aged 1-10 years (MU-UCSF unpublished data). Thus, Tororo provides an ideal epidemiological setting to study the impact of chemopreventive interventions in infants and young children who, in the areas with highest transmission intensity are at the highest risk for malaria.

Figure 3. Age distribution of patients < 5 years of age with malaria in Tororo

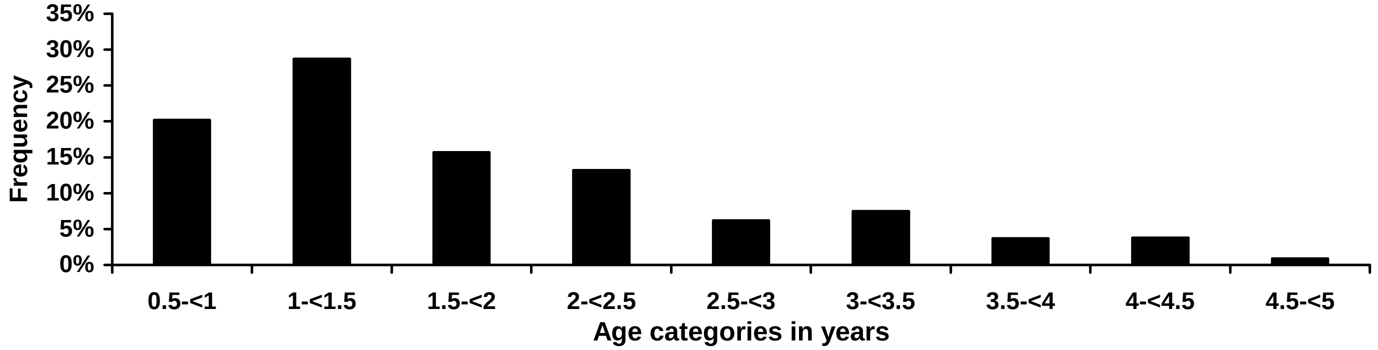

In addition to local epidemiological factors, Tororo is an excellent site for this proposal because of existing infrastructure and preliminary work that has been done by our group and the CDC. The CDC has been working in Tororo since 2001, primarily on studies of HIV and co-infections. They have established large cohorts of HIV-infected patients and their HIV-uninfected family members and developed a large infrastructure to support research based at Tororo District Hospital. The CDC has also established an extensive program to support the prevention of mother to child transmission of HIV, including routine counseling and testing (including PCR based testing). The experience and support provided by the CDC will be critical for assisting in recruitment of study subjects and laboratory services outlined in this proposal.

Recently we began a collaborative study with the CDC to measure malaria incidence in a cohort of HIV-infected and uninfected infants and children. This study has provided us with invaluable experience and preliminary data used for sample size calculations. Briefly, between August 2007 and April 2008 we enrolled 100 HIV-uninfected infants born to HIV-uninfected mothers (HIV-unexposed), 203 HIV-uninfected infants born to HIV-infected mothers (HIV-exposed), and 48 HIV-infected infants. All HIV-infected infants are given TS prophylaxis. HIV-exposed infants are given TS prophylaxis for the duration of breastfeeding and then retested for HIV 6 weeks after breastfeeding is stopped. If the HIV-exposed child remains HIV-uninfected following cessation of breastfeeding, TS prophylaxis is stopped in accordance with national guidelines. If the HIV-exposed child becomes HIV-infected during breastfeeding, TS prophylaxis is continued indefinitely. HIV-unexposed infants are not given TS prophylaxis. All mother-child pairs received a basic care package including ITNs at enrollment. All HIV-infected infants/children receive antiretroviral therapy if eligible according to standardized WHO criteria. Study participants are being followed for all of their health care needs in a designated study clinic. The incidence of clinical malaria through September 2008 among children 6-24 months of age stratified by use of TS prophylaxis is presented in Table 4. Malaria incidence is significantly lower among children taking TS prophylaxis compared to both HIV-exposed children who were never given TS prophylaxis and HIV-exposed children in whom TS prophylaxis was discontinued following cessation of breastfeeding. However, even in the setting of TS prophylaxis and ITN use the incidence of malaria after 6 months of age remains almost 2 episodes per year. These findings suggest that our high transmission study area provides an ideal setting to evaluate new strategies for malaria prevention and provide valuable estimates for our sample size calculations.

Table 4. Malaria incidence in infants and young children living in Tororo

Study group[†]	Age 6-24 months		
	Episodes of malaria	Person time (yrs)	Incidence*
HIV-unexposed not taking TS prophylaxis	417	95.7	4.36
HIV-exposed not taking TS prophylaxis	182	47.8	3.81
Taking TS prophylaxis	256	138.6	1.85

[†] All children given an ITN at enrollment ***** Episodes of malaria per person year

1.3. Rationale

Young African children living in high transmission areas suffer the greatest burden of malaria. In most African countries, such as Uganda, the only current preventive measure against malaria in high transmission settings is the use of ITNs. Our preliminary data show that even in the setting of ITN use, young children living in a high transmission setting suffer almost 4 episodes of clinical malaria per year, highlighting the need for new preventive strategies. Chemopreventive strategies offer a potential means of reducing the burden of malaria among young children living in a high transmission setting. In this study, we will compare the efficacy and safety of 3 promising chemopreventive strategies with the current standard of no chemoprevention. One treatment arm will consist of daily TS, a strategy which has been show to be effective and is now the standard of care in HIV-infected children [24]. Another treatment arm will consist of monthly SP. IPT with SP in infants has been shown to be effective and is currently undergoing large scale Phase III trials in Africa (http://www.ipti-malaria.org/). We have chosen a monthly dosing strategy for SP in this study because previous studies have shown that the greatest benefits were seen during the first month following dosing [34]. Another treatment arm will consist of monthly dosing of the new ACT regimen, DP. We have included this treatment arm because previous studies have shown this drug to be highly effective for the treatment of malaria, with excellent post-treatment prophylaxis over the month following therapy [47, 49].

This study will be conducted in two distinct patient populations: 1) HIV-unexposed children (HIV-uninfected children born to HIV-uninfected mothers) and 2) HIV-exposed children (HIV-uninfected children born to HIV-infected mothers). The rationale for including both of these populations is that there are importance difference in the current standard of care which could modify and effect of our intervention, and therefore the implications of our results. The current standard of care for HIV-unexposed children living in endemic areas of Africa is not to provide chemopreventive therapy. These children are thought to be relatively protected from developing symptomatic malaria during the first few months of life due to the presence of fetal hemoglobin and placental transfer of maternal antibodies. Therefore, the intervention will begin in HIV-unexposed children when they reach 6 months of age, the time at which the incidence of malaria begins to increase. The intervention will be continued until the children reach 24 months of age, which our prior data suggest is when the incidence of malaria begins to decline due to the development of semi-immunity. In addition we will follow study participants

for one additional year following chemopreventive therapy to examine for "rebound" in the incidence of malaria following our intervention.

In contrast to the HIV-unexposed children, the standard of care among HIV-exposed children is to provide TS prophylaxis for the duration of breastfeeding. TS prophylaxis has been shown to be effective for the prevention of malaria [23-25]. Current recommendations are that HIV-exposed children are retested for HIV approximately 6 weeks after the cessation of breastfeeding and if they remain HIV-uninfected, TS prophylaxis is stopped. It is unknown what effect TS prophylaxis during breastfeeding has on the development of naturally acquired antimalarial immunity among HIV-exposed children. It may be that HIV-exposed children develop immunity during the time they are taking TS prophylaxis and therefore would be expected to have less benefit from extended chemoprevention relative to HIV-unexposed children. Alternatively the use of TS prophylaxis may prevent the development of immunity and therefore place children at particular risk for malaria following cessation of TS, a scenario where extended chemoprevention may be of particular benefit. In this study, HIV-exposed children will begin the intervention when they have completed breastfeeding and have been confirmed to remain HIV-uninfected. The intervention will continue until study participants reach 24 months of age and then the study participants will be followed for on additional year to examine for rebound in the incidence of malaria (similar to the HIV-unexposed children).

It is anticipated that the results of this study will provide valuable comparative data on the effect of different chemopreventive strategies on malaria incidence in two distinct patient populations at high risk for malaria. In addition it is anticipated the results of this study will provide insight into the development of naturally acquired antimalarial immunity in the setting of chemopreventive therapy that will differ in terms of the drug regimens, the age at which the intervention is started, and the HIV status of the mothers.

2. STUDY OBJECTIVES

The study objectives outlined below will be tested in two distinct study populations: 1) HIV-unexposed children (HIV-uninfected children born to HIV-uninfected mothers), and 2) HIV-exposed children (HIV-uninfected children born to HIV-infected mothers who remain HIV-uninfected after cessation of breastfeeding). HIV-unexposed children will begin the intervention when they reach 6 months of age. HIV-exposed children will begin the intervention when they have completed breastfeeding and have been confirmed to remain HIV-uninfected.

2.1. Objective 1

To compare the incidence of malaria among infants and children enrolled at 4-5 months of age and randomized to receive no chemoprevention, daily TS, monthly SP, or monthly DP until they reach the age of 24 months. The primary tools currently available for malaria control in African children include the use of insecticide treated bednets (ITNs) and prompt effective treatment of symptomatic disease. However, there are limitations with these interventions and in highly endemic areas young children may still suffer frequent episodes of malaria despite their use. A strategy that has received renewed attention for African children is the use of antimalarial drugs to prevent

malaria (chemoprevention). Although data exist supporting the use of chemoprevention in African children, this strategy has not been adopted into policy and further research is needed on the optimal drugs, dosing strategies, and duration of chemoprevention.

We will test the hypotheses that infants and children who receive chemopreventive therapy will have a lower incidence of malaria compared to those given no chemopreventive therapy, and that the optimal chemopreventive measure will be monthly therapy with DP. This hypothesis will be tested in two distinct patient populations: 1) HIV-unexposed children who have not previously received any chemopreventive therapy, and 2) HIV-exposed children previously treated with TS prophylaxis for the duration of breastfeeding. Secondary outcomes will include comparisons of the incidence of complicated malaria, hospitalizations, diarrheal illnesses and respiratory tract infections; prevalence of anemia, asymptomatic parasitemia, and gametocytemia; and response to antimalarial therapy.

2.2. Objective 2

To compare the incidence of adverse events among infants and children enrolled at 4-5 months of age and randomized to receive no chemoprevention, daily TS, monthly SP, or monthly DP until they reach the age of 24 months. For a chemoprevention strategy to be widely implemented, it must be safe and well tolerated. Additional data is needed to balance the protective efficacy of various chemoprevention strategies against their potential for causing drug associated adverse events.

We will test the hypothesis that the incidence of adverse events related to study drugs will be lower in infants and children who receive monthly DP compared to those receiving monthly SP and daily TS. Secondary outcomes will include comparisons of the incidence of serious adverse events related to study drugs and the risk of discontinuation of study drugs.

2.3. Objective 3

To compare the incidence of malaria among children for 1 year following intervention with no chemoprevention, daily TS, monthly SP, or monthly DP. A concern with chemopreventive therapy is a potential increased risk of malaria after therapy is discontinued due to decreased antimalarial immunity, and this risk may be greatest with continuous chemoprophylaxis.

We will test the hypothesis that compared to children who received no chemopreventive therapy, those previously given chemoprophylaxis (daily TS), but not those given IPT (monthly SP or DP), will have a higher incidence of malaria. Secondary outcomes will include comparisons of the incidence of complicated malaria, hospitalizations, diarrheal illnesses and respiratory tract infections; prevalence of anemia, asymptomatic parasitemia, and gametocytemia; and response to antimalarial therapy.

3. STUDY DESIGN

Convenience sampling will be used to enroll a cohort of 600 HIV-uninfected infants between the ages of 4-5 months of age according to the following strata based on the mother's HIV status: 1) 200 HIV-exposed infants born to HIV-infected mothers, and 2) 400 HIV-unexposed infants born to HIV-uninfected mothers. Potential study participants

will be identified from the Tororo District Hospital Antenatal Clinic and surrounding clinics providing routine pediatric care. Potential study participants less than 6 months of age and their parents/guardians will be referred to our study clinic for screening. Eligible children will be enrolled when they reach 4-5 months of age and followed until the age of 36 months for all their routine medical care at our designated study clinic. All mother-child pairs will receive 2 long lasting ITNs at enrollment and, as available, a basic care package including a safe water vessel, multivitamins and condoms. HIV-unexposed children will be randomized to one of four chemoprevention arms when they reach 6 months of age. All HIV-exposed children born to HIV-infected mothers will be given TS prophylaxis and mothers will be encouraged to introduce food at 6 months of life and continue breastfeeding until 1 year of life, in accordance with Ugandan Ministry of Health (MOH) guidelines. HIV-exposed children will retested for HIV approximately every 60 days during breastfeeding and 6 weeks following cessation of breastfeeding. HIV-exposed children who remain HIV-uninfected following cessation of breastfeeding will be randomized to one of four chemoprevention arms. HIV-exposed children who test positive for HIV during the course of the study (those who seroconvert during breastfeeding) will be excluded from the study and referred for appropriate care.

During the follow-up period, all patients presenting to the clinic with a new episode of fever will undergo standard evaluation (history, physical examination and Giemsa-stained blood smear) for the diagnosis of malaria. Children diagnosed with uncomplicated malaria will be treated with AL and children diagnosed with complicated malaria will be treated with quinine in accordance with national guidelines. Response to antimalarial therapy will be assessed using standardized guidelines. All AL treatment failures occurring within 14 days of diagnosis will be treated with quinine in accordance with national guidelines. In the event that a patient fails quinine therapy, therapy will be repeated with quinine plus clindamycin. Patients with complicated malaria who have contraindications to giving quinine will be treated with parenteral artesunate. All episodes diagnosed more than 14 days after a previous episode will be considered new episodes for treatment purposes. After two years of age, patients with uncomplicated malaria will have follow up visits on the days Antimalarial drugs are administered only (Day 0, 1, and 2). Routine assessments will be performed in the study clinic approximately every 30 days. Routine assessments will include review of study protocol with parents/guardians of study participants, assessment for any outside medical care, assessment for adherence to assigned chemopreventive therapy, a focused history and physical examination and routine blood smears for the detection of asymptomatic parasitemia. Routine phlebotomy will be performed approximately every 120 days for all study participants for CBC, glucose and ALT measurements. A summary of the study profile is presented in Figure 4.

Figure 4. Study profile

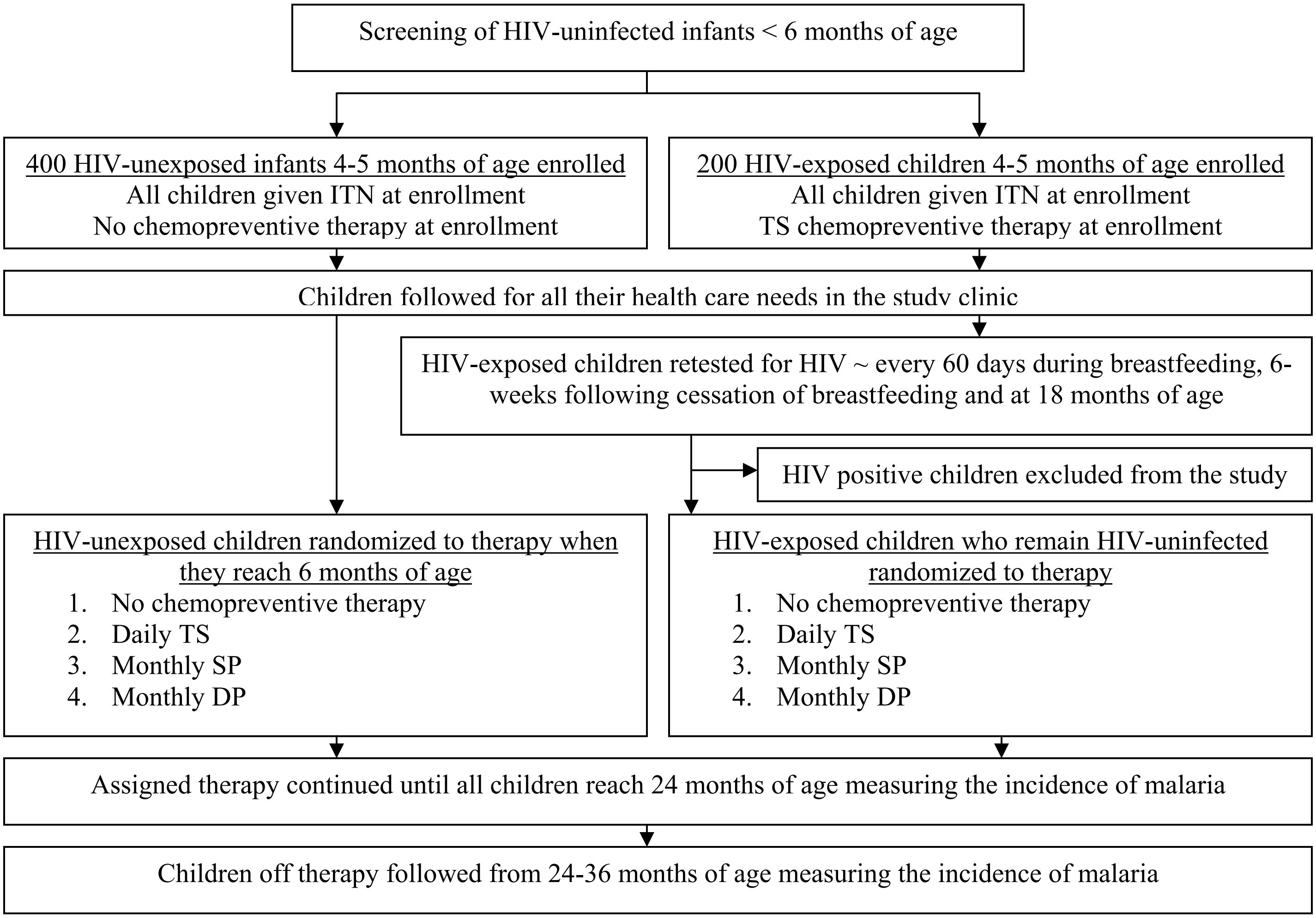

4. SELECTION AND ENROLLMENT OF SUBJECTS

4.1. Inclusion Criteria

1. Age 4 -5 months
2. Confirmed HIV status of biological mother
3. Negative HIV DNA PCR test at time of enrollment for infants born to HIV-infected mothers
4. Infants born to HIV-infected mothers must be breastfeeding
5. Residency within 30km of the study clinic
6. Agreement to come to the study clinic for any febrile episode or other illness and avoid medications given outside the study protocol
7. Provision of informed consent by parent/guardian

4.2. Exclusion Criteria

1. History of allergy or sensitivity to TS, SP, or DP
2. Active medical problem requiring in-patient evaluation at the time of screening
3. Intention of moving more that 30km from the study clinic during the follow-up period
4. Chronic medical condition (i.e. malignancy) requiring frequent medical attention
5. Living in the same household as a previously enrolled study participant
6. QTc interval > 450 msec
7. Other clinically significant ECG abnormalities such as arrhythmia, ischemia, or evidence of heart failure
8. Family history of Long QT syndrome
9. Current use of drugs that prolong the QT interval

4.3. Initial Screening

Potential study participants less than 6 months of age and their parents/guardians will be identified through the TDH Antenatal Clinic and other clinics within the Tororo District including the TASO clinic. Parents/guardians will be approached about participating in the study and will be provided an information sheet about the requirements of the study (see Appendix A). If the parents or guardians are initially agreeable to screening for participation in the study the child and parents will either be escorted to the study clinic or made an appointment to return at a later date. During the screening process, study physicians will assess for initial eligibility criteria through conversation with the parent/guardian (including age of the child, willingness to come to the study clinic for any febrile episode and avoid medications given outside the study protocol, residence within a 30 km radius of the study clinic and no intention to move outside of the study area, no history of allergy or sensitivity to TS or SP or DP, absence of severe illness requiring inpatient evaluation or chronic medical attention requiring frequent medical attention, lack of family history of Long QT syndrome, lack of current use of drugs that prolong the QT interval (see Appendix I), and not living in the same household as a previously enrolled study participant). If the HIV status of the biological mother is not documented and/or infants born to HIV-infected mothers are not confirmed to be HIV-uninfected by DNA PCR testing, appropriate referrals for HIV testing will be made to the TDH antenatal clinic which provides these routine services. In addition, infants born to HIV-infected mothers must be breastfeeding to pass initial screening. Study participants who pass initial screening will be given an appointment to the study clinic when the child is 4-5 months of age for assessment of final study eligibility. Children who are 4-5 months of age and fulfill all eligibility criteria (including confirmed HIV status of biological mother and child) on the day of initial screening may proceed to enrollment on the same day.

4.4. Study Enrollment Procedures and Baseline Evaluation

On the day of enrollment all eligibility criteria will be confirmed. Study physicians will conduct the informed consent discussion in the study clinic with the subjects' parent(s) or guardian(s). Informed consent will be conducted in the appropriate language and a translator will be used if necessary. The study will be described and consent obtained in one of 5 languages (Jopadhola, Teso, Swahili, Luganda, or English). The consent forms will be translated into each language and back-translated into English to check for any loss or change of meaning. Following the informed consent discussion, parents or guardians will be asked by the study physicians to sign a written consent form approved by the UCSF Committee for Human Research (UCSF CHR), Makerere University School of Medicine - Research and Ethical Committee (SOM-REC), and the Uganda National Council for Science and Technology (UNCST) for their child to participate in the research study (Appendix B) and a second approved consent form for the future use of biological specimens obtained during the course of the study (Appendix C). If the parent or guardian is unable to read or write, their fingerprint will substitute for a signature, and a signature from a witness to the informed consent procedures will be obtained. Following informed consent, an ECG will be performed to evaluate the QTc interval and assess for other clinically significant ECG abnormalities at baseline, including arrhythmia, ischemia, or evidence of heart failure. A QTc interval of > 450 msec will be confirmed by repeat ECG. Potential study participants with abnormal ECGs will be excluded from enrollment.

Children who fulfill eligibility criteria and whose parents/guardians provide informed consent will be enrolled on the day informed consent is obtained and assigned a study number in ascending order. On the day of enrollment, children will undergo a history and physical examination, including measurement of blood pressure, pulse, temperature, height, and weight. Children will have blood collected by venipuncture (5-10 cc's) for thick blood smear, filter paper sample (for future molecular studies), routine baseline laboratory testing and storage (see section 6.12). Routine baseline laboratory testing will consist of a CBC, glucose and ALT measurement. Children who have history of fever in the previous 24 hours or a temperature $\geq$ 38.0ºC (tympanic) will have their thick blood smear read urgently in the study clinic. Study patients with history of fever in the previous 24 hours or a temperature $\geq$ 38.0ºC (tympanic) and a positive blood smear will be diagnosed with malaria and treated as described in sections 6.4 and 6.5. All mother-child pairs will receive 2 long lasting ITNs at enrollment and, as available, a basic care package including a safe water vessel and condoms. All HIV-exposed children born to HIV-infected mothers will be given daily TS prophylaxis and mothers will be counseled to exclusively breastfeed until their child is 6 months old and encouraged to introduce food at 6 months of life and continue breastfeeding until 1 year of life, in accordance with Ugandan MOH guidelines.

5. STUDY TREATMENT

5.1. Treatment Group Assignments

Study participants will be randomized to one of the following four chemoprevention groups using a 1:1:1:1 scheme: 1) no chemoprevention, 2) daily TS, 3) monthly SP, and 4) monthly DP. Study participants born to HIV-uninfected mothers (HIV-unexposed

children) will be randomized to begin therapy when they reach 6 months of age. Study participants born to HIV-infected mothers (HIV-exposed children) will be randomized to begin therapy when they have been retested for HIV and confirmed to be HIV-uninfected, approximately 8 weeks following the cessation of breastfeeding (approximately 6 weeks duration from the time breastfeeding is stopped until HIV-testing in performed plus approximately 2 weeks to allow for the results of the HIV test to be obtained and bring the child back to the clinic for randomization).

Treatment group assignment will be made according to 2 randomization lists stratified by the mother's HIV status. Randomization lists will be computer generated by a member of the project who will not be directly involved in the conduct of the study. The randomization lists will include consecutive treatment numbers with corresponding random treatment assignments. Randomized codes will correspond to the 4 treatment groups using permuted variable sized blocks of 4, 8 and 12. Sealed copies of the original randomization lists and documentation of the procedure used to generate the lists will be stored in the project administrative offices in San Francisco and Kampala. Prior to the onset of the study, two sets of sequentially numbered, opaque, sealed envelopes will be prepared. Each envelope will be marked on the outside with the treatment group stratum (HIV-unexposed or HIV-exposed) and treatment allocation number. The inside of the envelope will contain a piece of paper with the treatment allocation number and treatment group assignment along with a piece of carbon paper.

5.2. Treatment Allocation

HIV-unexposed study participants will be seen in the clinic when they reach 6 months of age for study treatment allocation. HIV-exposed children will be seen in the clinic when the results of their 6 weeks post-breastfeeding HIV-testing are available for treatment allocation. Study participants will be referred to a study nurse responsible for the allocation of study drug treatment. The study nurse will assign intervention groups as follows:

1. Select next available envelope
2. Note the treatment group stratum and treatment number on the outside of the envelope
3. Write date, time, and study number on the outside of the envelope
4. Open envelope
5. Remove form containing code for treatment group and date, time, and study number (transferred to form via carbon paper inside of envelope)
6. Store form in file box in study clinic
7. Record onto the treatment allocation master list the study number, enrollment date, treatment assignment code, and study medications to be given.
8. Record the assigned treatment in the patient's file.

5.3. Study Drug Dosing and Formulations

Study participants randomized to chemoprevention with TS will receive once daily dosing according to weight based guidelines. Study participants randomized to chemoprevention with SP will receive monthly dosing given as a single dose according to

weight based guidelines. Study participants randomized to chemoprevention with DP will receive monthly dosing given once a day for 3 consecutive days according to weight based guidelines. Details of the drug formulation and dosing regimens of study drugs are included in Table 5 and Appendix D.

Table 5. Drug formulation and labeling

Drug	Formulations	Trade name (Manufacturer)
Trimethoprim-Sulfamethoxazole (TS)	40mgTMP/200mgSMX/5ml suspension<br>20mgTMP/100mgSMX tabs<br>80mgTMP/400mgSMX tabs	Co-trimoxazole<br>(KPI)
Sulfadoxine-Pyrimethamine (SP)	500mg/25mg tabs	Kamsidar<br>(KPI)
Dihydroartemisinin-Piperaquine (DP)	40mg/320mg tabs	Duo-Cotexin<br>(Holley-Cotec)

5.4. Study Drug Administration and Duration

Administration of all study drugs will be open label. Study drugs will be administered at home by the study participant's parents or guardians. Although this is an efficacy trial, it will not be practical to administer the study drugs utilizing a "directly observed therapy" approach, primarily due to the once a day dosing of TS. Parents or guardians will be given pre-packaged study drugs in opaque envelopes with dosing instructions written on the outside. In the case of liquid formulations, the dosing instructions will be written on the outside of the bottle. If a study participant vomits the study drug within 30 minutes of administration, parents or guardians will be instructed to re-administer the drug. If a study participant vomits the study drug a second time within 30 minutes of re-administration, parents or guardians will be instructed to bring the child to the study clinic the following day for evaluation. Study participants will be seen in the clinic when they reach 24 months of age, at which time parents or guardians will be given clear instructions to stop giving chemopreventive therapy. Plans for the monitoring of adverse events to study drugs are detailed in section 8 and anticipated adverse events are detailed in section 11.

5.5. Study Drug Supply, Distribution, and Pharmacy

Study drugs will be kept in a dedicated study pharmacy maintained in the study clinic. Study drugs will be distributed by study nurses at the time study subjects are seen for routine assessments (see section 6.10) done approximately every 30 days. At the time of routine assessments, study nurses will question parents or guardians about the amount of study drug they have at their home. If the parents or guardians have less than a 2 month supply of study drugs, they will be given addition study drugs so that they have an equivalent of a 3 month supply. Detailed records will be kept on the amount of study drug distributed to each study participant.

5.6. Study Drug Accountability

The study pharmacist will maintain complete records of all study drugs received in the study pharmacy. Lot number and number of pills given to each participant at each visit will be recorded. Detailed information on the dosing and schedule of study drugs given at home will be collected from parents/guardians at the time of routine assessments. A registry of all study medication, current product labels, and Certificates of Analysis, provided by suppliers will be maintained within the regulatory binder for the study. The date received, lot number, expiration date, and date used will be recorded for each of the study medications. Monthly inventory of all study medications will be conducted and a record log of investigational medications will be kept at the study clinic.

5.7. Additional Medications

On the day malaria is diagnosed, parents/guardians will receive paracetamol (10mg/kg) with instructions to give to their child at home approximately every 8 hours until the resolution of fever. Patients found to have uncomplicated malaria and a concomitant illness will be treated for both and followed up according to the study protocol. For patients with anemia (Hb < 10 gm/dL), we will follow Integrated Management of Childhood Illness (IMCI) guidelines: anemic children will be given iron sulfate (100 mg daily for 2 weeks) and mebendazole (only children > 1 year of age; 250 mg age 1-2 years; 500 mg > 2 years age; treated no more frequently than every 6 months).

6. SUBJECT MANAGEMENT

6.1. Subject Follow-up

At the time of enrollment, parents/guardians will be instructed to bring their child to the study clinic for all medical care and avoid the use of any outside medications. The study clinic will remain open 7 days a week from 8 a.m. to 5 p.m. Visits will be classified into the following four categories:

1. **New visit**: Any unscheduled visit to the study clinic for a new medical problem.
2. **Malaria follow-up visit**: Any scheduled or unscheduled visit to the study clinic, following the diagnosis of malaria, during the standardized 28-day follow-up period.
3. **Non-malaria follow-up visit**: Any scheduled or unscheduled visit at the study clinic, for a previously diagnosed non-malaria illness.
4. **Routine assessment**: Any assessment done at the study clinic to assure protocol compliance and obtain routine clinical and laboratory data at regularly scheduled intervals.

6.2. New Visits for Medical Problems

At each new visit, subjects will undergo a standardized history and physical including temperature, pulse, and blood pressure measurement. Patients who are febrile (tympanic temperature $\geq$ 38.0˚C) or report history of fever in the past 24 hours will have blood obtained by finger prick for a thick blood smear (in very young children, heel sticks may be substituted for finger pricks). If the thick blood smear is positive, the patient will be diagnosed with malaria. If the thick blood smear is negative, the patient will be managed by study physicians for a non-malarial febrile illness (Section 6.8). If the patient is afebrile and does not report a recent fever, a thick blood smear will not be obtained, except when following routine testing schedules (Section 6.10).

6.3. Diagnosis of Malaria

All episodes of malaria will be classified as uncomplicated or complicated based on the following criteria:

Uncomplicated malaria (all of the following)

1) Fever ($\geq$ 38.0ºC tympanic) or history of fever in the previous 24 hours
2) Positive thick blood smear
3) Absence of complicated malaria

Study participants with asymptomatic parasitemia with parasite density $\geq$ 100,000/ul identified during a routine assessment will also be considered as having an episode of uncomplicated malaria and brought to the clinic and treated accordingly (see section 6.10)

Complicated malaria (any of the following)

1) Evidence of severe malaria (Appendix E) and parasitemia
2) Danger signs present (Appendix E) and parasitemia
3) Parasite density $\geq$ 500,000/ul

6.4. Management of Uncomplicated Malaria

On Day 0 (the day treatment is initiated) of each malarial episode, patients will undergo a standardized clinical history and physical examination and have blood samples obtained by venipuncture for thin blood smear, CBC, serum/plasma for immunology studies, parasite culture, and filter paper collection. Patients with uncomplicated malaria will be treated with standard doses of AL (twice daily for 3 days), in accordance with national guidelines. Each first daily dose of antimalarial therapy on days 0-2 will be given by a study nurse in clinic and directly observed. Any patient who vomits the medication within 30 minutes of administration will be retreated with a second dose. Any patient who vomits repeatedly (> 3 times) will be recorded as having complicated malaria and referred to Tororo District Hospital for standard treatment with parenteral quinine (see section 6.5). All antimalarial therapy given in the study clinic will be recorded in a

treatment administration book. Patients will be given their second daily dose with instructions to take at home.

For each episode of malaria, patients will be evaluated clinically on Days 1, 2, 3, 7, 14, 21, 28, and on any unscheduled day in which the child is brought to the clinic by the parent/guardian (Table 6). After 2 years of age, patients will be evaluated clinically on Days 1 and 2 only. Patients who do not return for a scheduled visit will be visited at home and instructed to come to the clinic as soon as possible or if necessary, transported to the study clinic. Blood will be obtained by finger prick on Days 1, 2, 3, 7, 14, and 21 for thick blood smears (for parasite density and gametocytes) and filter paper collection and on Day 7 only, for hemacue measurement of hemoglobin levels. Blood will be obtained by finger prick on any unscheduled day, only when a fever is documented or reported in the previous 24 hours, for thick blood smears (for parasite density and gametocytes) and filter paper collection. On Day 28 (or day 29/30 if the patient is not seen on day 28) blood will be collected by venipuncture for thick blood smear, filter paper collection, and CBC.

6.5. Management of Complicated Malaria

Any patient who is diagnosed with severe malaria or danger signs (Appendix E), either on a new visit or during malaria follow-up, will be referred to TDH for consideration for admission and treatment with parenteral quinine. Patients not admitted to Tororo District Hospital will receive standard doses of oral quinine through the study clinic and continue follow-up at the study clinic using the same schedule as outlined in Table 6. Patients with complicated malaria who have contraindications to giving quinine will be treated with parenteral artesunate. Hospitalized patients will be seen daily by study personnel and will undergo scheduled study follow-up procedures. After hospital discharge, follow-up will involve the same schedule as that described for those with uncomplicated malaria.

Table 6. Follow-up schedule for malaria treatment episodes up to two years of age

	Day 0	Day 1	Day 2	Day 3	Day 7	Day 14	Day 21	Day 28	Unscheduled
History	X	X	X	X	X	X	X	X	X
Physical exam	X	X	X	X	X	X	X	X	X
Temperature	X	X	X	X	X	X	X	X	X
Case record form	X	X	X	X	X	X	X	X	X
Thick blood smear	X	X‡	X	X	X	X	X	X	X†
Thin blood smear	X								
Filter paper sample	X	X‡	X	X	X	X	X	X	X†
Hgb (hemacue)					X				
CBC	X							X	
Parasite culture	X								
Serum/plasma for immunology studies	X								

‡ Done only in cases where complicated malaria suspected

† Thick smear and filter paper sample obtained only if temperature elevated (≥ 38.0˚C tympanic) or history of fever in previous 24 hours

Table 6a. Follow-up schedule for malaria treatment episodes after two years of age

	Day 0	Day 1	Day 2	Unscheduled
History	X	X	X	X
Physical exam	X	X	X	X
Temperature	X	X	X	X
Case record form	X	X	X	X
Thick blood smear	X	X‡	X	X†
Thin blood smear	X			
Filter paper sample	X	X‡	X	X†
Hgb (hemacue)	X			
Parasite culture	X*			

‡ Done only in cases where complicated malaria suspected
† Thick smear and filter paper sample obtained only if temperature elevated (≥ 38.0˚C tympanic) or history of fever in previous 24 hours
* In a subset of patients

6.6. Malaria Treatment Outcome Classification System

28-day treatment outcomes will be measured using the standard WHO classification system (Appendix F). Patients who meet any of the following criteria will not be assigned a treatment outcome but will continue to be followed in the cohort study unless consent is withdrawn:

1) use of antimalarials outside of the study protocol
2) lost to follow-up (unable to locate patient on Days 1-2, or unable to locate patient within 24 hours of Day 3, or unable to locate patient within 48 hours of Days 7-28)
3) withdrawal of informed consent

6.7. Management of Clinical Treatment Failures

Patients treated for episodes of uncomplicated malaria that are classified as early or late clinical failures according to the standard WHO outcome classification system (Appendix 15.6) within 14 days of initiation of treatment will be treated with quinine, beginning a new 28-day follow-up period. Patients treated for complicated malaria with quinine that are classified as early or late clinical failures within 14 days of treatment will be treated with quinine plus clindamycin, beginning a new 28-day follow-up period. Late clinical failures occurring on day 15-28 will be treated as new episodes of malaria (AL for uncomplicated malaria, quinine for complicated malaria). Adequate clinical and parasitological responses or late parasitological failures will be given no additional antimalarial treatment. Patients with no treatment outcomes will be managed according to the criteria below.

1) use of antimalarials outside of the study protocol – treatment as above if criteria for early or late clinical failure met.
2) lost to follow-up – treatment as above if criteria for early or late clinical failure met.

3) withdrawal of informed consent – no further follow-up within the study but will receive continued standard of care at TDH.

6.8. Management of Non-Malaria Illnesses

Patients who are found to have illnesses other than malaria will receive standard-of-care treatment in the study clinic, according to standardized algorithms, or will be referred to the Tororo District Hospital. We will avoid the routine use of non-study medications with antimalarial activity, including tetracyclines, antifolates (with the exception of assigned chemopreventive regimens), and macrolide antibiotics, when acceptable alternatives are available. We will also avoid the use of medications known to cause prolongation of the QT interval (see Appendix I) in study participants receiving DP. During follow-up for non-malarial illnesses, blood smears will be done at the discretion of the study physician if the subjects are febrile (tympanic temperature $\geq$ 38.0˚C) or report history of fever in the past 24 hours. If the blood smear is positive, the patient will be diagnosed with a new episode of malaria and managed per study protocol. If a patient comes to the study clinic for a non-malarial illness and 30 days have passed since the last blood smear, a routine assessment will be completed in the study clinic (Section 6.10). If a patient is diagnosed with a non-malarial illness at the same time as malaria or during malaria follow-up, treatment of the non-malarial illness will be at the discretion of the physician, but this will have no impact on the management of malaria.

6.9. After Hours Visits

Parents/guardians will be encouraged to bring their child to the TDH pediatric inpatient ward (open 24 hours a day) when urgent care is needed outside of study clinic hours. Parents/guardians of study participants will be instructed to inform hospital personnel of their involvement in the study at the time of registration and to visit the study clinic on the following day. If a patient is diagnosed with uncomplicated malaria they will receive treatment from a hospital supply of AL and the doctors will be instructed to refer patients to our study clinic when it opens at 8 am the following day. If a patient is diagnosed with severe malaria, he/she will receive quinine following standard treatment guidelines. Patients with non-malarial illnesses will be managed at the discretion of the Tororo District Hospital staff. Upon discharge, patients will receive follow-up at the study clinic as outlined above. Study personnel will visit the Tororo District Hospital daily to inquire about visits from study subjects and facilitate follow-up in the study clinic.

6.10. Routine Assessments

Routine assessments will be done in the clinic approximately every 30 days. To ensure the proper timing of routine assessments, study participant will be scheduled to come to the study clinic within a minimum of 30 days following any visit. If a study participant is not seen in the study clinic after a period of approximately 30 days, they will be visited at home and requested to come to the clinic. During routine assessments subjects will be

asked about visits to outside health facilities and the use of any medications outside the study protocol. The study protocol will be reinforced with discussion regarding the need to come to the study clinic promptly upon the onset of any illness and to avoid use of outside medications. Standardized assessment of adherence will also be done for assigned chemopreventive therapy and ITN use. A routine history and physical exam (including blood pressure and pulse) will be performed using a standardized clinical assessment form. If a parent/guardian reports a fever in the last 24 hours or the child has a documented temperature $\geq$ 38.0°C tympanic, the subject will be evaluated for a new visit (see section 6.2). For participants without fever, blood will be collected by finger prick (in very young children, heel sticks may be substituted for finger pricks) for thick smear and filter paper samples to assess for asymptomatic parasitemia. Routine smears will be read within 48 hours, and asymptomatic children with parasitemia $\geq$100,000/µl will be considered to have new episodes of malaria, brought to the study clinic within 24 hours, and treated for malaria. In our experience, asymptomatic patients with a parasite density $\geq$ 100,000/µl have a high risk of developing symptomatic malaria in a short period of time. Asymptomatic patients with a parasite density of < 100,000/µl will not be treated in accordance with standard practices in endemic areas of Africa. Phlebotomy for routine laboratory tests (CBC, Glucose and ALT) to monitor for potential adverse events from study medications and for immunology studies will be performed approximately every 120 days. Every 5th study participant enrolled in the study and randomized to receive monthly DP (~ 40 children) will have an ECG performed at the time of their 30 day routine assessment during which time they are receiving DP. The timing of routine ECG's will be scheduled such that they occur 4-5 hours after the last of the 3 doses of DP received each month. The schedule for routine assessments is summarized in Table 7.

Mothers of HIV-exposed child will be counseled on HIV and infant feeding at the time of monthly routine assessments by trained personnel. The following guidelines will be used in accordance with Ugandan and WHO recommendations (Inter-agency Task Team on Prevention of HIV Infection in Pregnant Women, Mothers, and their Infants, 2007):

- Exclusive breastfeeding is recommended for HIV-infected women for the first six months of life unless replacement feeding is acceptable, feasible, affordable, sustainable and safe for them and their infants before that time.
- When replacement feeding is acceptable, feasible, affordable, sustainable and safe, avoidance of all breastfeeding by HIV-infected women is recommended.
- At six months, if replacement feeding is still not acceptable, feasible, affordable, sustainable and safe, continuation of breastfeeding with additional complementary foods is recommended, while the mother and baby continue to be regularly assessed. All breastfeeding should stop once a nutritionally adequate and safe diet without breast milk can be provided.

HIV-exposed children will retested for HIV approximately every 60 days during breastfeeding , 6 weeks following cessation of breastfeeding, and at 18 months of age, regardless of the age at which they stopped breastfeeding, at the time of routine assessments. HIV-exposed children who test positive for HIV during the course of the study (those who seroconvert during breastfeeding) will be excluded from the study and referred for appropriate care.

When children reach 24 months of age, the patents/guardians of those randomized to study drugs (daily TS, monthly SP, or monthly DP) will be administered a questionnaire on the knowledge and acceptability of the study drugs (Appendix M).

Table 7. Follow-up schedule for routine assessments

Procedure	Baseline	Approximately every 30 days	Approximately every 120 days
Malaria smear / filter paper	X	X	
Routine history/physical	X	X	
Adherence to study medications and ITN use		X	
Repeat HIV DNA PCR testing*			X
CBC/Glucose/ ALT	X		X
Immunology studies	X		X
ECG in subset of participants randomized to receive DP	X	X	
PK Plasma sample in subset of participants randomized to receive DP			X**

* HIV-exposed, breastfeeding study participants only, additionally will be done 6 weeks following cessation of breastfeeding and at 18-months of age regardless of breastfeeding status

** One PK plasma sample will be done at the time of the first 120 day visit after starting chemoprevention therapy.

6.11. Medical Care Outside the Study Clinic

We will provide routine medical care, including medications, in our clinic free of charge, and we will reimburse patients for costs of any transportation to and from our clinic. In addition, we will reimburse the cost of tests and drugs for referrals made by study physicians to other clinics and services as well as after hours visits at Tororo District Hospital. We anticipate reimbursing the cost of most diagnostic tests (including laboratory tests, X-rays, and ultrasounds) and medications resulting from these referrals, using available funds. However, reimbursement of all diagnostic tests and treatment recommended outside the study clinic cannot be guaranteed in all circumstances because neither Makerere University, the University of California, San Francisco, nor the funding agency have a program to cover these costs. Decisions on reimbursement will be made by the study coordinator and the investigators, in conjunction with the funding agency if necessary.

6.12. Criteria for premature subject discontinuation

Enrolled subjects will be withdrawn from the study for the following reasons:

1. Movement out of study area or inability to be located for > 60 consecutive days
2. Withdrawal of informed consent
3. Unable to comply with the study schedule and procedures
4. At the discretion of the investigator
5. Parent/guardian judged by the site investigator to be at significant risk of failing to comply with the provisions of the protocol as to cause harm to

the study participant or seriously interfere with the validity of the study results
6. Request of a primary care provider if he/she thinks the study is no longer in the best interest of the participant
7. HIV-exposed study participants who are infected with HIV during breastfeeding
8. HIV-unexposed study participants who are breastfeeding and the mother has been found to have become HIV-infected
9. Study participant who is not seen in clinic and randomized within 2 weeks of their scheduled randomization date

If a subject is withdrawn for reasons # 1 or 2, we will be unable to perform any additional study procedures. If a subject is withdrawn for reasons # 3-9, plans to obtain appropriate follow-up tests outside of the study will be individualized for each subject depending on the health status of the subject at the time of withdrawal and the willingness of the participant and his or her parent/guardian to proceed with additional testing.

6.13. Diagnostic and Laboratory Testing

6.13.1. Microscopy

Thick and thin blood smears will be stained with 2% Giemsa and read by experienced laboratory technologists who are not involved in direct patient care. Parasite densities will be calculated by counting the number of asexual parasites per 200 leukocytes (or per 500 leukocytes, if the count is <10 asexual parasites/200 leukocytes), assuming a leukocyte count of 8,000/µl. A blood smear will be considered negative when the examination of 100 high power fields does not reveal asexual parasites. Gametocytemia will also be determined from thick smears. Thin smears will be used for parasite species identification. Urgent thick smears will be read in our study clinic for initial diagnosis and to identify treatment failures during follow-up. Routine blood smears will be read within 48 hours. For quality control, all slides will be read by a second microscopist and a third reviewer will settle any discrepant readings.

6.13.2. Clinical Laboratory Studies

At baseline, approximately every 120 days, and on the day malaria is diagnosed, venipuncture blood samples will be collected for clinical laboratory studies, including CBC, Glucose and ALT measurements. Additional venipunctures will be performed, as appropriate, for laboratory testing to evaluate non-malarial medical illnesses at the discretion of study physicians. Turn around time for clinical laboratory studies is expected to be 1-3 days. Results will be made available to study physicians for patient management decision-making.

6.13.3. Molecular, Parasitology, Immunology, and PK Studies

Each time a thick blood smear is obtained; blood will also be collected onto filter paper. Samples will be collected by venipuncture or by finger prick sampling. Blood will be placed onto filter paper in approximately 25 µl aliquots per blood spot (4 blood spots per sample). The samples will be labeled with study numbers and dates, air-dried, and stored in small, sealed sample bags at ambient temperature or 4°C with desiccant. Molecular studies will include the extraction of DNA from filter paper and followed by characterization of parasite genetic polymorphisms using standard molecular procedures including PCR, DNA hybridization, and/or restriction enzyme digestion. For all repeat episodes of malaria, molecular genotyping methods, similar to those described for polymorphism analysis, will be used to distinguish recrudescent from new infections. Additional molecular studies will include analyses of polymorphisms in parasite genes for mutations that may impact on malaria infection and response to antimalarial therapy. Molecular studies will be performed only for research purposes and will have no impact on the clinical management of study patients.

Blood collected by venipuncture on the day malaria is diagnosed will be used in selected subjects for parasite culture. For these studies the skin will be prepped with 3 washes with a betadine or equivalent sterilizing solution, and then approximately 1 ml of blood will be collected in a anticoagulated sterile tube and transferred promptly (generally within 30 minutes) to our molecular laboratory. Parasitology studies will only require phlebotomy required also for laboratory studies at the time of malaria diagnosis. Parasites will be cultured following standard protocols. In brief, erythrocytes will be separated from plasma by centrifugation and removal of the supernatant and buffy coat, and the infected erythrocytes will then be cultured in RPMI medium supplemented with human serum or Albumax serum substitute. Cultured parasites will be evaluated for in vitro drug sensitivity, molecular characteristics, and other features to characterize antimalarial drug resistance and other aspects of malaria. Information from the parasitology studies will have no impact on patient care.

As resources permit, venipuncture blood samples will be collected in selected subjects at baseline, on the day malaria is diagnosed, and approximately every 120 days at the time of routine assessments will be used in selected subjects for immunology and PK studies. Approximately 5ml of blood will be collected and separated into plasma and peripheral blood mononuclear cells (PBMC) using a Ficoll gradient, following standard protocols. Plasma will be stored at -80C for future immunologic and PK studies, which may include measurement of levels of cytokines, antibodies, and other features related to the host immune response. PBMCs will be stored at -80°C or in liquid nitrogen to maintain viability, and will be evaluated using flow cytometry, ELISPOT, and other assays to assess the host immune response. Information from immunology studies will have no impact on patient care. Stored plasma samples and dried blood spots on filter paper will be available for measuring DHA and piperaquine levels in a subset of patients who are randomized to receive DP and selected to undergo routine ECGs or develop malaria. The timing of the first routine phlebotomy following initiation of DP will be made such that it corresponds to the time the ECG is done.

A summary of all scheduled clinical and laboratory evaluations are presented in Appendix G.

6.13.4. Planned tests on banked specimens

In a select number of patients, banked patient specimens (such as DBS and plasma) will be utilized to investigate other microbial non-malarial causes of fever, including bacteria and viruses. We will only utilize excess volumes of specimens that had already been collected and stored for future use (see Appendix E, "INFORMED CONSENT FOR FUTURE USE OF BIOLOGICAL SPECIMENS"). A pan-viral microarray (Virochip) and ultra – deep sequencing will be utlized. The current Virochip platform consists of approximately 16,000 oligonucleotide probes derived from all 400k records in Genbank as of January 2007. Ultra high-throughput deep sequencing using Solexa ™ genetic analyzers will also be performed for some specimens. These studies will be performed only for research purposes and will have no impact on the clinical management of study patients and results will not be returned to the clinicians or the patients.

6.14. Nutritional Visits

PROMOTE-Chemoprevention participant's parent/guardians may be asked to take part in optional activities about food and nutrition. The purpose of these activities is to inform the design and implementation of a nutritional support intervention for PROMOTE participants. We will plan to ask the parents/guardians of all participants a short list of questions about food security and home hygiene that should take less than 20 minutes (Appendix L). In addition, we plan to ask a subset of participant parents/guardians for their consent to participate with in-depth questioning. These interviews will take no more than 2 hours and will occur either in a focus group, one-on-one setting, or both, based on participants' preference and availability. We will collect information about food preferences, beliefs, practices, nutritional challenges, and opinions about potential micronutrient supplements (micronutrient sprinkles, tablets, and syrups). The focus group sessions will be digitally recorded to facilitate later transcription and translation. Participation is entirely voluntary and a separate consent form will be completed for those who chose to participate in a focus group discussion and/or in-depth interview (See Appendix M). The specific number of parent/guardians of participants to be asked to participate in individual interviews and/or the focus groups will depend on resources and participant visit scheduling.

6.15. Household questionnaire

Following enrollment of study participants a household questionnaire will be administered to the primary care giver of the study participant. Appointments will be made with the primary care giver and the questionnaire will be administered at the home of the study participants by study staff. The questionnaire will be administered using a paperless system with tablet computers. Prior to administering the questionnaire verbal consent from the primary care giver will be obtained. A list of all questions and pre-specified responses are included in Appendix O and the verbal consent script in Appendix P. Questions will include characteristics of the household, details of the study participant's sleeping area, characteristics of the primary care giver, and the primary care giver's thoughts about home improvement. The primary purpose of the household

questionnaire will be to identify potential household level risk factors associated with the incidence of malaria measured on the study participant.

7. Management of Toxicities Related to Study Medications

The following section outlines management of toxicities for participants randomized and receiving SP, DP or TS for the purposes of chemoprevention therapy. Participants not yet randomized (including participants taking TS as part of standard treatment for HIV exposure and not for chemoprevention) or randomized to the 'no chemoprevention' arm will be managed on a case by case basis as clinically indicated and at the discretion of the medical officer. Additionally, participants receiving SP, DP or TS will be followed up according to these toxicity management guidelines up to one month following cessation of chemoprevention therapy and thereafter managed on a case by cases basis as clinically indicated and at the discretion of the medical officer.

7.1. Grade 1 or 2 Toxicities

Participants who develop grade 1 or 2 adverse events or toxicity may continue study medications without alteration of dosage. The site physicians will manage the grade 1 or 2 events according to standard practice.

7.2. Grade 3 or 4 Toxicities

Management will be as follows:

- Repeat observation or lab test within 72 hours of observation or of receiving lab results report.
- For grade 3 or non-life threatening grade 4 toxicity, subjects may continue taking study drugs pending clinic visit or repeat laboratory tests. Clinician has the option of immediately stopping the study drugs if subject cannot be examined in clinic, if a repeat laboratory test cannot be performed within 72 hours, or if the clinician determines that the continuation of study drugs is unsafe while awaiting clinic exam or test results. For grade 4 life-threatening toxicity, subjects should hold study drugs pending laboratory confirmation.
- Work-up to exclude other causes.
- For all Grade 3 or 4 toxicities supported by repeat clinical exam or laboratory test results, study drugs will be stopped until toxicity resolves to ≤ Grade 2 unless there is strong evidence that the toxicity is not related to study drugs.
- If toxicity persists at Grade 3 or 4 for more than 14 days or recurs on re-challenge, study drugs will be permanently discontinued.

In the event that study drugs are permanently discontinued, study participants will remain in the study, following our intention-to-treat analysis approach (see section 9.1.3).

7.3 ECG Related Toxicities

A conservative approach will be used for abnormal ECGs (applies only to subset of patients randomized to DP). Management will be as follows:

- Hold DP for any abnormal ECG (grades 1-4) and notify study team to determine course of action.
- Arrange for subject to be examined in clinic for repeat ECG within 72 hours and notify study team of those results.
- For all ECG abnormalities confirm at the time of repeat testing, DP will be permanently discontinued and the DSMB will be notified

8. MONITORING OF ADVERSE EVENTS AND MANAGEMENT

8.1. Monitoring and Reporting of Adverse Events

8.1.1. Definitions

An adverse event is defined as "any untoward medical occurrence in a patient or clinical investigation subject administered a pharmaceutical product that does not necessarily have a causal relationship with this treatment" (ICH Guidelines E2A). An adverse event can further be broadly defined as any untoward deviation from baseline health, which includes:

- Worsening of conditions present at the onset of the study
- Deterioration due to the primary disease
- Intercurrent illness
- Events related or possibly related to concomitant medications

(International Centers for Tropical Disease Research Network Investigator Manual, Monitoring and Reporting Adverse Events, 2003).

8.1.2. Identification of Adverse Events

At each scheduled and unscheduled visit to the clinic, study clinicians will assess patients according to a standardized clinical record form. A severity grading scale, based on toxicity grading scales developed by the NIH Divisions of AIDS and Microbiology and Infectious Diseases Pediatric Toxicity Tables, will be used to grade severity of all symptoms, physical exam findings, and laboratory results (Appendix H). Although, all participants, regardless randomization status or treatment arm, will be assessed using the same standardized clinical record form, only new events occurring in participants randomized and receiving SP, DP or TS for the purposes of chemoprevention therapy will be considered an adverse event.

Data will be captured on the incidence of all adverse events, regardless of severity. For each adverse event identified and graded as severe or life threatening and felt to be

possibly, probably or definitely related to study drugs, an adverse event report form will be completed. In addition, an adverse event form will be completed for all serious adverse events and unexpected events, regardless of severity. An adverse event report form will not be completed for events classified as mild or moderate (unless they are serious or unexpected), as mild and moderate symptoms are common and difficult to distinguish from signs and symptoms due to malaria and other common illnesses. The following information will be recorded for all adverse experiences that are reported:

1) Description of event
2) Date of event onset
3) Date event reported
4) Maximum severity of the event
5) Maximum suspected relationship of the event to study drugs
6) Whether the event is a serious adverse event
7) Initials of the person reporting the event
8) Outcome
9) Date event resolved

Additionally, participants receiving SP, DP or TS for chemoprevention will be continue adverse event follow up for one month only, following cessation of chemoprevention therapy.

8.1.3. Reporting of Adverse Events

Guidelines for reporting of adverse events provided by NICHD, UCSF Committee for Human Research (CHR), and the Food and Drug Administration (FDA) in the U.S. and the Makerere University IRB, Ugandan National Council for Science and Technology (UNCST) and National Drug Authority (NDA) in Uganda will be followed as summarized in Table 8 below.

Of note, as described above, adverse events will reported only from participants randomized and receiving SP, DP or TS for the purposes of chemoprevention therapy and up to one month following the cessation of chemoprevention therapy. Participants not yet randomized (including participants taking TS as part of standard treatment for HIV exposure and not for chemoprevention) or randomized to ‘no chemoprevention’ arm or greater than one month following the cessation of chemoprevention therapy will not require adverse event reporting.

Table 8. Guidelines for reporting adverse events

Institution	Type of Adverse Events	When to Report
**NICHD**	• Definitely, Probably, or Possibly related **AND** Serious or Unexpected	• Within 3 working days of awareness
**UCSF-CHR**	• Definitely, Probably, or Possibly related **AND** Serious or Unexpected	• Within 10-working days of awareness
**UNCST**	• All Serious and Unexpected events irrespective of relationship;	• Within 7-calendar days of awareness<br>• All other reportable events within 15-calendar days of awareness
**MU-SOMREC**	• All Serious and Unexpected events irrespective of relationship;	• Fatal or life-threatening events within 3 working days of awareness<br>• All other SAEs within 10 working days of awareness
**NDA**	• All Serious and Unexpected events irrespective of relationship	• Within 7 calendar days of awareness
**FDA**	• Definitely, Probably or Possibly related **AND BOTH** Serious* AND Unexpected±	• For fatal or life-threatening events, by telephone or fax within 7 calendar days of first awareness<br>• All other reportable events within 15 calendar days of first awareness

Serious Adverse Event (SAE) is any AE that results in any of the following outcomes:

- Death,
- Life-threatening adverse experience.
- Inpatient hospitalization or prolongation of existing hospitalization,
- Persistent or significant disability/incapacity,
- Congenital anomaly/birth defect, or cancer, or
- Any other experience that suggests a significant hazard, contraindication, side effect or precaution that may require medical or surgical intervention to prevent one of the outcomes listed above,
- Event occurring in a gene therapy study
- Event that changes the risk/benefit ratio of the study.

Unexpected Adverse Event An adverse event is defined as being unexpected if the event exceeds the nature, severity, or frequency described in the protocol, consent form and investigator brochure (when applicable). An unexpected AE also includes any AE that meets any of the following criteria:

- Results in subject withdrawal from study participation,
- Due to an overdose of study medication, or
- Due to a deviation from the study protocol

9. STATISTICAL CONSIDERATIONS

9.1. Hypothesis 1

We will test the hypotheses that infants and children who receive chemopreventive therapy will have a lower incidence of malaria compared to those given no chemopreventive therapy, and that the optimal chemopreventive measure will be monthly therapy with DP. Secondary outcomes will include comparisons of the incidence of complicated malaria, hospitalizations, diarrheal illnesses and respiratory tract infections; prevalence of anemia, asymptomatic parasitemia, and gametocytemia; and response to antimalarial therapy.

9.1.1. Primary Outcome

The primary endpoint will be the incidence of malaria defined as the number of treatments for new episodes of malaria per time at risk. Incident cases of malaria will include all first-line treatments given for uncomplicated and complicated malaria. Second-line treatments given for clinical treatment failures occurring within 14 days will not be considered incident events. Time at risk will begin when study participants are randomized to chemopreventive therapy and will end when study participants reach 24 months of age or early study termination.

9.1.2. Secondary Outcomes

Secondary outcomes are summarized in Table 9 below.

Table 9. Secondary outcomes

Secondary outcome	Definition
Incidence of complicated malaria	Any treatment for malaria meeting WHO criteria for severe malaria, danger signs present, or parasite density ≥ 500,000/ul
Incidence of hospital admissions	Admission to the Tororo District Hospital pediatric ward for any cause
Incidence of diarrheal illnesses	Defined by our diagnostic coding system
Incidence of respiratory tract infections	Defined by our diagnostic coding system
Prevalence of anemia	Proportion of hemoglobin measurements < 10 g/dL. Anemia will also be defined according to severity: mild (8.5-10), moderate (7.5-8.4) and severe (< 7.5).
Prevalence of asymptomatic parasitemia	Proportion of routine blood smears done in asymptomatic study participants positive for asexual parasites.
Prevalence of gametocytemia	Proportion of routine blood smears done in asymptomatic study participants positive for gametocytes.
Response to antimalarial therapy	Risk of 28-day treatment failure following therapy with AL for episodes of uncomplicated malaria

9.1.3. Analyses

An intention-to-treat approach to all analyses will be used, including all study participants randomized to therapy and including all follow-up time until the study participants reach 24 months of age or early study termination regardless of whether the intervention was stopped to due an adverse event. All analyses will be stratified into the following 2 groups according to the mother's HIV status: 1) HIV-unexposed children born to HIV-uninfected mothers and, 2) HIV-exposed children born to HIV-infected mothers.

Primary analysis. Statistical modeling and comparison of the number of episodes of malaria must take into account each participant's follow-up time, because more time at risk will produce more episodes regardless of which chemopreventive strategy is used. Estimating how the treatment arms differ in terms of malaria episodes per person-year of follow-up is therefore a useful summary. We will accomplish this by using Poisson regression, the standard approach for count data, to model the number of malaria episodes. These models will include the logarithm of the follow-up time as an offset (an offset is similar to a predictor variable, but its coefficient is set to 1.0 instead of being estimated). Because the outcome variable is implicitly logarithmically transformed in Poisson regression, this will have the net effect of modeling log(treatments/follow-up), as desired. We will translate the fitted coefficients and their confidence bounds into percentage effects with the formula 100*[exp(coefficient)-1]. This approach is closely related to exponential survival models for analyzing events per follow-up time, but is better able to adjust for violated assumptions. Simpler methods for rates would require assuming that times between malaria episodes are independent and identical exponential distributions. Testing for overdispersion in the Poisson regression can detect violations of these assumptions, and variances can be adjusted accordingly to produce valid p-values and confidence interval using a negative binomial regression model.

Secondary analyses. Comparisons of other incidence measures (complicated malaria, hospitalizations, diarrheal illnesses, and respiratory tract infections) will use the same statistically modeling approach as described above. Binary outcome measures (anemia, asymptomatic parasitemia, gametocytemia, and treatment failure following antimalarial therapy) will be analyzed using repeated measures logistic regression to model the influence of treatment arm on the probability of the outcome of interest being observed. Because outcomes occurring in the same person may not be independent we will use the Statistical Analysis System's NLMIXED procedure to include a random person effect in the logistic regression models. Pair-wise differences in treatment arms will be estimated and tested, without formal adjustment for multiple comparisons. Instead, interpretation will be cautious and not based solely on whether $p<0.05$ is achieved for any pair-wise comparison; biologic plausibility and magnitudes of estimated effects will also be considered. In addition, an omnibus p-value for any treatment difference will be calculated by a likelihood ratio test.

9.1.4. Sample Size and Accrual

Sample size calculations are based on testing the hypothesis that the incidence of malaria in the monthly SP or DP arms will be significantly lower compared to the daily TS arm

during the period when the intervention will be given. Based on preliminary data (see Table 4), we estimate the incidence of malaria in the daily TS arm will be 1.85 episodes per person year. To approximate the statistical power that can be expected for the analyses of the primary outcome described above, we employ normal approximations for the outcome defined as log[(1+treatments)/follow-up], where we have added 1 to the treatment count in order to allow taking the log. Our preliminary data show that this outcome measure is approximately normally distributed with a mean value of 1.26 and a standard deviation of 0.40 for the HIV-unexposed children (where we expect approximately 10% loss of potential follow-up time) and a standard deviation of 0.50 for the HIV-exposed children (where we expect approximately 30% loss of potential follow-up time given the later age at the time of randomization). For the HIV-unexposed children, we will need to enroll 100 children in each arm (total = 400) to have 80% power to detect a reduction in the incidence of malaria in either the monthly SP or DP arms (two-sided significance level = 0.05) compared to the daily TS arm of 32%. For the HIV-exposed children, we will only be able to enroll 50 children in each arm (total = 200) due to logistical constraints. Given this sample size we will have 80% power to detect a reduction in the incidence of malaria in either the monthly SP or DP arms (two-sided significance level = 0.05) compared to the daily TS arm of 48% or greater.

9.2. Hypothesis 2

We will test the hypothesis that the incidence of adverse events related to study drugs will be lower in infants and children who receive monthly DP compared to those receiving monthly SP and daily TS. Secondary outcomes will include comparisons of the incidence of serious adverse events related to study drugs and the risk of discontinuation of study drugs.

9.2.1. Primary Outcome

Our primary outcome will be the incidence of any adverse events of severity grade 2 or higher that are possibly, probably, or definitely related to study drugs. We have chosen our primary outcome to only include adverse events with severity grade $\geq$ 2 because previous experience has shown us that adverse events of grade 1 (mild) are very common and generally of minimal clinical significance. We will also stratify our outcomes according to specific adverse events (i.e. vomiting, diarrhea, rash, etc.). For hypothesis 2 we will consider the time at risk as the duration from the day of randomization (when the intervention is begun) to when the study participant reaches 24 months of age (when the intervention will be stopped) or when early study termination takes place.

9.2.2. Secondary Outcomes

Secondary outcomes will include the incidence of specific adverse events of grade 1 severity (a measure of tolerability), incidence of serious adverse events, and the risk of discontinuation of study drugs.

9.2.3. Analyses

An intention-to-treat approach to all analyses will be used, including all study participants randomized to therapy and including all follow-up time until the study participants reach 24 months of age or early study termination regardless of whether the intervention was stopped to due an adverse event. All analyses will be stratified into the following 2 groups according to the mother's HIV status: 1) HIV-unexposed children born to HIV-uninfected mothers and, 2) HIV-exposed children born to HIV-infected mothers.

Primary and secondary analyses. For the comparison of the incidence of adverse events between the 3 chemopreventive treatment arms we will use the same statistical modeling approach as outlined in the primary analysis of section 9.1.3. For all adverse event outcomes we will perform analyses aggregated for any type of adverse event as well as analyses for specific adverse events (i.e. vomiting). We will also compare the risk of discontinuation of study drugs over the course of the study. Risk of discontinuation of study drug will be estimated using survival analysis (Kaplan-Meier product-limit formula) to allow for censoring of patients who do not complete the full observation period. Hypothesis testing across treatment groups will be performed by comparing survival curves using the non-parametric log-rank test.

9.2.4. Sample Size and Accrual

We will enroll 100 patients per treatment arm stratified by mothers' HIV status based on the sample size calculations presented in section 9.1.4. It is difficult to predict the incidence of adverse events. In Table 10 we present a range of incidence values and the relative difference we will be able to detect given our sample size and a power of 80% (significant level = 0.05).

Table 10. Relative differences in incidence of adverse events detectable

Incidence of adverse evens in baseline treatment group (n=100)	Relative standard deviation of log[(1+number of adverse events)/follow-up]		
	¼	½	1
1.0 per person year	5%	10%	21%
3.0 per person year	10%	21%	47%
5.0 per person year	13%	29%	65%

9.3. Hypothesis 3

We will test the hypothesis that compared to children who received no chemopreventive therapy, those previously given chemoprophylaxis (daily TS), but not those given IPT (monthly SP or DP), will have a higher incidence of malaria over the 1 year following our intervention (24-36 months of age). Secondary outcomes will include comparisons of the incidence of complicated malaria, hospitalizations, diarrheal illnesses and respiratory tract infections; prevalence of anemia, asymptomatic parasitemia, and gametocytemia; and response to antimalarial therapy.

9.3.1. Primary Outcome

Primary outcomes with be the same as those described in section 9.1.1 with the exception that the time at risk will begin when study participants reach 24 months of age (when the intervention will end) and will end when study participants reach 36 months of age or when early study termination occurs.

9.3.2. Secondary Outcomes

Secondary outcomes with be the same as those described in section 9.1.2 with the exception that the time at risk will begin when study participants reach 24 months of age (when the intervention will end) and will end when study participants reach 36 months of age or when early study termination occurs.

9.3.3. Analyses

The analytical approach will be the same as that described in section 9.1.3.

9.3.4. Sample Size and Accrual

For specific aim 3 we will test the hypothesis that children 24-36 months of age previously given daily TS will have a higher incidence of malaria compared to those who previously received no chemopreventive therapy. Assuming that we will have 85 children in each treatment arm stratified by the mother's HIV status (10% lost to follow-up per year) and an incidence of malaria of 3.90 episodes per person year (based on preliminary data presented in Table 4) in those who previously received no chemopreventive therapy, we will have 80% power (significance level = 0.05) to detect a 49% or greater increase in the incidence of malaria in those previously given daily TS. We will also test the hypothesis that children 24-36 months of age previously given monthly SP or DP will have a similar incidence of malaria (non-inferiority) compared to those who previously received no chemopreventive therapy. Given the same assumptions above, we will have 80% power (significance level = 0.05) to conclude that the incidence of malaria is similar for those previously given monthly SP or DP compared to those previously given no chemopreventive therapy assuming that the difference in malaria incidence does not differ by more than 42%.

Sample size calculations were repeated in June 2011 for the HIV-exposed strata because it was deemed no longer possible to recruit 400 children due time constraints and limitations in resources available. The plan is now to enroll 50 children in each arm for a total of 200 HIV-exposed children. Assuming that we will have 42 children in each treatment arm among the HIV-exposed strata and an incidence of malaria of 3.90 episodes per person year (based on preliminary data presented in Table 4) in those who previously received no chemopreventive therapy, we will have 80% power (significance level = 0.05) to detect a 74% or greater increase in the incidence of malaria in those previously given daily TS.

9.4. Data and Safety Monitoring Plan

9.4.1. Data and Safety Monitoring Board

Pursuant to the NICHD policy for Data and Safety Monitoring: (http://www.nichd.nih.gov/funding/polices/datasafety.cfm?redorforprint=1) a Data Safety and Monitoring Board (DSMB) will be convened to provide oversight of the clinical trial. The role of the DSMB will be to review implementation and progress of the trial and to review the accumulating data from the study to detect early, significant benefit or harm for patients while the trial is in progress. In consultation with NICHD, the study team will convene the DSMB consisting of at least 5 members with expertise in the following 5 areas: 1) the specific disease(s) under study, 2) biostatistics, 3) epidemiology, 4) ethics/patient advocacy, 5) clinical trials. Board membership should consist of persons completely independent of the investigators who have no financial, scientific, or other conflict of interest with the trial. The DSMB will convene annually to review study progress and safety and may be called into ad hoc sessions as the Board sees fit or at the request of the study Principal Investigator or NICHD. At the annual meeting, the study Statistician and Principal Investigator will present the trial progress and safety and efficacy data, including the results of any planned interim analyses, to the DSMB for consideration. Following its meetings, the DSMB will present its recommendations in writing to continue, modify, or terminate the trial to NICHD and the study Principal Investigator.

9.4.2. Interim Analyses and Stopping Guidelines

Over the course of the trial we will perform one interim analysis in addition to the final analysis for a total of two sequential evaluations of primary study outcome for hypothesis 1. The interim analysis will be performed when the study has accrued approximately one half of the estimated total person time. For the interim analysis, a standardized test statistic will be calculated for the main outcome (malaria incidence) based on accrued data. If this statistic exceeds the nominal critical value calculated using the error spending function (Table 11), then a statistically significant result will have been achieved at the time of that analysis. The sponsor will be notified and a report submitted for review to the Data and Safety Monitoring Board (DSMB). In each interim analysis, the study team will present information on recruitment and the results of interim analyses to the DSMB which will review the data and recommend a course of action.

Table 11. Schedule of interim analyses and boundaries to monitor study outcome

% of Estimated Total Person Time Accrued	Lower Bound	Upper Bound	Alpha	Cumulative Alpha
50%	-2.9626	2.9626	0.00153	0.00305
100%	-1.9686	1.9686	0.02450	0.05000

This analysis assumes α=0.05 (two-sided test), O'Brien-Fleming boundaries (DeMets error-spending function), and 100 trial participants in each arm to be compared.

The DSMB will determine whether to stop the study for early evidence of intervention inferiority or safety concerns after a thorough review of interim data. The DSMB's decision to stop the study will be guided by the critical values shown in Table 10. If, during an interim analysis, the critical value crosses the O'Brien-Fleming boundary indicating that the daily TS arm is superior to the monthly SP and monthly DP arms, we will recommend to the DSMB that the study be stopped on statistical grounds, as a significant difference in favor of the arm expected to be least effective will have been observed. To evaluate trends in adverse events between the annual reviews of the DSMB, the study team will provide interim safety reports to the NICHD Medical Officer, and a DSMB representative, pending discussion of the overall safety monitoring plan with the DSMB committee. These reports will provide cumulative enrollment figures, cumulative deaths and adverse events (classified according to grade), sorted by study arm. Brief clinical descriptions of key events will also be provided. If concerns arise from these interim reports, they will be forwarded to the full DSMB for review. In the event of early study termination, formal reports will be submitted to the NICHD, and local IRBs.

9.5. Rationale, Sample Size, and Analysis Plan for Parasite Culturing and Post-Malaria Phlebotomy

Key single nucleotide polymorphisms (SNPs) are associated with diminished response of malaria parasites to aminoquinoline, quinoline methanol, and possibly artemisinin drugs. We hypothesize that heavy use of antimalarials, in the context of repeated treatment with the antimalarials used for chemoprevention, will select for additional SNPs that mediate diminished response to the drugs. We further hypothesize that novel SNPs will initially be seen only rarely, although once they enter the circulating population of parasites in a community they may increase in prevalence and have major public health importance. For any novel SNPs identified, it will be important to search for associations between the SNP and altered sensitivity of parasites to relevant antimalarial drugs. The only means to directly test the sensitivity of a parasite line to antimalarial drugs is to test sensitivity of parasites cultured in vitro. Parasites are cultured with varied concentrations of drugs of interest, and for each drug the IC_{50}, the concentration at which the drug inhibits parasite development by 50%, is determined. Once in vitro sensitivity or resistance has been established for a parasite line, we can test for possible association between SNPs found in the genomes of these parasites and increased in vitro resistance to each drug using a t-test.

For our sample size calculation, we predict that 1% of collected samples will contain novel SNPs. This estimate is supported by initial evaluation for a single SNP that mediates antifolate resistance and is known to be rare in Africa, dihydrofolate reductase (dhfr) I164L. In Kampala, this SNP has been seen in 1 of 44 (2.3%) children in our study population receiving daily trimethoprim/sulfamethoxazole (TS) and 1 of 121 (0.8%) children in our study population not receiving daily TS. SNP assessment will be performed by standard molecular techniques available in our laboratory, using DNA extracted from finger-prick blood obtained with each episode of malaria identified in our study. Sample size calculations for children to receive phlebotomy for culturing of parasites are as follows. For P. falciparum strains recently studied in Kampala, the mean

IC_{50} values for dihydroartemisinin (DHA) (which is also the active metabolite of artemether), lumefantrine (LUM), and piperaquine (PIP) were 0.74 nmol/L, 0.73 nmol/L, and 8.56 nmol/L, respectively. Clinically relevant cut-offs for resistance for DHA, LUM, and PIP are estimated to be 7 nmol/L, 10 nmol/L, and 30 nmol/L, respectively. To approximate the statistical power that can be expected for the analyses of the IC_{50} values, we employed normal approximations for the outcome defined as log(IC_{50}). Data from other cohort studies done in Ugandan children by our group show that this outcome measure is approximately normally distributed. Following log transformation, the mean values and standard deviations for the sensitive strains are as follows: DHA= -0.25 nmol/L (SD= 0.31), LUM= -0.29 nmol/L (SD= 0.32), and PIP= 0.79 nmol/L (SD= 0.35). The log transformation of the clinically relevant cut-offs for resistance strains for DHA, LUM, and PIP are estimated to be 0.85 nmol/L, 1.0 nmol/L, and 1.48 nmol/L, respectively For DHA, in order to have 90% power (alpha=0.05) to detect a mean difference in the IC_{50} of 1.10 nmol/L (with an estimated standard deviation equal to that of the sensitive controls) we will require 2 strains with in vitro DHA resistance. For LUM, in order to have 90% power (alpha=0.05) to detect a mean difference in the IC_{50} of 1.39 nmol/L (with an estimated standard deviation equal to that of the sensitive controls) we will require 2 strains with in vitro LUM resistance. For PIP, in order to have 90% power (alpha=0.05) to detect a mean difference in the IC_{50} of 0.7 nmol/L (with an estimated standard deviation equal to that of the sensitive controls) we will require 4 strains with in vitro PIP resistance. Since an estimated 1% of our malaria episodes will contain a novel SNP in relevant genes that may confer resistance (pfcrt or pfmdr1), we will need to culture 400 parasite strains in order to obtain 4 strains with in vitro resistance. Our past experience with parasite culture indicate that we can successfully culture approximately 60% of all malaria episodes, dependent on parasite density and timing of presentation to the clinic. However, given that this Tororo lab is a new facility with new equipment and newly-trained staff, we estimate that we will be able to successfully culture approximately 40% of all malaria episodes. Therefore, we will require a total sample size of 1000 episodes of malaria over the course of the study to analyze the relationship between in vitro drug-resistance and resistance-mediating SNPs.

For analysis, we will consider each novel SNP to be a case and all strains with wild type genotypes to be the controls. We will match each case with three controls randomly generated from our list of strains with wild type genotypes at the position of the novel SNP. Matching controls will be chosen using randomly generated numbers and then matching these numbers to a numerical list of wild type strains. We will test the association between the mean log IC50 of the strains with novel SNPs and the strains with wild type genotypes using a t-test. We will also test the association between the outcomes of antimalarial therapy involving strains with novel SNPs and wild type using Fisher's exact test.

10. DATA COLLECTION AND MONITORING

10.1. Record Keeping

All clinical data will be recorded onto standardized case record forms (CRFs) by study physicians. Laboratory data will be recorded in a laboratory record book by the study laboratory technologists and then transferred to the case record forms by study coordinators, who will review the case record forms frequently for completeness and accuracy. Data will be entered directly from CRFs into a computerized database or transferred from the CRFs onto standardized data extraction forms and then into a computerized database. All computerized data will be double entered to verify accuracy of entry. Electronic data including all study databases and supporting electronic documentation will be archived to large-scale digital tape on a daily basis. On a monthly basis, a complete backup tape will be transported off-site to the Kampala Data Management Center for rotating secure storage. In addition, the database from the backup will be placed onto one of the Kampala DMC servers as a data mirror for read-only access in the event that the Tororo web-site becomes temporary unavailable.

10.2. Data Quality Assurance and Monitoring

In order to insure data quality, the study Data Manager will perform a quarterly data quality audit. For this audit a 1% random sample of study forms entered into the data management system from the previous 2 weeks will be selected and compared for accuracy with the original case-report forms and source documents. In addition the study the Data Manager will perform monthly reviews of the 100% double data entry data verification logs and the data management system audit trail log to identify potential data quality issues**.** The data will be owned by the Makerere University-University of California, San Francisco Research Collaboration.

11. HUMAN SUBJECTS

11.1. Subject Selection Criteria

Study subjects will be HIV-uninfected children born to HIV-infected and HIV-uninfected mothers who meet our selection criteria and whose parents or guardians provide informed consent. Study subjects will be enrolled at 4-5 months of age and followed until they reach 36 months of age. We plan to recruit only Ugandan residents and will recruit male and female children. This population is most appropriate for study, as malaria is primarily a pediatric disease in highly endemic countries such as Uganda. In addition, children have less antimalarial immunity than adults, due to a smaller number of past infections, and so are likely the group that will benefit most from chemoprevention.

11.2. Risks and Discomforts

11.2.1. Privacy

Care will be taken to protect the privacy of subjects and parents/guardians, as described in this protocol. However, there is a risk that others may inadvertently see patients' medical information, and thus their privacy may be compromised.

11.2.2. Finger Pricks, Heel Sticks, and Venipuncture

Risks of these procedures include pain, transient bleeding and soft-tissue infection.

11.2.3. Risks of Study Medications

11.2.3.1. Risk of Trimethoprim-Sulfamethoxazole

TS has been licensed, in use, and under study for decades. Thus, there are an abundance of studies that investigate the use and safety profile of TS. Most of the adverse events associated with use of TS occur at higher doses and with a mean onset in the first 10-14 days of initiation of the medication [55]. Additionally, HIV patients, and especially those who are at lower CD4 counts, tend to have more adverse reactions to TS [56]. Even in children, in whom adverse reactions are less prevalent, concomitant HIV infection appears to predispose toward increased adverse events, though this finding may have been distorted by lower doses used in a comparison group on urinary prophylaxis [57].

Not surprisingly, in examining a multitude of studies, there are a wide range of results assessing the overall tolerability of TS. At one end of the spectrum, one study followed HIV patients with CD4 counts<200 cells/mm^3 on TS prophylaxis for 36 months, reporting that only 21% of the patients initially started on the drug were able to remain on the drug at the original dose [58]. The primary side effects leading to discontinuation or changing of the doses were leukopenia (26%), fever (20%), rash (16%), and gastroenterologic distress (10%). In contrast, a more recent study from Malawi followed 767 HIV-infected patients with active tuberculosis receiving either single strength or double strength TS once a day for eight months [59]. No differences were demonstrated in survival or adverse events between the two groups, with only 37 patients having any adverse events, 36 of whom had mild to moderate symptoms and did not require cessation of the drug. One patient developed Stevens-Johnson syndrome, which regressed following discontinuation of the drug. Despite the variety of reports regarding adverse events, most studies reviewed found sulfonamides to be safe drugs for use in long-term preventive therapy.

The primary severe adverse events related to chronic TS use are rare, even with treatment doses of the drug [60]. The quoted prevalences of these rare manifestations are based on large database analysis and are not necessarily related to chronic prophylactic therapy. The severe adverse events can be divided into three categories: cutaneous, hematologic, and hepatic. Cutaneous reactions to TS are the most common and well-known reactions. The rate in children less than 2 years of age is 7.4 events per 100 years at risk [61]. The spectrum of disease includes urticaria, papular rash, fixed drug eruption, erythema multiforme, Stevens-Johnson syndrome (SJS), and toxic epidermal necrolysis (TEN). The most common rashes are urticaria and papular rash and are usually hypersensitivity reactions that regress upon cessation of drug. Erythema multiforme, SJS, and TEN are rare acute bullous diseases with similar pathophysiology. Erythema multiforme secondary to TS was not reported in a population-based study of almost 300,000 people,[62] but there have been case reports of the syndrome related to TS use [63]. The overall incidence of SJS in large population studies was 1.2 to 6.1 cases per

million person-years, with only 3 pediatric cases possibly related to TS use [62]. The overall incidence of TEN was 0.4 to 1 case per million person-years, though the true incidence of TEN related to TS in these cases is unclear [62]. Hematologic manifestations appear to be caused by a dose-dependent decrease in erythroid and granulocyte-monocyte colony formation [64]. The estimated incidence of blood dycrasias appears to vary greatly depending upon the dose and length of therapy. The largest population study of 2,622 children on TS therapy showed no blood dyscrasias, but the average number of medication refills was only two [65]. Hepatotoxicity is also a rare adverse event, with substantial increase in liver transaminases reported to occur in 1/11,000 to 1/45,000 treatments with TS in adults [66, 67]. In reviews of children placed on TS, there were no cases of hepatotoxicity noted in either Sweden, Finland, or the USA [61, 65]. There were, however, 6 individual case reports, of children aged 16 months to 16 years with hepatotoxicity thought to be related to TS or SP, four with resolution of symptoms and two with liver failure [68-71]. These studies suggest that the most common adverse events to TS are mild and self-limited, while serious adverse events are extremely rare and usually reversible.

11.2.3.2. Risk of Sulfadoxine-Pyrimethamine

Although technically a combination regimen, SP is generally considered a single antimalarial agent, as its success depends on the synergistic action of its two component inhibitors of folate synthesis. SP is approved in the USA for the treatment of falciparum malaria and for chemoprophylaxis against malaria in travelers, but it is no longer recommended for this second use due to rare, but serious toxicity. Adverse reactions listed on the SP package insert (Roche, USA) are blood dyscrasias (agranulocytosis, aplastic anemia, thrombocytopenia), allergic reactions (erythema multiforme and other dermatological conditions), gastrointestinal reactions (glossitis, stomatitis, nausea, emesis, abdominal pain, hepatitis, diarrhea), central nervous system reactions (headache, peripheral neuritis, convulsions, ataxia, hallucinations), respiratory reactions (pulmonary infiltrates), and miscellaneous reactions (fever, chills, nephrosis); based on widespread experience with the drug, all of these reactions appear to be uncommon or rare with short-term therapeutic use. The best-documented severe adverse effects with SP are cutaneous reactions, primarily noted when SP was used for long-term chemoprophylaxis in non-African populations. Reported rates of serious reactions to SP in the UK, with long-term use for chemoprophylaxis, were 1:2100, with 1:4900 serious dermatological reactions and 1:11,100 deaths [72]. Estimated rates of toxicity in the US were 1:5000-8000 severe cutaneous reactions and 1:11,000-25,000 deaths [73]. Clinical experience suggests that risks of severe toxicity are much lower with malaria treatment regimens in Africa.

Several centers of malaria research formed a consortium to conduct a robust and comprehensive assessment of use of intermittent preventive treatment of malaria in infants (IPTi) using SP (http://www.ipti-malaria.org/). To date 6 randomized controlled trials have been published evaluating the use of IPTi with SP at the time of routine immunizations. In a pooled analysis of these 6 trials, approximately 4,000 infants have received 12,000 doses of IPTi with SP (http://www.ipti-malaria.org/). The drug was found to be well tolerated and safe, with significantly fewer serious adverse events in the

SP group compared to the placebo group. Two SP recipients were reported to have developed SJS after the third dose. Both of these children were seen at home by a general physician who noted bullous skin lesions, but did not recommend hospitalization. The children were examined approximately 2 weeks later and were found to be well. As SJS is a severe disorder requiring intensive care and is associated with a high case-fatality rate, the investigators questioned the validity of the diagnosis of SJS in these two children.

11.2.3.3. Risk of Dihydroartemisinin-Piperaquine

DP is an artemisinin-containing fixed-combination drug developed in China. Recent randomized clinical trials in Cambodia, Vietnam, and Thailand indicated excellent tolerability and high cure rates against multi-drug resistant falciparum malaria. Artemisinin derivatives such as dihydroartemisinin have been used safely in large numbers of patients with uncomplicated or severe malaria [74]. Piperaquine has been used less widely, but it was a standard antimalarial drug in China from the 1960s-1980s when over 140 million doses were distributed and used for the treatment and prevention of malaria. Data from in vitro and animal studies suggest that it is as potent as chloroquine but less toxic [75, 76]. In the first human studies of piperaquine, the main side effects were mild headache, listlessness, nausea, and dizziness [77]. In a study of the safety and efficacy of DP in 106 Cambodian children and adults with uncomplicated malaria, adverse events were uncommon (< 5%), mild, short lived, and difficult to distinguish from symptoms of malaria (anorexia, nausea, vomiting, abdominal pain, diarrhea, and dizziness) [78]. In a safety evaluation of DP in 62 Cambodian children and adults with malaria, DP was found to be safe and well tolerated with no evidence of clinically significant postural hypotension, QTc prolongation, or propensity for hypoglycemia [79]. In a clinical trial of DP in 166 Vietnamese patients with uncomplicated malaria, 3% of patients reported minor adverse events, mostly transient nausea, which were self limited and resolved with the abatement of fever [80]. In a dose-optimization clinical trial of DP in 487 children and adults from Thailand with uncomplicated malaria, DP was well tolerated, with a low incidence of mild adverse events, which were mainly upper gastrointestinal and were similar to those reported in other studies [81]. In a clinical trial of DP in 331 children and adults from Thailand with uncomplicated malaria, DP was well tolerated, with a low incidence of mild side effects and no serious adverse events felt to be related to the study drug [82]. Our group has completed 3 clinical trials including DP for the treatment of uncomplicated falciparum malaria in Ugandan children 6 months to 10 years of age. A total of 777 DP treatments have been observed resulting in excellent tolerability and no serious adverse events related to the drug [47, 49].

11.3. Treatment and Compensation for Injury

If the participant is injured as a result of being in this study, treatment will be available through Tororo District Hospital. The costs of the treatment may be covered by the study sponsor, NICHD, depending on a number of factors. Makerere University, UCSF, and NICHD do not normally provide any other form of compensation for injury.

11.4. Costs to the Subjects

There will be no cost to the participant or their parents/guardians for participation in this study.

11.5. Reimbursement of Subjects

Participants will not be paid for their participation in the study. We will provide all routine medical care, including evaluations, medications available in our clinic, and cost of any transportation free of charge. In addition, we will reimburse the cost of consultation for referrals made by study physicians to other clinics and services within Tororo District Hospital and visits the cost of most diagnostic tests (including laboratory test, X-rays, and ultrasounds) and medications resulting from referrals by the study team, using available funds. However, reimbursement of all diagnostic tests and treatment recommended outside the study clinic cannot be guaranteed in all circumstances.

11.6. Institutional Review Board (IRB) Review and Informed Consent

This protocol, all procedures and consent forms, and any subsequent modifications must be reviewed and approved by the IRBs of all the participating institutions in both the U.S. and in Uganda. This includes the UCSF Committee on Human Research (CHR), the MU School of Medicine - Research and Ethics Committee (SOM-REC), and the Uganda National Council of Science and Technology (UNCST).

All consent forms will be translated into the local language (Jopadhola, Teso, Swahili, Luganda, and English) and back-translated into English to ensure correct use of language. Consent forms will be read aloud to parents by trained study interviewers. The informed consent will describe the purpose of the study, all the procedures involved, and the risks and benefits of participation. Interviewers will ask parents/guardians of study participants to summarize the study and explain the reasons why they want to participate. Either a signature or a thumbprint (for parents/guardians who cannot read) will be acceptable to confirm informed consent for participation in the study.

11.7 Definition of Parent/Guardianship.

For this project, we will define a parent as someone who attests that he/she is the biological parent of the potential participant. However, has been found in Uganda that a high number of the HIV-infected children have lost one or both parents. These children live with caretakers who do not have documented formal guardianship status because there is no formal, legal guardian system in Uganda. In Uganda, orphan children are customarily cared for by one or more relatives; a single individual family member is not usually identified as the sole guardian or custodian of the child. We will define a guardian as someone who identifies him/herself as the primary caregiver who is able to make all health care decisions for the potential participant. A guardian must be at least 18 years of

age, however; a parent may be less than 18 years of age. These definitions are currently approved for use in current research projects conducted in Uganda following extensive discussion with the Ugandan Ministry of Justice, the Uganda National Council of Science, the Makerere University Research and Ethics Committee (IRB), and the NIH in 2006.

11.8 Study Discontinuation

This study may be discontinued at any time by the NIH, respective IRBs or other Governmental agencies in the United States or Uganda as part of their duties to ensure that research subjects are protected. Furthermore, A Data Safety and Monitoring Board (DSMB) will be established by the study team in cooperation with the NIH to assess at specified intervals the progress of the clinical trial, safety data and critical efficacy variables, and will recommend to the NIH whether to continue, modify or terminate the trial. Over the course of the trial we will perform two interim analyses in addition to the final analysis for a total of three sequential evaluations of study outcome. We will perform the two interim analyses when the study has accrued approximately one third and then two thirds of the study subjects. We will submit a report to the DSMB which will review the data and recommend a course of action. The DSMB will determine whether to stop the study for early evidence of intervention inferiority or safety concerns after a thorough review of interim data.

12. PUBLICATION OF RESEARCH FINDINGS

The findings from this study may be published in a medical journal. No individual identities will be used in any reports or publications resulting from the study. The researchers will publish results of the trial in accordance with NICHD, UNCST, UCSF, and Makerere University guidelines.

13. BIOHAZARD CONTAINMENT

As the transmission of HIV and other blood-borne pathogens can occur through contact with contaminated needles, blood, and blood products, appropriate blood and secretion precautions will be employed by all personnel involved in the drawing of blood, exposure to blood and secretions, and shipping and handling of all specimens for this study. We will follow the current guidelines set forth by the Centers for Disease Control and Prevention and the NIH. All infectious specimens will be transported using packaging mandated in the Federal Code of Regulations, CDC 42 CFR Part 72.

14. REFERENCES

1. Okello PE, Van Bortel W, Byaruhanga AM, et al. Variation in malaria transmission intensity in seven sites throughout Uganda. Am J Trop Med Hyg 2006;75:219-25
2. Organization WH. World Malaria Report, 2005. Geneva, Switzerland: World Health Organization, 2006

3. Lynch KI, Beach R, Asamoa K, Adeya G, Nambooze J and Janowsky E. President's Malaria Initiative, Rapid Assessment Report - Uganda, 2005
4. Lalloo DG, Olukoya P and Olliaro P. Malaria in adolescence: burden of disease, consequences, and opportunities for intervention. Lancet Infect Dis 2006;6:780-93
5. Sachs J, Malaney P. The economic and social burden of malaria. Nature 2002;415:680-5
6. Rogier C. Natural history of Plasmodium falciparum malaria and determining factors of the acquisition of antimalaria immunity in two endemic areas, Dielmo and Ndiop (Senegal). Bull Mem Acad R Med Belg 2000;155:218-26
7. White NJ. Intermittent presumptive treatment for malaria. PLoS Med 2005;2:e3
8. Snow RW, Craig M, Deichmann U and Marsh K. Estimating mortality, morbidity and disability due to malaria among Africa's non-pregnant population. Bull World Health Organ 1999;77:624-40
9. Rogier C, Tall A, Diagne N, Fontenille D, Spiegel A and Trape JF. Plasmodium falciparum clinical malaria: lessons from longitudinal studies in Senegal. Parassitologia 1999;41:255-9
10. Lengeler C. Insecticide-treated bed nets and curtains for preventing malaria. Cochrane Database Syst Rev 2004:CD000363
11. Alonso PL, Sacarlal J, Aponte JJ, et al. Efficacy of the RTS,S/AS02A vaccine against Plasmodium falciparum infection and disease in young African children: randomised controlled trial. Lancet 2004;364:1411-20
12. Aponte JJ, Aide P, Renom M, et al. Safety of the RTS,S/AS02D candidate malaria vaccine in infants living in a highly endemic area of Mozambique: a double blind randomised controlled phase I/IIb trial. Lancet 2007;370:1543-51
13. Wolde B, Pickering J and Wotton K. Chloroquine chemoprophylaxis in children during peak transmission period in Ethiopia. J Trop Med Hyg 1994;97:215-8
14. Greenwood BM, Greenwood AM, Bradley AK, et al. Comparison of two strategies for control of malaria within a primary health care programme in the Gambia. Lancet 1988;1:1121-7
15. Hogh B, Thompson R, Lobo V, et al. The influence of Maloprim chemoprophylaxis on cellular and humoral immune responses to Plasmodium falciparum asexual blood stage antigens in schoolchildren living in a malaria endemic area of Mozambique. Acta Trop 1994;57:265-77
16. Menendez C, Kahigwa E, Hirt R, et al. Randomised placebo-controlled trial of iron supplementation and malaria chemoprophylaxis for prevention of severe anaemia and malaria in Tanzanian infants. Lancet 1997;350:844-50
17. Watkins WM, Brandling-Bennett AD, Oloo AJ, Howells RE, Gilles HM and Koech DK. Inadequacy of chlorproguanil 20 mg per week as chemoprophylaxis for falciparum malaria in Kenya. Lancet 1987;1:125-8
18. Faucher JF, Binder R, Missinou MA, et al. Efficacy of atovaquone/proguanil for malaria prophylaxis in children and its effect on the immunogenicity of live oral typhoid and cholera vaccines. Clin Infect Dis 2002;35:1147-54
19. Greenwood BM, David PH, Otoo-Forbes LN, et al. Mortality and morbidity from malaria after stopping malaria chemoprophylaxis. Trans R Soc Trop Med Hyg 1995;89:629-33

20. Anglaret X, Chene G, Attia A, et al. Early chemoprophylaxis with trimethoprim-sulphamethoxazole for HIV-1-infected adults in Abidjan, Cote d'Ivoire: a randomised trial. Cotrimo-CI Study Group. Lancet 1999;353:1463-8
21. Wiktor SZ, Sassan-Morokro M, Grant AD, et al. Efficacy of trimethoprim-sulphamethoxazole prophylaxis to decrease morbidity and mortality in HIV-1-infected patients with tuberculosis in Abidjan, Cote d'Ivoire: a randomised controlled trial. Lancet 1999;353:1469-75
22. Chintu C, Bhat GJ, Walker AS, et al. Co-trimoxazole as prophylaxis against opportunistic infections in HIV-infected Zambian children (CHAP): a double-blind randomised placebo-controlled trial. Lancet 2004;364:1865-71
23. Mermin J, Lule J, Ekwaru JP, et al. Effect of co-trimoxazole prophylaxis on morbidity, mortality, CD4-cell count, and viral load in HIV infection in rural Uganda. Lancet 2004;364:1428-34
24. Kamya MR, Gasasira AF, Achan J, et al. Effects of trimethoprim-sulfamethoxazole and insecticide-treated bednets on malaria among HIV-infected Ugandan children. Aids 2007;21:2059-66
25. Thera MA, Sehdev PS, Coulibaly D, et al. Impact of trimethoprim-sulfamethoxazole prophylaxis on falciparum malaria infection and disease. J Infect Dis 2005;192:1823-9
26. ter Kuile FO, van Eijk AM and Filler SJ. Effect of sulfadoxine-pyrimethamine resistance on the efficacy of intermittent preventive therapy for malaria control during pregnancy: a systematic review. Jama 2007;297:2603-16
27. Rosen JB, Breman JG. Malaria intermittent preventive treatment in infants, chemoprophylaxis, and childhood vaccinations. Lancet 2004;363:1386-8
28. Chandramohan D, Owusu-Agyei S, Carneiro I, et al. Cluster randomised trial of intermittent preventive treatment for malaria in infants in area of high, seasonal transmission in Ghana. Bmj 2005;331:727-33
29. Kobbe R, Kreuzberg C, Adjei S, et al. A randomized controlled trial of extended intermittent preventive antimalarial treatment in infants. Clin Infect Dis 2007;45:16-25
30. Mockenhaupt FP, Reither K, Zanger P, et al. Intermittent preventive treatment in infants as a means of malaria control: a randomized, double-blind, placebo-controlled trial in northern Ghana. Antimicrob Agents Chemother 2007;51:3273-81
31. Schellenberg D, Menendez C, Aponte JJ, et al. Intermittent preventive antimalarial treatment for Tanzanian infants: follow-up to age 2 years of a randomised, placebo-controlled trial. Lancet 2005;365:1481-3
32. Watkins WM, Mberu EK, Winstanley PA and Plowe CV. More on 'the efficacy of antifolate antimalarial combinations in Africa'. Parasitol Today 1999;15:131-2
33. Roper C, Pearce R, Bredenkamp B, et al. Antifolate antimalarial resistance in southeast Africa: a population-based analysis. Lancet 2003;361:1174-81
34. ter Kuile FO, Steketee RW. Intermittent preventive treatment in infants--adjusting expectations and seeing opportunity. J Infect Dis 2006;194:269-72
35. Massaga JJ, Kitua AY, Lemnge MM, et al. Effect of intermittent treatment with amodiaquine on anaemia and malarial fevers in infants in Tanzania: a randomised placebo-controlled trial. Lancet 2003;361:1853-60
36. Cisse B, Sokhna C, Boulanger D, et al. Seasonal intermittent preventive treatment with artesunate and sulfadoxine-pyrimethamine for prevention of malaria in Senegalese children: a randomised, placebo-controlled, double-blind trial. Lancet 2006;367:659-67

37. Schellenberg D, Menendez C, Kahigwa E, et al. Intermittent treatment for malaria and anaemia control at time of routine vaccinations in Tanzanian infants: a randomised, placebo-controlled trial. Lancet 2001;357:1471-7
38. Macete E, Aide P, Aponte JJ, et al. Intermittent preventive treatment for malaria control administered at the time of routine vaccinations in mozambican infants: a randomized, placebo-controlled trial. J Infect Dis 2006;194:276-85
39. Grobusch MP, Lell B, Schwarz NG, et al. Intermittent Preventive Treatment against Malaria in Infants in Gabon--A Randomized, Double-Blind, Placebo-Controlled Trial. J Infect Dis 2007;196:1595-602
40. Bakyaita N, Dorsey G, Yeka A, et al. Sulfadoxine-pyrimethamine plus chloroquine or amodiaquine for uncomplicated falciparum malaria: a randomized, multisite trial to guide national policy in Uganda. Am J Trop Med Hyg 2005;72:573-80
41. Bukirwa H, Yeka A, Kamya MR, et al. Artemisinin combination therapies for treatment of uncomplicated malaria in Uganda. PLoS Clin Trials 2006;1:e7
42. Dorsey G, Kamya MR, Ndeezi G, et al. Predictors of chloroquine treatment failure in children and adults with falciparum malaria in Kampala, Uganda. Am J Trop Med Hyg 2000;62:686-92
43. Dorsey G, Njama D, Kamya MR, et al. Sulfadoxine/pyrimethamine alone or with amodiaquine or artesunate for treatment of uncomplicated malaria: a longitudinal randomised trial. Lancet 2002;360:2031-8
44. Dorsey G, Staedke S, Clark TD, et al. Combination therapy for uncomplicated falciparum malaria in Ugandan children: a randomized trial. Jama 2007;297:2210-9
45. Gasasira AF, Dorsey G, Nzarubara B, et al. Comparative efficacy of aminoquinoline-antifolate combinations for the treatment of uncomplicated falciparum malaria in Kampala, Uganda. Am J Trop Med Hyg 2003;68:127-32
46. Kamya MR, Dorsey G, Gasasira A, et al. The comparative efficacy of chloroquine and sulfadoxine-pyrimethamine for the treatment of uncomplicated falciparum malaria in Kampala, Uganda. Trans R Soc Trop Med Hyg 2001;95:50-5
47. Kamya MR, Yeka A, Bukirwa H, et al. Artemether-lumefantrine versus dihydroartemisinin-piperaquine for treatment of malaria: a randomized trial. PLoS Clin Trials 2007;2:e20
48. Yeka A, Banek K, Bakyaita N, et al. Artemisinin versus nonartemisinin combination therapy for uncomplicated malaria: randomized clinical trials from four sites in Uganda. PLoS Med 2005;2:e190
49. Yeka A, Dorsey G, Kamya MR, et al. Artemether-lumefantrine versus dihydroartemisinin-piperaquine for treating uncomplicated malaria: a randomized trial to guide policy in Uganda. PLoS ONE 2008;3:e2390
50. Njama-Meya D, Clark TD, Nzarubara B, Staedke S, Kamya MR and Dorsey G. Treatment of malaria restricted to laboratory-confirmed cases: a prospective cohort study in Ugandan children. Malar J 2007;6:7
51. Dorsey G, Dokomajilar C, Kiggundu M, Staedke SG, Kamya MR and Rosenthal PJ. Principal role of dihydropteroate synthase mutations in mediating resistance to sulfadoxine-pyrimethamine in single-drug and combination therapy of uncomplicated malaria in Uganda. Am J Trop Med Hyg 2004;71:758-63
52. Kyabayinze D, Cattamanchi A, Kamya MR, Rosenthal PJ and Dorsey G. Validation of a simplified method for using molecular markers to predict sulfadoxine-pyrimethamine

treatment failure in African children with falciparum malaria. Am J Trop Med Hyg 2003;69:247-52
53. Kamya MR, Bakyaita NN, Talisuna AO, Were WM and Staedke SG. Increasing antimalarial drug resistance in Uganda and revision of the national drug policy. Trop Med Int Health 2002;7:1031-41
54. Sibley CH, Hyde JE, Sims PF, et al. Pyrimethamine-sulfadoxine resistance in Plasmodium falciparum: what next? Trends Parasitol 2001;17:582-8
55. Schneider MM, Hoepelman AI, Eeftinck Schattenkerk JK, et al. A controlled trial of aerosolized pentamidine or trimethoprim-sulfamethoxazole as primary prophylaxis against Pneumocystis carinii pneumonia in patients with human immunodeficiency virus infection. The Dutch AIDS Treatment Group. N Engl J Med 1992;327:1836-41
56. van der Ven AJ, Koopmans PP, Vree TB and van der Meer JW. Adverse reactions to co-trimoxazole in HIV infection. Lancet 1991;338:431-3
57. Rieder MJ, King SM and Read S. Adverse reactions to trimethoprim-sulfamethoxazole among children with human immunodeficiency virus infection. Pediatr Infect Dis J 1997;16:1028-31
58. Bozzette SA, Finkelstein DM, Spector SA, et al. A randomized trial of three antipneumocystis agents in patients with advanced human immunodeficiency virus infection. NIAID AIDS Clinical Trials Group. N Engl J Med 1995;332:693-9
59. Boeree MJ, Sauvageot D, Banda HT, Harries AD and Zijlstra EE. Efficacy and safety of two dosages of cotrimoxazole as preventive treatment for HIV-infected Malawian adults with new smear-positive tuberculosis. Trop Med Int Health 2005;10:723-33
60. Lawson DH, Paice BJ. Adverse reactions to trimethoprim-sulfamethoxazole. Rev Infect Dis 1982;4:429-33
61. Uhari M, Nuutinen M and Turtinen J. Adverse reactions in children during long-term antimicrobial therapy. Pediatr Infect Dis J 1996;15:404-8
62. Chan HL, Stern RS, Arndt KA, et al. The incidence of erythema multiforme, Stevens-Johnson syndrome, and toxic epidermal necrolysis. A population-based study with particular reference to reactions caused by drugs among outpatients. Arch Dermatol 1990;126:43-7
63. Villiger RM, von Vigier RO, Ramelli GP, Hassink RI and Bianchetti MG. Precipitants in 42 cases of erythema multiforme. Eur J Pediatr 1999;158:929-32
64. Golde DW, Bersch N and Quan SG. Trimethoprim and sulphamethoxazole inhibition of haematopoiesis in vitro. Br J Haematol 1978;40:363-7
65. Jick SS, Jick H, Habakangas JA and Dinan BJ. Co-trimoxazole toxicity in children. Lancet 1984;2:631
66. Beard K, Belic L, Aselton P, Perera DR and Jick H. Outpatient drug-induced parenchymal liver disease requiring hospitalization. J Clin Pharmacol 1986;26:633-7
67. Jick H, Derby LE. A large population-based follow-up study of trimethoprim-sulfamethoxazole, trimethoprim, and cephalexin for uncommon serious drug toxicity. Pharmacotherapy 1995;15:428-32
68. Kraemer MJ, Kendall R, Hickman RO, Haas JE and Bierman CW. A generalized allergic reaction with acute interstitial nephritis following trimethoprim-sulfamethoxazole use. Ann Allergy 1982;49:323-5

69. Simma B, Meister B, Deutsch J, et al. Fulminant hepatic failure in a child as a potential adverse effect of trimethoprim-sulphamethoxazole. Eur J Pediatr 1995;154:530-3
70. Stevenson DK, Christie DL and Haas JE. Hepatic injury in a child caused by trimethoprim-sulfamethoxazole. Pediatrics 1978;61:864-6
71. Zitelli BJ, Alexander J, Taylor S, et al. Fatal hepatic necrosis due to pyrimethamine-sulfadoxine (Fansidar). Ann Intern Med 1987;106:393-5
72. Phillips-Howard PA, West LJ. Serious adverse drug reactions to pyrimethamine-sulphadoxine, pyrimethamine-dapsone and to amodiaquine in Britain. J R Soc Med 1990;83:82-5
73. Miller KD, Lobel HO, Satriale RF, Kuritsky JN, Stern R and Campbell CC. Severe cutaneous reactions among American travelers using pyrimethamine-sulfadoxine (Fansidar) for malaria prophylaxis. Am J Trop Med Hyg 1986;35:451-8
74. Meshnick SR, Taylor TE and Kamchonwongpaisan S. Artemisinin and the antimalarial endoperoxides: from herbal remedy to targeted chemotherapy. Microbiol Rev 1996;60:301-15
75. Chen C, Zheng XY and Guo HZ. [Synthesis of pyronaridine related compounds and comparison of antimalarial activities]. Yao Xue Xue Bao 1993;28:594-8
76. Lan CX, Lin X, Huang ZS, Chen YS and Guo RN. [In vivo sensitivity of Plasmodium falciparum to piperaquine phosphate assayed in Linshui and Baisha counties, Hainan Province]. Zhongguo Ji Sheng Chong Xue Yu Ji Sheng Chong Bing Za Zhi 1989;7:163-5
77. Chen L, Qu FY and Zhou YC. Field observations on the antimalarial piperaquine. Chin Med J (Engl) 1982;95:281-6
78. Denis MB, Davis TM, Hewitt S, et al. Efficacy and safety of dihydroartemisinin-piperaquine (Artekin) in Cambodian children and adults with uncomplicated falciparum malaria. Clin Infect Dis 2002;35:1469-76
79. Karunajeewa H, Lim C, Hung TY, et al. Safety evaluation of fixed combination piperaquine plus dihydroartemisinin (Artekin) in Cambodian children and adults with malaria. Br J Clin Pharmacol 2004;57:93-9
80. Tran TH, Dolecek C, Pham PM, et al. Dihydroartemisinin-piperaquine against multidrug-resistant Plasmodium falciparum malaria in Vietnam: randomised clinical trial. Lancet 2004;363:18-22
81. Ashley EA, Krudsood S, Phaiphun L, et al. Randomized, controlled dose-optimization studies of dihydroartemisinin-piperaquine for the treatment of uncomplicated multidrug-resistant falciparum malaria in Thailand. J Infect Dis 2004;190:1773-82
82. Ashley EA, McGready R, Hutagalung R, et al. A randomized, controlled study of a simple, once-daily regimen of dihydroartemisinin-piperaquine for the treatment of uncomplicated, multidrug-resistant falciparum malaria. Clin Infect Dis 2005;41:425-32

15. APPENDICES

Appendix A. Information Sheet

A STUDY ON PREVENTING MALARIA IN YOUNG CHILDREN

Makerere University and the University of California, San Francisco in the United States are combining efforts in Tororo to study new ways of using drugs to prevent malaria in young children.

- Drugs are not now recommended for the prevention of malaria in Uganda. We want to study 3 drugs currently available in Uganda to see if they can be used to prevent malaria
- Our study clinic is located at Tororo District Hospital next to the antenatal clinic and is open every day from 8:00 am to 5:00 pm
- We want to enroll children younger than 6 months of age and follow them until 3 years of age
- Children in this study will receive free medical care
- We shall also give reimbursement for transport to and from our study clinic

For more information, please come to our study clinic where our doctors will be happy to talk to you and see if your child can be in the study.

Appendix B. Research Participant Main Informed Consent

Study Title:	A Randomised Controlled Trial of Monthly Dihydroartemisinin-piperaquine versus Monthly Sulfadoxine-pyrimethamine versus Daily Trimethoprim-sulfamethoxazole versus No Therapy for the Prevention of Malaria
Funding Source	NIH/NICHD
UCSF-CHR Number:	H9926-33953
SOM-REC Number:	2009-077
UNCST Number:	HS-580
Site of Research:	Tororo District Hospital<br>Tororo, Uganda
U.S. Principal Investigator:	Grant Dorsey, MD, PhD
Ugandan Principal Investigator:	Moses Kamya, MBChB, MMed, MPH
Date:	October 5, 2011

INTRODUCTION

You are being asked to allow your child to participate in this research study because he or she lives in an area of Uganda where malaria is common and he or she is not infected with HIV. If your child is found to be infected with HIV, he or she will be referred for appropriate care within Tororo District Hospital or The AIDS Support Organisation (TASO).

This study is sponsored by the National Institute of Child Health and Human Development (NICHD) in America. Before you decide if you want your child to participate in this study, we want you to know as much as possible about it.

This is a consent form which gives information about this study. The study staff is available to talk more about the study. You can ask questions about this study at any time. If you agree to allow your child to participate, we will ask you to sign the consent form. You will get a copy of this form to keep.

WHY IS THIS STUDY BEING DONE?

The purpose of this study is to see if drugs can be used to prevent malaria in young children. We will compare 3 drugs to the current recommended practice of no drugs for the prevention of malaria. The drugs used in this study are:

- trimethoprim-sulfamethoxazole, commonly called “Co-trimoxazole”
- sulfadoxine-pyrimethamine, commonly called “Kamsidar”
- dihydroartemisinin-piperaquine, commonly called “Duocotexin”

The study will also look at the safety of these drugs for children taking them. All of these drugs are approved for use in Uganda. You should know that these drugs have not been fully studied for the prevention of malaria.

HOW MANY PEOPLE WILL TAKE PART IN THIS STUDY?

About 600 children will take part in this study. None of the children in this study will have HIV. About half of the children enrolled will be born to HIV-infected mothers and about half of the children will be born to mothers who are not infected with HIV.

WHAT WILL HAPPEN IF I AGREE TO HAVE MY CHILD PARTICIPATE IN THE STUDY?

If you agree to have your child in this study, the following will happen:

1. Screening for the Study

1a) The study doctors have already asked you some questions about your child to see if it is possible for your child to enter the study. These questions were about :

- your child's age
- breastfeeding
- your child's ability to take the drugs we are studying
- your willingness for your child to get drugs only from the study clinic or Tororo District Hospital
- how close you live to the study clinic
- if you have plans to move away from Tororo
- if you or your child have HIV
- if you or your child has been tested for HIV, letting us look in your hospital or clinic records for the result

If you or your child has not been tested for HIV we will send you and/or your child for HIV testing to the Tororo Hospital antenatal clinic. HIV testing is free of charge. If we need to check your HIV test results, screening for the study will continue after we receive these results.

1b. After the research staff person reads this consent form to you, your questions have been answered, and if we are sure that your child may be in the study, you may sign the consent form today. To help you decide, you may talk to people you know. Also, you have up to one week to decide if you want your child to be in the study.

1c. After you sign the consent form:

- study doctors will perform an electrocardiogram on your child. This test involves placing stickers on your child's chest to see how their heart is working. If this test is abnormal your child cannot participate in this study and will be referred for further evaluation.
- study doctors will examine your child and you will be asked about his or her medical history
- we will take a blood sample (about 2 teaspoons) from one of your child's veins to look for malaria, measure blood cell counts, measure the sugar level, and check for liver problems
- if you agree, a small amount of this blood will be saved for other tests which may be done in the future. Blood for future use will be discussed in a separate consent form. The future tests will have no effect on your child's health care.

1d. We will give you a study card that tells the doctors which part of the study your child is in. You need to keep this card with you at all times or in a safe place. Make sure that you take it with you to the study clinic or hospital whenever your child gets sick.
1e. If study doctors think your child may have malaria, we will immediately look at your child's blood sample. If your child has malaria, we will treat him or her with Coartem, the recommended treatment for malaria in Uganda. If your child has malaria at screening he or she may still join this study.

2. Study Visits

2a) Once Every 30 Days: We want to see your child at least once every 30 days in the study clinic. This is even if your child has not been sick. We will take a blood sample (a finger prick) at these visits to look for malaria. We will also check your child's physical health and make sure you understand the study and are following our instructions. These visits will take about 15 minutes if your child has no health problems. In a subset of children who have been assigned to receive Duocotecxin, we will repeat the electrocardiogram of the heart while they are taking this drug.
2b) Every 4 Months: At least every 4 months, we will take 2 teaspoons of blood from your child for safety and research reasons to check blood counts, the sugar level, and how the liver is working. We may also do some other laboratory tests for research purposes. We will tell you, in advance, when these visits will be scheduled. If your child misses one of these visits, we will visit you at home and ask you to bring your child to the clinic as soon as possible. We will tell you the results of your child's safety blood tests at the next study visit after they are available.
2c) When Your Child Has a Health Problem: Each time your child seems sick or has any health problem, please bring him or her to our study clinic. Unless the study doctors tell you that you need to take your child somewhere else for treatment, we ask that you bring your child to *our* study clinic only for all health care and medications. There will be someone at the study clinic every day from 8:00 am to 5:00 pm. The clinic will be open on weekends and holidays.
2d) After 5:00 pm: If your child becomes sick after 5:00 pm, please bring him or her to Tororo District Hospital. Please bring your child's study card with you and tell the hospital staff that your child is a part of this study.
2e) How Long are Study Visits: Except for the visits every 30 days, each study visit will last between 30 minutes and 2 hours, depending on your child's health
2f) Health Care at Different Clinics or Hospitals: If you choose to take your child to another clinic or hospital for medical care, we will not cause any problems for you, but we cannot guarantee you will be reimbursed for care given outside of the study.

3. At Each Study Clinic Visit For A New Illness

3a) Study doctors will examine your child and ask you some questions.
3b) If the doctors think your child does not have malaria, they will treat your child for what they think is the problem.
3c) If the doctors think your child may have malaria, they will take a small amount of blood from your child's finger or heel to look for malaria.
3d) If the doctors do not find malaria, they will treat your child or refer your child to another part of Tororo District Hospital for needed care.

3e) If the doctors do find malaria, we will take 2 teaspoons of blood from your child to check blood counts. We may also do other laboratory tests for research purposes.

3f) If the doctors find malaria, we will treat your child at the study clinic with Coartem. Coartem is the drug which the government thinks is the best treatment for non-severe malaria. If your child is found to have severe malaria, he or she will be treated with quinine, the recommended treatment for severe malaria in Uganda. Depending on how sick your child is, treatment for severe malaria may be given at the clinic or your child may be admitted to Tororo District Hospital.

3g) If your child is diagnosed with malaria, we will ask you to bring your child back to the study clinic at least 7 times over the next 4 weeks. These visits will help study doctors see how well your child is responding to the study drug. At each of these visits:

- We will examine your child.
- We will take a small amount of blood from your child's finger or heel to look for malaria.
- If the treatment given to your child is not working well, we will give your child additional treatment through the clinic or Tororo District Hospital.

3h) After your child turns two years of age and is diagnosed with malaria, we will ask you to bring your child back to the study clinic the following 2 days only and will examine your child and take a small amount of blood from your child's finger to look for malaria.

3i) If your child does not come to a scheduled visit, a health worker will visit you at your home. The health worker will bring you and your child to the study clinic or request that you come to the clinic as soon as possible.

3j) If your child is treated at Tororo District Hospital, we will ask you to return the next morning to the study clinic. At the clinic, we will continue follow-up as described above.

4. Amount of Blood to be Collected Each Year

Each year we expect to collect from your child a little over 16 teaspoons of blood. If your child is sick with malaria more than twice in one year, we will take a little more blood. We will collect this blood from a vein in your child's arm or leg and by finger prick or heel stick.

5. Study Drugs for the Prevention of Malaria

The decision about which study drug to give your child for the prevention of malaria will be made by chance, like flipping a coin. You or the study staff will not be able to decide which study drug your child will get. Some children will be given no study drug. Some children will be given one of three different study drugs. If your child is chosen to get a study drug, we ask you to give the drug to your child at home. We will provide you with the drugs and make sure you do not run out. We will also give you instructions on how to give the drugs and give you a calendar to remind you when to give the drugs. About once each month we will ask you questions about whether or not you gave the drugs and when you gave them.

5a) **If your child was born to a mother not infected with HIV**: We will ask you to bring your child to the clinic when he or she reaches 6 months of age. At this time the study staff will decide which of the following treatments your child will receive:

- No treatment
- Co-trimoxazole, given once each day
- Kamsidar, given once each month as a single dose
- Duocotexin, given each month once every day for 3 days in a row

5b) **If your child is born to a mother infected with HIV**: You will be asked to give your child Co-trimoxazole once a day at the time he or she is enrolled in the study. This is recommended for all children who are born to mothers infected with HIV. You will receive counseling on HIV and infant feeding throughout the study.

We will recommend that your child only breastfeed for the first six months of life unless replacement feeding is acceptable, feasible, affordable, sustainable and safe for you and your child before that time. When study doctors think replacement feeding is acceptable, feasible, affordable, sustainable and safe he or she should not breastfeed at all.

When your child reaches six months of age, if replacement feeding is still not acceptable, feasible, affordable, sustainable and safe, continuation of breastfeeding with additional complementary foods will be recommended, and you and your child will continue to be regularly assessed. We will recommend that all breastfeeding should stop once a nutritionally adequate and safe diet without breast milk can be provided.

We will test your child for HIV when he/she turns 18 months of age. About 6 weeks after your child has stopped breastfeeding, we will retest him or her for HIV. We will also test your child for HIV every 60 days while he or she is breastfeeding. You will be asked to bring your child to the clinic about 2 weeks after HIV testing so that we can give you the results.

If your child becomes infected with HIV during breastfeeding, he or she will be taken out of this study and referred for care at Tororo District Hospital or TASO.
If your child did not get HIV during breastfeeding, the study staff will decide which of the following treatments your child will receive during the study:

- stop giving Co-trimoxazole
- continue giving Co-trimoxazole once each day
- stop giving Co-trimoxazole and begin giving Kamsidar once each month as a single dose
- stop giving Co-trimoxazole and begin giving Duocotexin once each day for 3 days in a row every month

HOW LONG WILL MY CHILD BE IN THE STUDY?

If your child is given study drug for the prevention of malaria, we ask you to continue study drug until your child reaches 2 years of age. When your child turns 2 years old, we

will ask you to bring any unused study drug to the clinic. All children enrolled in the study will be followed until they turn 3 years old.

WHY WOULD MY CHILD BE WITHDRAWN FROM THE STUDY EARLY?
You may decide to stop your child from being in the study at any time and for any reason. For your child's safety and health, study doctors might decide he or she should stop being in the study. If either takes place, the study doctors may ask to examine your child and collect about 2 teaspoons of blood for safety tests. If your child leaves the study before finishing and we have enough funds available, we will give your child medical care for any problems that began during the study.

The funding agency can stop the study at any time and for any reason. Your study doctors may also take your child out of the study. These are the possible reasons:

1. If you and your child moves more than 30 km from the study clinic for more than 60 days in a row
2. If we are unable to locate you or your child for more than 60 days in a row
3. If you withdraw your consent to have your child stay in this study
4. If your child is not able to come for his or her study visits
5. If the study doctors think the study is no longer good for your child

WHAT SIDE EFFECTS OR RISKS CAN I EXPECT MY CHILD TO HAVE FROM BEING IN THE STUDY?

1) **Risk of study drugs:** All of the drugs being used for the prevention of malaria in this study may cause side effects. Below is a list of potential side effects for each of the study drugs. Those listed as less likely are mild and occur in fewer than 1 in 5 patients. Those listed as rare but serious occur in fewer than 1 in 100 patients.

1A. **Risks of Co-trimoxazole:**

Less Likely

- nausea ● vomiting ● diarrhea
- itching ● skin rash ● slight decrease in blood counts

Rare but serious and can result in death

- serious skin rash and blisters
- large decrease in blood counts which can make it harder for your child's body to fight infection or disease
- swelling of the liver, which can result in your child becoming weak and have difficulty eating

1B) **Risks of Kamsidar:**

Less Likely

- nausea ● vomiting ● diarrhea
- abdominal pain ● decrease in appetite ● itching
- skin rash ● feeling dizzy ●headache
- weakness ● slight decrease in blood counts

Rare but serious and can result in death

- serious skin rash and blisters
- large decrease in blood counts which can make it harder for your child's body to fight infection or disease
- swelling of the liver, which can result in your child becoming weak and have difficulty eating

1C) **Risks of Duocotexin:**

Less Likely

- nausea
- vomiting
- diarrhea
- abdominal pain
- decrease in appetite
- itching
- skin rash
- feeling dizzy
- slight decrease in blood counts

Rare but serious and can result in death

- large decrease in blood counts which can make it harder for your child's body to fight infection or disease
- swelling of the liver, which can result in your child becoming weak and have difficulty eating
- rapid beating of the heart

2) **Randomisation risks:** Your child will be assigned to a treatment group by chance. The treatment group your child is assigned to may prove to be less effective in the prevention of malaria or to have more side effects than the other treatment groups.

3) **Blood drawing (venipuncture) risks:** Drawing blood from a vein may cause temporary discomfort from the needle stick, bruising, and infection.

4) **Blood drawing (finger stick or heel prick) risks:** The risk of drawing blood by finger stick or heal prick includes temporary discomfort from the needle stick, bruising, skin infection, and fainting. The amount of blood removed will be too small to affect your child's health.

5) **For children born to a mother infected with HIV only – HIV Testing Risks:** Testing your child for HIV may cause you anxiety regardless of the test results. A positive test indicates your child has been infected with the HIV virus, but no one knows for sure when, if ever, your child will become sick with AIDS or a related condition. Receiving positive results may make you very upset. If your child's test is negative, there is still the possibility that your child could be infected with the HIV virus during breastfeeding and test positive at some time in the future. Also, it is always possible that the test results could be wrong.

6) **Unknown Risks:** The experimental treatments may have side effects that no one knows about yet. The researchers will let you know if they learn anything that might make you change your mind about your child participating in the study.

For more information about risks and side effects, please ask your study doctor.

ARE THERE BENEFITS TO TAKING PART IN THE STUDY?

1) The information we get from this study might help Uganda and other countries to decide whether drugs can be used to prevent malaria and, if so, which drug(s) are the best.
2) Your child will be assigned to a treatment program by chance, and the treatment you receive may prove to be more effective than the other study treatments for the prevention of malaria, but this cannot be guaranteed.

WHAT OTHER CHOICES DO I HAVE IF I DO NOT ALLOW MY CHILD TO TAKE PART IN THIS STUDY?

You are free to decide whether or not you want your child in this study. If you decide you do not want your child in the study or decide to stop your child from being in the study at any time and for any reason, this will not affect your child’s care at Tororo District Hospital. If you decide not to take part, your child may still get medical care and get any of these drugs if your doctor thinks they are needed.

WILL MY CHILD’S MEDICAL INFORMATION BE KEPT PRIVATE?

Other people may learn that your child is part of this study because you will get medical care at the study clinic; however, we will not allow people who are not working for the study to see any medical information about your child. Medical information about malaria and other diseases will be collected on your child, but only the people working on the study will see it. Only people involved in this study and officials at the U.S. National Institutes of Health (NIH)/ National Institute of Child Health and Human Development (NICHD) will be able to link your child’s medical records and your child’s study number. The universities and research organisations running this study or making sure that the research is done properly are not allowed to let others know the identity of the people in the study. These organizations include the NIH/NICHD, U.S. Office for Human Research Protections, the U.S. Food and Drug Administration, Committee for Human Research of the University of California, San Francisco, the Makerere University School of Medicine Research Ethics Committee (SOM-REC), and the Uganda National Council on Science and Technology. The medical records for the study will be kept in a locked office and will only be able to be seen by study workers. Your name or your child’s name will not be written in any reports based on this research.

WHAT ARE THE COSTS OF TAKING PART IN THIS STUDY?

There will be no cost for you or your child to take part in this study. Also, we will pay for the cost of all tests or drugs prescribed by our doctors that you may have to purchase outside the clinic. We will also try to pay for tests or drugs prescribed by a doctor to whom we have referred you. This will only be possible if we have the funds available.

WILL I BE PAID FOR TAKING PART IN THIS STUDY?

You or your child will not be paid for participation in the study. You will receive free transportation to the study clinic for all study visits. The amount of reimbursement we give you for transport to the study clinic will be based on current transportation costs from your home. If you agree for your child to be in this study, today you will be given 2

long-lasting insecticide- treated bednets and, as available, a basic care package including a safe water vessel and condoms.

WHAT HAPPENS IF MY CHILD IS INJURED BECAUSE HE OR SHE TOOK PART IN THIS STUDY?

If your child is injured or becomes ill, please contact the doctors at the study clinic. If you have questions about injuries as a result of being in the study, contact the doctors at the study clinic. You can get health care services at Tororo District Hospital in case of such injuries. If funds provided by the study sponsors to carry out this research project are available, you will not have to pay for care for study-related injuries.

WHAT ARE MY CHILD'S RIGHTS IF HE OR SHE TAKES PART IN THIS STUDY?

You are free to decide whether or not you want your child in this study. You have the right to stop your child's participation in the study at any time without penalty or loss of benefits to which you are otherwise entitled.

We will tell you about new information or changes in the study that may affect your child's health or your willingness to have your child continue in the study.

WHO CAN ANSWER MY QUESTIONS ABOUT THE STUDY?

Dr. Kamya and his staff can answer questions you have about the study. You may call Dr. Kamya at Mulago Hospital (mobile: 0712-520469). We will give you free access to a telephone line. You may also contact Prof. James Tumwine (0414-530020) at the Makerere University School of Medicine - Research and Ethics Committee which approved this study for questions about participants' rights and research-related harm.

CONSENT: WHAT YOUR SIGNATURE OR THUMBPRINT MEANS

PARTICIPATION IN RESEARCH IS VOLUNTARY. You have the right to decline to allow your child to participate or to withdraw your child at any point in this study without penalty or loss of benefits to which your child is otherwise entitled. A copy of this consent form will be given to you. Your signature or thumbprint below means that you have had this study explained to you. Your signature or thumbprint below means you have had the opportunity to ask questions and get answers. If you wish your child to participate in this study, you should sign or place your thumbprint below. You will also be asked to sign another form if you want left-over blood specimens to be used for future studies.

Name of Participant (printed)

Name of Parent/Guardian

Signature or Fingerprint * of Parent/Guardian   Date

Name of Study Staff Administering Consent (printed)   Position/Title

---

Signature of Study Staff Administering Consent   Date

Name of Translator (if necessary)

Signature of Translator   Date

*If the parent or guardian is unable to read and/or write, an impartial witness should be present during the informed consent discussion. After the written informed consent form is read and explained to the parent or guardian, and after he or she has orally consented to the child's participation in the trial, and has either signed the consent form or provided his or her fingerprint, the witness should sign and personally date the consent form. By signing the consent form, the witness attests that the information in the consent form and any other written information was accurately explained to, and apparently understood by, the parent or guardian, and that informed consent was freely given by the patient and parent or guardian.

Name of Person Witnessing Consent (printed)

Signature of Person Witnessing Consent   Date

Appendix C. Informed Consent for Future Use of Biological Specimens

Study Title:	A Randomised Controlled Trial of Monthly Dihydroartemisinin-piperaquine versus Monthly Sulfadoxine-pyrimethamine versus Daily Trimethoprim-sulfamethoxazole versus No Therapy for the Prevention of Malaria
Funding Source	NIH/NICHD
UCSF-CHR Number:	H9926-33953
SOM-REC Number:	2009-077
UNCST Number:	HS-580
Site of Research:	Tororo District Hospital<br>Tororo, Uganda
U.S. Principal Investigator:	Grant Dorsey, MD PhD
Ugandan Principal Investigator:	Moses Kamya, MBChB, MMed, MPH
Date:	October 5, 2010

INTRODUCTION

While your child is in this study, there may be blood samples taken from your child that may be useful for future research. These samples will be stored for a long time at one of the universities or research organisations which are running this study. These are: Infectious Disease Research Collaboration and the University of California, San Francisco. Samples may also be shared with investigators at other institutions.
Any use of these stored samples must be approved by an institutional review or ethics board.

WHAT SAMPLES WILL BE USED FOR

Your child's blood will be used to study malaria and other infectious diseases and the response of these diseases to treatment. The information we get from these studies will not affect your child's care.

1. These samples will be used for future research to learn more about malaria and other diseases.
2. Your child's samples will be used only for research. They will not be sold or used for the production of commercial products.
3. We may perform genetic research on these samples. However, no genetic information obtained from this research will be placed in your child's medical records. These samples will be identified only by codes so that they cannot be readily identified with your child.

LEVEL OF IDENTIFICATION

We will code your child's samples using numbers. Therefore, the study workers will not be able to easily find out your child's name. We will not put reports about research done with your child's samples into his or her medical record. We will not allow researchers using your child's samples to know your child's name to the best of our ability.

Researchers who study your child's samples in the future may need to know more about your child, such as age, gender and race. We may give this information to a researcher if it is available from your child's participation in this study. We will not give your child's name of anything that might identify your child personally. We will not ask you to sign another consent form.

RISKS

There are few risks to your child from future use of your child's samples. A potential risk might be the release of information from your child's health or study records. We will not put reports about research done with your child's samples into his or her health record. We will keep these reports with study records. We will keep study records as private and safe as possible.

BENEFITS

There will be no direct benefit to your child from this future research on stored samples. From studying your child's samples we may learn more about malaria or other diseases. We may learn how to prevent them, how to treat them, or how to cure them.

RESEARCH RESULTS/MEDICAL RECORDS

1. We may present results from future research using your child's samples in publications and meetings. If this happens, we will not identify your child's name.
2. We will not give reports from future research done with your child's samples to you or your doctor. We will not put these reports in your child's medical record.

QUESTIONS

Dr. Moses Kamya and staff are available to explain this study to you and answer your questions. If you have any other questions about the information here, you may call Dr. Kamya at (mobile: 0712-520469).

FREEDOM TO REFUSE

You can change your mind at any time about allowing your child's samples to be used for future research. If you change your mind, contact Dr. Moses Kamya (mobile: 0712-520469) or the study team at Tororo District Hospital (telephone: 0454-448840). Our

study team will make sure your child's samples will no longer be made available for research by destroying your child's samples. If you decide for us to destroy your child's samples for future research, we still want your child to stay in the study. We will still allow your child to be in future studies, if you want.

WHAT YOUR SIGNATURE OR THUMBPRINT MEANS

A copy of this consent form will be given to you. Your signature or thumbprint below means that use of your child's specimens for future research has been explained to you. It also means that you have had the opportunity to ask questions. You have also had time to think about if you want your child's samples to be used for future research. If you wish to allow your child's specimens to be used for future research, you should sign or thumbprint below.

____
Name of Participant (printed)

____
Name of Parent/Guardian (printed)

____
Signature or Fingerprint * of Parent/Guardian   Date

____
Name of Study Staff Administering Consent (printed)   Position/Title

____
Signature of Study Staff Administering Consent   Date

____
Name of Translator (if necessary)

____
Signature of Translator   Date

*If the parent or guardian is unable to read and/or write, an impartial witness should be present during the informed consent discussion. After the written informed consent form is read and explained to the parent or guardian, and after they have orally consented to their participation in the trial, the parent or guardian should either sign the consent form or provided their fingerprint, and the witness should sign and personally date the consent form. By signing the consent form, the witness attests that the information in the consent form and any other written information was accurately explained to, and apparently understood by, the parent or guardian, and that informed consent was freely given by the parent or guardian.

____
Name of Person Witnessing Consent (printed)

____
Signature of Person Witnessing Consent   Date

Appendix D. Weight-based Administration of Study Drugs

Weight (kg)	Trimethoprim-Sulfamethoxazole daily dosing		
	20mg/100mg tabs	40mg /200mg /5ml susp.	80mg /400mg tabs
< 5	1 tab	2.5 ml	¼ tab
5-15	-	5 ml	½ tab
> 15-30	-	-	1 tab
**Weight (kg)**	**Dihydroartemisinin-Piperaquine (40mg/320mg tabs) monthly dosing given once a day for 3 consecutive days**		
	**Day 1**	**Day 2**	**Day 3**
≤ 5	¼ tab	¼ tab	¼ tab
6-10	½ tab	½ tab	½ tab
11-14	¾ tab	¾ tab	¾ tab
15-19	1 tab	1 tab	1 tab
20-23	1 ¼ tab	1 ¼ tab	1 ¼ tab
24-25	1 ½ tab	1 ½ tab	1 ½ tab
**Weight (kg)**	**Sulfadoxine-Pyrimethamine (500mg/ 25mg tabs) monthly dosing given as a single dose**		
≤12	½ tab		
13-18	¾ tab		
19-22	1 tab		
23-25	1 ¼ tab		

Appendix E. WHO Criteria for Severe Malaria and Danger Signs

Criteria for severe malaria

- Cerebral malaria - defined as unarousable coma not attributable to any other cause in a patient with falciparum malaria
- Generalized convulsions ($\geq$ 3 convulsions over 24 hours period)
- Severe normocytic anemia (Hb < 5 gm/dL)
- Hypoglycemia
- Metabolic acidosis with respiratory distress
- Fluid and electrolyte disturbances
- Acute renal failure
- Acute pulmonary edema and adult respiratory distress syndrome (ARDS)
- Circulatory collapse, shock, septicemia ("algid malaria")
- Abnormal bleeding
- Jaundice

Danger signs

- Less than 3 convulsions over 24 hour period
- Inability to sit up or stand
- Vomiting everything
- Unable to breastfeed or drink
- Lethargy

Appendix F. WHO Antimalarial Treatment Outcome Classification System

ETF (Early Treatment Failure): Days 0. 1, 2, and 3

- Development of danger signs or severe malaria on Days 0-3 in the presence of parasitemia
- Parasitemia on Day 2 higher than on Day 0, irrespective of temperature
- Parasitemia on Day 3 with temperature $\geq$ 38.0°C (tympanic)
- Parasitemia on Day 3 > 25% of count on Day 0

LPF (Late Parasitological Failure): Days 7 to 28

- Presence of parasitemia on Days 7-28 with temperature < 38.0°C (tympanic) and no history of fever in past 24 hours, without previously meeting any of the criteria for early treatment failure

LCF (Late Clinical Failure): Days 4-28

- Development of danger signs or severe malaria Days 4 to 28 in the presence of parasitemia, without previously meeting any of the criteria of early treatment failure or late parasitological failure
- Temperature $\geq$ 38.0°C (tympanic) or history of fever in past 24 hours on Days 4 to 28 in the presence of parasitemia, without previously meeting any of the criteria of early treatment failure or late parasitological failure

ACPR (Adequate Clinical and Parasitological Response)

- Absence of parasitemia on Day 28, irrespective of temperature, without previously meeting any of the criteria of early treatment failure, late clinical failure, or late parasitological failure

Appendix G. Schedule of Clinical and Laboratory Evaluations

Evaluations	Baseline (enrollment)	Approx. every 30 days	Approx. every 60 days	Approx. every 120 days	New visits for medical problems	Day malaria diagnosed
Informed consent	X					
History and physical	X	X			X	X
CBC	X			X		X^
Glucose	X			X		
AST/ALT	X			X		
Routine assessment	X	X				
Adherence monitoring		X				
Blood smear/filter paper	X	X			X*	X
Parasite culture						X
Immunology studies	X			X		X
Repeat HIV DNA PCR			X†			
ECG in subset of those receiving DP	X	X				
PK Plasma sample/Filter paper in subset of those receiving DP				X**		X

* if fever present

† HIV-exposed study participants during breastfeeding and additionally at 6 weeks after breastfeeding cessation and at 18 months of age

** One PK plasma sample will be done at the time of their first 120 day visit after starting chemoprevention therapy.

^CBC only until 2 years of age then Hgb by HemoCue thereafter on day of malaria diagnosis.

Appendix H. Guidelines for Adverse Event Grading (DAIDS AE grading table for symptoms and DMID AE grading table for laboratory tests)

Estimating Severity Grade

If the need arises to grade a clinical AE that is not identified in the AE grading table, use the category "Estimating Severity Grade".

Determining Severity Grade

If the severity of an AE could fall under either one of two grades (e.g., the severity of an AE could be either Grade 2 or Grade 3), select the higher of the two grades for the AE.

Definitions

Basic Self-care Functions	Activities that are age and culturally appropriate (e.g., feeding self with culturally appropriate eating implement)
LLN	Lower limit of normal
NA	Not Applicable
Operative Intervention	Surgical OR other invasive mechanical procedures
ULN	Upper limit of normal
Usual Social & Functional Activities	Activities that are age and culturally appropriate (e.g., social interactions, play activities, learning tasks, etc.).

DAIDS Toxicity Tables- December 2004

SYMPTOMS LISTED ON CRF

PARAMETER	Grade 1 MILD	Grade 2 MODERATE	Grade 3 SEVERE	Grade 4 LIFE THREATENING
**Temperature (tympanic)**	37.7 – 38.6°C	38.7 – 39.3°C	39.4 – 40.5°C	♏
**Chills**	Symptoms causing no or minimal interference with usual social & functional activities	Symptoms causing greater than minimal interference with usual social & functional activities	Symptoms causing inability to perform usual social & functional activities	N/A
**Fatigue/Malaise**	Symptoms causing no or minimal interference with usual social & functional activities	Symptoms causing greater than minimal interference with usual social & functional activities	Symptoms causing inability to perform usual social & functional activities	Incapacitating fatigue/malaise symptoms causing inability to perform basic self-care functions
**Anorexia**	Loss of appetite without decreased oral intake	Loss of appetite associated with decreased oral intake without	Loss of appetite associated with significant weight loss	Life-threatening consequences OR Aggressive intervention indicated [e.g., tube

		significant weight loss		feeding or total parenteral nutrition (TPN)]
**Unintentional weight loss**	N/A	5 – 9% loss in body weight from baseline	10 – 19% loss in body weight from baseline	≥ 20% loss in body weight from baseline OR Aggressive intervention indicated [e.g., tube feeding]
**Diarrhea**	Transient or intermittent episodes of unformed stools OR Increase of ≤ 3 stools over baseline per 24-hour period	Persistent episodes of unformed to watery stools OR Increase of 4 – 6 stools over baseline per 24-hour period	Bloody diarrhea OR Increase of ≥ 7 stools per 24-hour period OR IV fluid replacement indicated	Life-threatening consequences (e.g., hypotensive shock)
**Dysphagia/odynophagia**	Symptomatic but able to eat usual diet	Symptoms causing altered dietary intake without medical intervention indicated	Symptoms causing severely altered dietary intake with medical intervention indicated	Life-threatening reduction in oral intake
**Vomiting**	Transient or intermittent vomiting with no or minimal interference with oral intake	Frequent episodes of vomiting with no or mild dehydration	Persistent vomiting resulting in orthostatic hypotension OR Aggressive rehydration indicated (e.g., IV fluids)	Life-threatening consequences (e.g., hypotensive shock)
**Seizure**	Seizure, generalized onset with or without secondary generalization, lasting< 5 minutes with < 24 hours post ictal state	Seizure, generalized onset with or without secondary generalization, lasting 5 – 20 minutes with < 24 hours post ictal state	Seizure, generalized onset with or without secondary generalization, lasting > 20 minutes	Seizure, generalized onset with or without secondary generalization, requiring intubation and sedation
**Cough**	Transient-intermittent	Persistent-constant	Uncontrolled	Cyanosis, stridor, severe SOB
**Pain** (indicate body site)	Pain causing no or minimal interference with usual social & functional activities	Pain causing greater than minimal interference with usual social & functional activities	Pain causing inability to perform usual social & functional activities	Disabling pain causing inability to perform basic self-care functions OR Hospitalization (other than emergency room visit) indicated

PHYSICAL FINDINGS LISTED ON CRF

PARAMETER	Grade 1 MILD	Grade 2 MODERATE	Grade 3 SEVERE	Grade 4 LIFE THREATENING
**Jaundice**	Slight yellowing of sclera and conjunctiva	Moderate yellowing of sclera, conjunctiva, mucous membranes	Severe yellowing of sclera, conjunctiva, skin	N/A
**Pallor**	Minimally pale conjunctiva, nail beds	Moderately pale conjunctiva, nail beds	Paper white conjunctiva, nail beds, palms	N/A
**Respiratory (RESP)**				
**Dyspnea or respiratory distress**	Wheezing OR minimal increase in respiratory rate for age	Nasal flaring OR Intercostal retractions OR Pulse oxmetry 90– 95%	Dyspnea at rest causing inability to perform usual social & functional activities OR Pulse oximetry < 90%	Respiratory failure with ventilatory support indicated
**CARDIOVASCULAR (CV)**				

**Hemorrhage**<br><br>(significant acute blood loss)	N/A	Symptomatic AND No transfusion indicated	Symptomatic AND Transfusion of $\leq$ 10 cc/kg packed RBCs indicated	Life-threatening hypotension OR Transfusion of > 10 cc/kg packed RBCs indicated
**Hypertension**	N/A	91st – 94th percentile adjusted for age, height, and gender (systolic and/or diastolic)	≥ 95th percentile adjusted for age, height, and gender (systolic and/or diastolic)	Life-threatening consequences (e.g., malignant hypertension) OR Hospitalization indicated (other than emergency room visit)
**Hypotension**	N/A	Symptomatic, corrected with oral fluid replacement	Symptomatic, IV fluids indicated	Shock requiring use of vasopressors or mechanical assistance to maintain blood pressure
**Cardiac arrhythmia (general)**<br>**(By ECG or physical exam)**	Asymptomatic AND No intervention indicated	Asymptomatic AND Non-urgent medical intervention indicated	Symptomatic, non-life-threatening AND Non-urgent medical intervention indicated	Life-threatening arrhythmia OR Urgent intervention indicated
**Cardiac-ischemia/infarction**	NA	NA	Symptomatic ischemia (stable angina) OR Testing consistent with ischemia	Unstable angina OR Acute myocardial infarction
**Prolonged QTc**	Asymptomatic, QTc interval 0.450 – 0.464 sec	Asymptomatic, QTc interval 0.465 – 0.479 sec	Asymptomatic, QTc interval ≥ 0.480 sec	Life-threatening consequences, e.g. Torsade de pointes or other associated serious ventricular dysrhythmia
**Prolonged PR interval**	1st degree AV block (PR > normal for age and rate)	Type I 2nd degree AV block	Type II 2nd degree AV block	Complete AV block
		**GASTROINTESTINAL (GI)**		
**Ascites**	Asymptomatic	Symptomatic AND Intervention indicated (e.g., diuretics or therapeutic paracentesis)	Symptomatic despite intervention	Life-threatening consequences
**Mucositis/stomatitis**	Erythema of the mucosa	Patchy pseudomembranes or ulcerations	Confluent pseudomembranes or ulcerations OR Mucosal bleeding with minor trauma	Tissue necrosis OR Diffuse spontaneous mucosal bleeding OR Life-threatening consequences (e.g., aspiration, choking)
**Pancreatitis**	N/A	Symptomatic AND Hospitalization not indicated (other than emergency room visit)	Symptomatic AND Hospitalization indicated (other than emergency room visit)	Life-threatening consequences (e.g., circulatory failure, hemorrhage, sepsis)
		**SKIN – DERMATOLOGICAL**		
**Alopecia**	Thinning detectable by study participant (or by caregiver for young children and disabled adults)	Thinning or patchy hair loss detectable by health care provider	Complete hair loss	N/A

**Rash** – Cutaneous reaction	Localized macular rash	Diffuse macular, maculopapular, or morbilliform rash OR Target lesions	Diffuse macular, maculopapular, or morbilliform rash with vesicles or limited number of bullae OR Superficial ulcerations of mucous membrane limited to one site	Extensive or generalized bullous lesions OR Stevens-Johnson syndrome OR Ulceration of mucous membrane involving two or more distinct mucosal sites OR Toxic epidermal necrolysis (TEN)
**Hyperpigmentation**	Slight or localized	Marked or generalized	N/A	N/A
**Hypopigmentation**	Slight or localized	Marked or generalized	N/A	N/A
**MUSCULOSKELETAL (MS)**				
**Arthritis**	Stiffness or joint swelling causing no or minimal interference with usual social & functional activities	Stiffness or joint swelling causing greater than minimal interference with usual social & functional activities	Stiffness or joint swelling causing inability to perform usual social & functional activities	Disabling joint stiffness or swelling causing inability to perform basic self-care functions
**NEUROLOGIC (NEURO)**				
**Altered Mental Status**	Changes causing no or minimal interference with usual social & functional activities	Mild lethargy or somnolence causing greater than minimal interference with usual social & functional activities	Confusion, memory impairment, lethargy, or somnolence causing inability to perform usual social & functional activities	Delirium OR obtundation, OR coma
**Ataxia**	Asymptomatic ataxia detectable on exam OR Minimal ataxia causing no or minimal interference with usual social & functional activities	Symptomatic ataxia causing greater than minimal interference with usual social & functional activities	Symptomatic ataxia causing inability to perform usual social & functional activities	Disabling ataxia causing inability to perform basic self-care functions
**CNS ischemia**	N/A	N/A	Transient ischemic attack	Cerebral vascular accident (CVA, stroke) with neurological deficit
**Developmental delay**	Mild developmental delay, either motor or cognitive, as determined by comparison with a developmental screening tool appropriate for the setting	Moderate developmental delay, either motor or cognitive, as determined by comparison with a developmental screening tool appropriate for the setting	Severe developmental delay, either motor or cognitive, as determined by comparison with a developmental screening tool appropriate for the setting	Developmental regression, either motor or cognitive, as determined by comparison with a developmental screening tool appropriate for the setting
**Neuromuscular Weakness**<br><br>(including myopathy & neuropathy)	Asymptomatic with decreased strength on exam OR Minimal muscle weakness causing no or minimal interference with usual social & functional activities	Muscle weakness causing greater than minimal interference with usual social & functional activities	Muscle weakness causing inability to perform usual social & functional activities	Disabling muscle weakness causing inability to perform basic self-care functions OR Respiratory muscle weakness impairing ventilation
**Neurosensory alteration**<br><br>(including paresthesia and	Asymptomatic with sensory alteration on exam or minimal	Sensory alteration or paresthesia causing greater than minimal	Sensory alteration or paresthesia causing inability to perform	Disabling sensory alteration or paresthesia causing inability to

painful neuropathy)	paresthesia causing no or minimal interference with usual social & functional activities	interference with usual social & functional activities	usual social & functional activities	perform basic self-care functions
**GENITOURINARY (GU)**				
**Urinary tract obstruction**<br><br>(e.g., stone)	N/A	Signs or symptoms of urinary tract obstruction without hydronephrosis or renal dysfunction	Signs or symptoms of urinary tract obstruction with hydronephrosis or renal dysfunction	Obstruction causing lifethreatening consequences
**ENDOCRINE/METABOLIC (ENDO)**				
**Abnormal fat accumulation**<br><br>(e.g., back of neck, breasts, abdomen)	Detectable by study participant (or by caregiver for young children)	Detectable on physical exam by health care provider	Disfiguring OR Obvious changes on casual visual inspection	N/A
**Diabetes mellitus**	N/A	New onset without need to initiate medication OR Modification of current medications to regain glucose control	New onset with initiation of medication indicated OR Diabetes uncontrolled despite treatment modification	Life-threatening consequences (e.g., ketoacidosis, hyperosmolar nonketotic coma)
**Hyperthyroidism**	Asymptomatic	Symptomatic causing greater than minimal interference with usual social & functional activities OR Thyroid suppression therapy indicated	Symptoms causing inability to perform usual social & functional activities OR Uncontrolled despite treatment modification	Life-threatening consequences (e.g., thyroid storm)
**Hypothyroidism**	Asymptomatic	Symptomatic causing greater than minimal interference with usual social & functional activities OR Thyroid replacement therapy indicated	Symptoms causing inability to perform usual social & functional activities OR Uncontrolled despite treatment modification	Life-threatening consequences (e.g., myxedema coma)
**Lipoatrophy**<br><br>(e.g., fat loss from the face, extremities, buttocks)	Detectable by study participant (or by caregiver for young children)	Detectable on physical exam by health care provider	Disfiguring OR Obvious on casual visual inspection	N/A

CLINICAL NOT LISTED ON CRF (RECORD AS OTHER)

Symptom	Grade 1<br>MILD	Grade 2<br>MODERATE	Grade 3<br>SEVERE	Grade 4<br>LIFE THREATENING
**Clinical adverse event NOT identified elsewhere in this grading table**	Symptoms causing no or minimal interference with usual social & functional activities	Symptoms causing greater than minimal interference with usual social & functional activities	Symptoms causing inability to perform usual social & functional activities	Symptoms causing inability to perform basic self-care functions OR Medical or operative intervention indicated to prevent permanent impairment, persistent disability, or death
**Acute systemic allergic reaction**	Localized urticaria (wheals) with no medical intervention indicated	Localized urticaria with medical intervention indicated OR Mild angioedema with no medical intervention indicated	Generalized urticaria OR Angioedema with medical intervention indicated OR Symptomatic mild bronchospasm	Acute anaphylaxis OR Life-threatening bronchospasm OR laryngeal edema
**Infection**	Localized, no	Systemic antimicrobial	Systemic antimicrobial	Life-threatening

(any other than HIV infection)	systemic antimicrobial treatment indicated AND Symptoms causing no or minimal interference with usual social & functional activities	treatment indicated OR Symptoms causing greater than minimal interference with usual social & functional activities	treatment indicated AND Symptoms causing inability to perform usual social & functional activities OR Operative intervention (other than simple incision and drainage) indicated	consequences (e.g., septic shock)

DMID Pediatric Toxicity Tables – November 2007

LABORATORY LISTED ON CRF				
**PARAMETER**	**Grade 1 MILD**	**Grade 2 MODERATE**	**Grade 3 SEVERE**	**Grade 4 LIFE THREATENING**
**HEMATOLOGY**				
WBC, decreased	2,000 – 2,500/mm^3	1,500 – 1,999/mm^3	1,000 – 1,499/mm^3	< 1,000/mm^3
Absolute neutrophil count (ANC)	750-1200/mm^3	400-749/mm^3	250-399/mm^3	< 250/mm^3
Hemoglobin (Hb)	8.5 – 10.0 g/dL	7.5 – 8.4 g/dL	6.50 – 7.4 g/dL	< 6.5 g/dL
Platelets, decreased	-	50,000 – 75,000/mm^3	25,000 – 49,999/mm^3	< 25,000/mm^3
ALT (SGPT)	1.1-<2.0 x ULN	2.0-<3.0 x ULN	3.0-8.0 x ULN	> 8.0 x ULN
AST (SGOT)	1.1-<2.0 x ULN	2.0-<3.0 x ULN	3.0-8.0 x ULN	> 8.0 x ULN

LABORATORY NOT LISTED ON CRF				
**PARAMETER**	**Grade 1 MILD**	**Grade 2 MODERATE**	**Grade 3 SEVERE**	**Grade 4 LIFE THREATENING**
Bilirubin (Total)	1.1-<1.5 x ULN	1.5-<2.0 x ULN	2.0-3.0 x ULN	> 3.0 x ULN
Creatinine 3 months-2 years	0.6-0.8 x ULN	0.9-1.1 x ULN	1.2-1.5 x ULN	> 1.5 x ULN
Creatinine 2-12 years	0.7-1.0 x ULN	1.1-1.6 x ULN	1.7-2.0 x ULN	> 2.0 x ULN
Hyperglycemia	116 – 159 mg/dL	160 – 249 mg/dL	250 – 500 mg/dL	> 400 mg/dL
Hypoglycemia	55-65 mg/dL	40-54 mg/dL	30-39 mg/dL	<30 mg/dL or abnormal glucose and mental status changes
Potassium, serum, high	5.0 – 5.9 mEq/L	6.0 – 6.4 mEq/L	6.5 – 7.0 mEq/L	> 7.0 mEq/L
Potassium, serum, low	3.0 – 3.5 mEq/L	2.5 – 2.9 mEq/L	2.0 – 2.4 mEq/L	< 2.0 mEq/L
Sodium, serum, high	-	145 – 149 mEq/L	150 – 155 mEq/L	> 155 mEq/L
Sodium, serum, low	-	130 – 135 mEq/L	124 – 129 mEq/L	< 124 mEq/L
**Urinalysis**				
Hematuria (microscopic)	< 25 RBC/HPF	> 25 RBC/HPF	-	Gross Hematuria
Proteinuria, random collection	1 +	2 +	3 +	4 +

Appendix I. List of drugs used in children and available in Uganda known to cause prolongation of the QT interval

Clarithromycin
Erythromycin
Fluoxetine
Foscarnet
Halofantrine
Haloperidol
Moxifloxacin
Nicardipine
Petamidine
Risperidone
Salmeterol
Sertraline
Thioridazine

Appendix J. Food insecurity and water and sanitation questions for Nutrition Interview (version March 31, 2011)

I'm going to ask you some questions about the circumstances in your household. By "household" we mean those of you that sleep under the same roof and take meals together at least four days a week.

I'm going to begin by asking you 9 questions about food security in your household over the last four weeks (30 days)[1]. I will be asking whether the condition ever happened, or if it rarely (once or twice), sometimes (three to ten times) or often (more than ten times). Remember, these are only questions about the last 4 weeks.

1. Did you worry that your household would not have enough food?	□ 0. Never<br>□ 1. Rarely (1 – 2 times in the last 4 weeks)<br>□ 2. Sometimes (3 – 10 times in the last 4 weeks)<br>□ 3. Often (More than 10 times in the last 4 weeks)<br>□ 8. Don't know
2. Was there any time when you or any of your household members could not eat the food you wanted because you didn't have resources?	□ 0. Never<br>□ 1. Rarely (1 – 2 times in the last 4 weeks)<br>□ 2. Sometimes (3 – 10 times in the last 4 weeks)<br>□ 3. Often (More than 10 times in the last 4 weeks)<br>□ 8. Don't know
3. Was there any time when you or any of your household members could not eat the food you wanted because you didn't have resources?	□ 0. Never<br>□ 1. Rarely (1 – 2 times in the last 4 weeks)<br>□ 2. Sometimes (3 – 10 times in the last 4 weeks)<br>□ 3. Often (More than 10 times in the last 4 weeks)<br>□ 8. Don't know
4. Was there ever a time when you or a household member had to eat a limited variety of foods due to a lack of resources?	□ 0. Never<br>□ 1. Rarely (1 – 2 times in the last 4 weeks)<br>□ 2. Sometimes (3 – 10 times in the last 4 weeks)<br>□ 3. Often (More than 10 times in the last 4 weeks)<br>□ 8. Don't know
5. Did you or a household member have to eat some foods that you didn't want to eat because of lack of resources to obtain other foods?	□ 0. Never<br>□ 1. Rarely (1 – 2 times in the last 4 weeks)<br>□ 2. Sometimes (3 – 10 times in the last 4 weeks)<br>□ 3. Often (More than 10 times in the last 4 weeks)<br>□ 8. Don't know
6. Did you or a household member ever compromise on the portion of food you ate because there was not enough?	□ 0. Never<br>□ 1. Rarely (1 – 2 times in the last 4 weeks)<br>□ 2. Sometimes (3 – 10 times in the last 4 weeks)<br>□ 3. Often (More than 10 times in the last 4 weeks)<br>□ 8. Don't know
7. Was there any point where you or your household member had no food in the household because of lack of resources?	□ 0. Never<br>□ 1. Rarely (1 – 2 times in the last 4 weeks)<br>□ 2. Sometimes (3 – 10 times in the last 4 weeks)<br>□ 3. Often (More than 10 times in the last 4 weeks)<br>□ 8. Don't know

[1] Academy of Education Development, "Food and Nutrition Technical Assistance Project", Version 3, 2007, Available at: http://www.fantaproject.org/downloads/pdfs/HFIAS_v3_Aug07.pdf

8. Did you or a household member sleep hungry at night because of food shortages in the house?	□ 0. Never<br>□ 1. Rarely (1 – 2 times in the last 4 weeks)<br>□ 2. Sometimes (3 – 10 times in the last 4 weeks)<br>□ 3. Often (More than 10 times in the last 4 weeks)<br>□ 8. Don't know
9. Did you or a household member go hungry in day and night because of limited food in the house?	□ 0. Never<br>□ 1. Rarely (1 – 2 times in the last 4 weeks)<br>□ 2. Sometimes (3 – 10 times in the last 4 weeks)<br>□ 3. Often (More than 10 times in the last 4 weeks)<br>□ 8. Don't know

Now I'm going to ask you 9 questions about water and sanitation in your household.[2]

Q1. What is the main source of drinking-water for members of your household?
Piped water into dwelling >>Q4
Piped water to yard/plot >>Q4
Public tap/standpipe >>Q2
Tubewell/borehole >>Q2
Protected dug well >>Q2
Unprotected dug well >>Q2
Protected spring >>Q2
Unprotected spring >>Q2
Rainwater collection >>Q2
Bottled water >>Q1A
Cart with small tank/drum >>Q2
Tanker-truck >>Q2
Surface water (river, dam, lake, pond, stream, canal, irrigation channels)>>Q2
Other (specify)>>Q2

Q1A. What is the main source of water used by your household for other purposes, such as cooking and hand washing?
Piped water into dwelling >>Q4
Piped water to yard/plot >>Q4
Public tap/standpipe
Tubewell/borehole
Protected dug well
Unprotected dug well
Protected spring
Unprotected spring
Rainwater collection
Cart with small tank/drum
Tanker-truck
Surface water (river, dam, lake,pond, stream, canal, irrigation channels)
Other (specify)

Q2. How long does it take to go there, get water, and come back?
No. of minutes >>Q3
Water on premises >>Q4
DK >>Q3

[2] World Health Organization, 2006. "CORE QUESTIONS ON DRINKING-WATER AND SANITATION FOR HOUSEHOLD SURVEYS. Available at http://whqlibdoc.who.int/publications/2006/9789241563260_eng.pdf.

Q3. Who usually goes to this source to fetch the water for your household?
Probe: Is this person under age 15 years? What sex? Circle the code that best describes this person.
Adult woman >>Q4
Adult man >>Q4
Female child (under 15 years) >>Q4
Male child (under 15 years) >>Q4
DK >>Q4

Q4. Do you treat your water in any way to make it safer to drink?
Yes >>Q5
No >>Q6
DK >>Q6

Q5. What do you usually do to the water to make it safer to drink?
Prompt: Anything else? Record all items mentioned
Boil >>Q6
Add bleach/chlorine >>Q6
Strain it through a cloth >>Q6
Use a water filter (ceramic, sand, composite, etc.)>>Q6
Solar disinfection >>Q6
Let it stand and settle >>Q6
Other (specify) >>Q6
DK >>Q6

Q6. What kind of toilet facility do members of your household usually use?
If "flush" or "pour flush" probe: Where does it flush to?
Flush/pour flush to: >>Q7
piped sewer system >>Q7
septic tank >>Q7
pit latrine >>Q7
elsewhere >>Q7
unknown place/not sure/DK where>>Q7
Ventilated improved pit latrine (VIP) >>Q7
Pit latrine with slab >>Q7
Pit latrine without slab/open pit >>Q7
Composting toilet >>Q7
Bucket >>Q7
Hanging toilet/hanging latrine >>Q7
No facilities or bush or field >>Q9
Other (specify) >>Q7

Q7. Do you share this facility with other households?
Yes >>Q8
No >>Q9

Q8. How many households use this toilet facility?
How many other households share this toilet? >>Q9
Can any member of the public use this toilet? >>Q9
DK >>Q9

Q9. The last time your youngest child in the household passed stools, what was done to dispose of the stools?
Child used toilet/latrine
Put/rinsed into toilet or latrine
Put/rinsed into drain or ditch
Thrown into garbage
Buried
Left in the open
Other (specify)
DK

Appendix K. Nutrition questions for focus group discussion or open-ended interviews (Version March 31, 2011)

Introduction

Thank you for taking the time to participate today. I'd like to talk with you to learn more about your nutritional experiences, what you eat, what you don't eat, what you wish you did eat, etc. There are no right or wrong answers. You should consider yourself/ves to be like the teacher, and I'm the student. Please teach me about your food and nutrition! You should know that the things you say today are confidential, they will stay between us only. Do you have any questions for me before we begin?

1. Okay, let's start with questions about the diet of non-pregnant adults

What are your favorite things to eat?

Why do you like them so much?

How frequently do you eat them?

What are the main foods you typically eat?

Are there things that you don't like to eat that you regularly do eat? What are they

Which foods do you spend the most money on every month?

Would you say you grow most of your food, or buy most of it, or somewhere in the middle?

How many meals per day do you eat?

In a typical household, who is responsible for making sure there is food to eat?

Are there things that aren't food that are sometimes eaten , like earth or charcoal? What about other things like that?

2. Questions about foods for pregnant women

What are the best foods for pregnant women to eat? Why?

Do pregnant women here eat them with any regularity? Why or why not?

In your opinion, are there any foods that pregnant women should NOT eat? What are they? What is the reason?

3. Questions about foods for infants and young children

What do you think the ideal length of time for feeding only breast milk to children is? Are there any circumstances when you think breastmilk should not be given? Could prompt with e.g. any illness on the part of the child or mother, after the mother has been traveling, when baby has diarrhea?

What is the best age to continue feeding children any breast milk?

In your opinion, what are the best foods to feed children as soon as they are not drinking only breast milk? Why?

In your village, are these foods regularly given to children? If no, why not?

What are the foods that are regularly given to these very young children?

Which foods do you think should not be fed to very young children, children that are less than one year? Why not?

What do you think are the best foods to give to children once they get a little older, say 1 or 2 years old?

What are the foods that are regularly given to these slightly older children?

When do children start eating the same food as the adults?

Should children be given snacks? What kind? When?

How many meals should t hey eat?

How many meals do they typically eat in your village?

4. Nutritional challenges
(Besides what we discussed above) what are the biggest challenges with food that you face?

Do you see consequences of these problems in your health? Or in your children's health?

How do you try to solve these problems? Could prompt with growing other food, borrowing food from neighbors, feeding smaller meals, skipping meals, etc.

5. Questions about potential supplemental foods
We may have potential supplemental foods on display and for sampling. These could include simsim (sesame) paste, odi (groundnut/peanut paste), mukene (whitebait), fresh milk, millet flour, micronutrient sprinkles (encapsulated micronutrients that can be mixed into porridgey type of foods), micronutrient tablets, and micronutrient syrups.

Which of these foods are good to give children between 6 and 12 months? Why?
Which are good to give children > 12 months? Why?
Which are good for pregnant women? Why?

Explain how each of the micronutrient supplements work.
Can you imagine eating any of these regularly? What would be difficult about it?

Appendix L. Informed Consent to Participate in a Nutrition Interview or Focus Group

Study Title:	A Randomised Controlled Trial of Monthly Dihydroartemisinin-piperaquine versus Monthly Sulfadoxine-pyrimethamine versus Daily Trimethoprim-sulfamethoxazole versus No Therapy for the Prevention of Malaria
Funding Source	NIH/NICHD
UCSF-CHR Number:	H9926-33953
SOM-REC Number:	2009-077
UNCST	HS-580
Site of Research:	Tororo District Hospital<br>Tororo, Uganda
U.S. Principal Investigator:	Grant Dorsey, MD, PhD
Ugandan Principal Investigator:	Moses Kamya, MBChB, MMed, MPH
Date:	October 5, 2011

You are being asked to participate in a study involving a discussion about food and nutrition that is separate from your child's clinic visit today. This discussion may happen in a group of other people or one-on-one. This is a consent form. It will give you information about what is involved in these discussions so you can decide if you would like to participate in these discussions or not. We are asking you to participate in these discussions so that we can better understand how and what you and your child eats and how you feel about certain types of food.

Why is this study being done?

This study is being conducted to learn more about the practices and beliefs concerning food and nutrition in the communities of Tororo district. The things we learn will be used to help find ways we can help people in your community with nutritional needs. This study is sponsored by the National Institute of Child Health and Human Development (NICHD) in the U.S.

How many people will take part in this study?

We will be asking parents/guardians with a child already enrolled in the PROMOTE studies to be involved in these interviews and focus groups.

What will happen if I choose to participate in a one-on-one interview about water and food in my own household?

- You will meet with a staff interviewer who will ask you questions about your household's access to food and water.
- The interview will take no more than 20 minutes of your time.
- All of the interviews and discussions will take place in a private room at the study clinic at Tororo District Hospital.

What will happen if I choose to participate in a discussion about food and nutrition?

- You will meet with an interviewer by yourself or with 1 to 8 other people to discuss food and nutrition.
- We will ask you questions about foods that people like to eat, should eat and actually do eat.
- We will invite you to taste or discuss some foods, like peanut butter, sesame paste, little fish, and micronutrient tablets and syrups.
- The interview will take no more than 1 to 2 hours of your time.
- All of the interviews and discussions will take place in a private room at the study clinic at Tororo District Hospital.

Can I stop being in the study?

Even if you do agree to participate, you may leave the discussion at any time.

What are the benefits of participating?

There will be no direct benefit to you from participating in this study. However, if you choose to participate, your input will help us to design a nutritional intervention to help people who have nutritional needs.

Are there any risks to participating in these discussions?

Some of the discussion questions may make you uncomfortable but you are free to decline to answer any questions you do not wish to answer at any time.

Will my answers to these questions remain private?

With your permission, the discussion today will be recorded. The recorded information will be available to our research team only. Your personal information will remain confidential and your name will never be published or shared with anyone other than the project researchers.

Will I be paid for taking part in this study?

After the discussion is over, we will compensate you for your time.

What are my rights if I take part in this study?

Taking part in this study is your choice. You may choose either to take part or not to take part in the study. If you decide to take part in this study, you may leave the study at any time. No matter what decision you make, there will be no penalty to you in any way. You will not lose any of your regular benefits, and you can still get your care from our institution the way you usually do.

Who can answer my questions about the study?

Dr. Jane Achan and staff are available to explain this study to you and answer your questions. If you have any other questions about the information here, you may call Dr. Achan at (mobile: 0713-410183). You may also contact Prof. James Tumwine (0414-530020) at the Makerere University School of Medicine Research and Ethics Committee which approved this study for questions about participant's rights and research related harm.

Agree to participate in the one-on-one interview only – continue to signature page.
Agree to participate in the focus group only – continue to signature page.
Agree to participate in the one-on-one interview and focus group – continue to signature page
Decline to participate – Reason:____

CONSENT: WHAT YOUR SIGNATURE OR THUMBPRINT MEANS

PARTICIPATION IN RESEARCH IS VOLUNTARY. You have the right to decline to participate or to withdraw at any point in this study without penalty or loss of benefits to which you or your child is otherwise entitled. A copy of this consent form will be given to you. Your signature or thumbprint below means that you have had this study explained to you. Your signature or thumbprint below means you have had the opportunity to ask questions and get answers. If you wish to participate in this study, you should sign or place your thumbprint below.

Name of participant (printed)

Signature of participant | Date

Name of staff administering consent | Position/Title

Signature of staff administering consent | Date

Name of translator (if necessary)

Signature of translator | Date

*If the participant is unable to read and/or write, an impartial witness must be present during the consent discussion. After the written informed consent form is read and explained to the participant, and after he or she has orally consented to his or her participation in this study, and has either signed the consent form or provided his or her fingerprint, the witness must sign and personally date the consent form. By signing the consent form, the witness attests that the information in the consent form and any other written information was accurately explained to, and apparently understood by, the participant, and that consent was freely given.

Name of person witnessing consent (printed)

Signature of person witnessing consent | Date

Appendix M. Chemoprevention Acceptability Questionnaire

Acceptability Questionnaire
1. Do you know why you were giving your child the study drugs?<br>□ Yes<br>□ No (If No, skip next to question # 5)
2. Were the study drugs used to prevent malaria?<br>□ Yes<br>□ No
3. Were the study drugs used to treat malaria?<br>□ Yes<br>□ No
4. Were the study drugs used to prevent or treat illnesses other than malaria?<br>□ Yes<br>□ No
5. Do you think your child benefitted from taking the study drugs?<br>□ Yes<br>□ No
6. Would you recommend the study drugs for other children?<br>□ Yes<br>□ No
7. Would you have liked to have continued to give the study drugs to your child beyond 2 years of age?<br>□ Yes<br>□ No
**For HIV-unexposed children only**<br><br>8. Would you have liked for the study drugs to be started at an early age than 6 months?<br>□ Yes<br>□ No (If No, skip the last question)<br><br>9. At what age would you have like the study drug to be started (chose 1)?<br>□ less than 1 month of age<br>□ 1-3 months of age<br>□ 4-5 months of age
Initials:

Appendix N. Informed Consent to Participate in Chemoprevention Acceptability Questionnaire

Study Title:	A Randomised Controlled Trial of Monthly Dihydroartemisinin-piperaquine versus Monthly Sulfadoxine-pyrimethamine versus Daily Trimethoprim-sulfamethoxazole versus No Therapy for the Prevention of Malaria
Funding Source	NIH/NICHD
UCSF-CHR Number:	H9926-33953
SOM-REC Number:	2009-077
UNCST	HS-580
Site of Research:	Tororo District Hospital<br>Tororo, Uganda
U.S. Principal Investigator:	Grant Dorsey, MD, PhD
Ugandan Principal Investigator:	Moses Kamya, MBChB, MMed, MPH
Date:	October 5, 2011

You are being asked to participate in a questionnaire about the study drug your child has been receiving. This questionnaire will be done one-on-one. This is a consent form. It will give you information about what is involved in these discussions so you can decide if you would like to participate in the questionnaire or not.

Why is this questionnaire being done?

This questionnaire is being conducted to learn more about your opinions about your child taking the study drug. This study is sponsored by the National Institute of Child Health and Human Development (NICHD) in the U.S.

How many people will take part in this study?

We will be asking parents/guardians with a child already enrolled in the PROMOTE Chemoprevention study to be involved in this questionnaire.

What will happen if I choose to participate in the one-on-one questionnaire?

- You will meet with a staff interviewer who will ask you questions about the study drugs your child received.
- The interview will take no more than 10 minutes of your time.
- The discussion will take place in a private room at the study clinic at Tororo District Hospital.

Can I stop being in the study?

Even if you do agree to participate, you may leave the discussion at any time.

What are the benefits of participating?

There will be no direct benefit to you from participating in this questionnaire. However, if you choose to participate, your input will help us to know better about what you know about the study drug and how you feel about your child taking the drug.

Are there any risks to participating in this questionnaire?

Some of the discussion questions may make you uncomfortable but you are free to decline to answer any questions you do not wish to answer at any time. Any answers you provide us will not change your child's care or participation in the study.

Will my answers to these questions remain private?

Your personal information will remain confidential and your name will never be published or shared with anyone other than the project researchers.

Will I be paid for taking part in this questionnaire?

No, participation in this very brief questionnaire is voluntary and will not be paid.

What are my rights if I take part in this questionnaire?

Taking part in this questionnaire is your choice. You may choose either to take part or not to take part in the questionnaire. If you decide to take part in this questionnaire, you may leave the discussion at any time. No matter what decision you make, there will be no penalty to you in any way. You will not lose any of your regular benefits, and you can still get your care from our institution the way you usually do.

Who can answer my questions about the questionnaire?

Dr. Steven Kinara and staff are available to explain this study to you and answer your questions. If you have any other questions about the information here, you may call Dr. Kinara at (mobile: 0772-438856). You may also contact Prof. James Tumwine (0414-530020) at the Makerere University School of Medicine Research and Ethics Committee which approved this study for questions about participant's rights and research related harm.

CONSENT: WHAT YOUR SIGNATURE OR THUMBPRINT MEANS

PARTICIPATION IN RESEARCH IS VOLUNTARY. You have the right to decline to participate or to withdraw at any point in this questionnaire without penalty or loss of benefits to which you or your child is otherwise entitled. A copy of this consent form will be given to you. Your signature or thumbprint below means that you have had this questionnaire explained to you. Your signature or thumbprint below means you have had the opportunity to ask questions and get answers. If you wish to participate in this questionnaire, you should sign or place your thumbprint below.

____

Name of participant (printed)

____

Signature of participant Date

____

Name of staff administering consent Position/Title

____

Signature of staff administering consent Date

____

Name of translator (if necessary)

____

Signature of translator Date

*If the participant is unable to read and/or write, an impartial witness must be present during the consent discussion. After the written informed consent form is read and explained to the participant, and after he or she has orally consented to his or her participation in this study, and has either signed the consent form or provided his or her fingerprint, the witness must sign and personally date the consent form. By signing the consent form, the witness attests that the information in the consent form and any other written information was accurately explained to, and apparently understood by, the participant, and that consent was freely given.

____

Name of person witnessing consent (printed)

____

Signature of person witnessing consent Date

Appendix O. Household Questionnaire

Household	Question	Responses
**Section 1: Identification**		
1	Date of interview	DD-MM-YYYY
2	Do you agree to participate in this questionnaire?	Yes<br>No
3	Participant ID Number	3001-3607
**Section 2: Household Characteristics (to be asked of the study participant's primary care giver)**		
4	Has the study participant lived in this same home since enrollment in the study?	1 - Yes<br>2 - No<br>8 - Don't Know<br>9 - No Response
5	If No: When did they move here?	DD-MM-YYYY
6	New GPS Coordinates of home	
7	What is the main source of drinking water for members of your household?	10 - borehole<br>11 - pipe into dwelling<br>12 - piped into yard/compound<br>13 - public tap<br>21 - open well in yard/compound<br>22 - open public well<br>31 - protected well in yard/compound<br>32 - protected public well<br>41 - protected spring<br>42 - unprotected spring<br>43 - river/stream<br>44 - pond/lake<br>45 - dam<br>51 - rainwater<br>61 - water truck<br>71 - bottled water<br>96 - other<br>99 - Refused to answer/skipped
8	Specify other source of water	
9	Where do you usually do your cooking?	1 - In home in room where study participant sleeps<br>2 - In home in different room from where study participant sleeps<br>3 - In separate detached and enclosed space<br>4 - In the open air / outside<br>8 - Don't Know

		9 - Refused to Answer
10	What type of fuel does your household mainly use for cooking?	1 - electricity<br>2 - lpg/natural gas<br>3 - biogas<br>4 - paraffin/kerosene<br>5 - charcoal<br>6 - firewood<br>7 - starw/shrubs/grass<br>8 - animal dung<br>95 - no food cooked in household<br>96 - other<br>99 - Refused to answer
11	Specify other type of fuel used	
12	What kind of toilet facility do members of your household usually use?	1 - flush toilet<br>2 - vip latrine<br>3 - covered pit latrine no slab<br>4 - covered pit latrine w/slab<br>5 - uncovered pit latrine no slab<br>6 - uncovered pit latrine w/slab<br>7 - composting toilet<br>8 - no facility/bush/field<br>96 - other<br>99 - Refused to answer
13	Specify other kind of toilet facilities	
14	What is the main source of energy for lighting in the household?	1 - electricity<br>2 - solar<br>3 - gas<br>4 - paraffin - hurricane lamp<br>5 - paraffin - pressure lamp<br>6 - paraffin - wick lamp<br>7 - firewood<br>8 - candles<br>96 - other<br>99 - Refused to answer/skipped
15	Specify other source of energy for lighting	

16	MAIN MATERIAL OF THE FLOOR<br><br>RECORD OBSERVATION.<br><br>MARK ONLY ONE.	11 - earth/sand<br>12 - earth and dung<br>31 - parquet or polished wood<br>33 - mosaic or tiles<br>34 - bricks<br>35 - cement<br>36 - stones<br>96 - other<br>99 - Refused to Answer
17	Specify other material of the floor	
18	MAIN MATERIAL OF THE ROOF.<br><br>RECORD OBSERVATION.<br><br>MARK ONLY ONE.	11 - thatched<br>12 - mud<br>13 - sticks<br>21 - wood/planks<br>22 - iron sheets<br>24 - tiles<br>25 - cement<br>28 - plastic/tarp<br>96 - other<br>99 - Refused to Answer
19	Specify other material of the roof	
20	MAIN MATERIAL OF THE EXTERIOR WALLS.<br><br>RECORD OBSERVATION.<br><br>MARK ONLY ONE.	11 - thatched/straw<br>21 - mud and poles<br>22 - un-burnt bricks<br>23 - un-burnt bricks with plaster<br>24 - burnt bricks with mud<br>31 - cement blocks<br>32 - stone<br>33 - timber<br>34 - burnt bricks with cement<br>96 - other<br>99 - Refused to Answer
21	Specify other material of the exterior walls	
22	Is there livestock in the home (cattle sheep chickens)?	1 - Never<br>2 - Seldom<br>3 - Sometimes<br>4 - Often<br>5 - Always<br>8 - Don't Know<br>9 - Refused to Answer

23	How many rooms are in your house?<br><br>(INCLUDING ROOMS OUTSIDE THE MAIN DWELLING)<br><br>If there are 15 or more rooms, enter 15	Range 1--15<br>98 - Don't Know<br>99 - Refused to Answer
24	How many usual household members live here?	1 -- 20<br>Don't Know<br>Refused to Answer
25	How many of those usual household members are under the age of 5?	Range 1 -- 20<br>98 - Don't Know<br>99 - Refused to Answer
26	How many rooms in your household are used for sleeping?<br><br>(INCLUDING ROOMS OUTSIDE THE MAIN DWELLING)<br><br>If there are 15 or more rooms, enter 15	Range 1-15<br>98 - Don't know<br>99 - Refused to answer/skipped
27	Does your household have any mosquito nets that can be used while sleeping?	1 - Yes<br>2 - No<br>8 - Don't know<br>9 - Refused to answer
28	How many mosquito nets does your household have that are used?<br><br>IF MORE THAN 12, ENTER 13	Continuous number<br>99 - Refused to answer
29	Of those mosquito nets that you use, how many are treated with insecticide?	Range 1--15<br>98 - Don't Know<br>99 - Refused to Answer
30	Has the inside of your house been sprayed with insecticide in the last 12 months?	1 - Yes<br>2 - No<br>8 - Don't Know<br>9 - Refused to Answer
31	If yes, when was it sprayed?	Date DD/MM/YYYY
**Section 3: Study Participants Sleeping Area Characteristics**		
32	OBSERVATION: Note shape of room where study participant sleeps	1 - Circular<br>2 - Rectangular
33	If circular: measure diameter	
34	If rectangular: measure length	
35	If rectangular: measure width	

36	OBSERVATION: How many entryways into the room are there?	Range 1-5
	For each entryway, repeat the following questions:	
39	OBSERVATION: Does it open to the outside?	1 - Yes<br>2 - No
37	OBSERVATION: Is the entry way covered?	1 - Covered<br>2 - Partially Covereed<br>3 - Open
38	OBSERVATION: Main material is the covering made of.	1 - Wood<br>2 - Screen<br>3 - Metal<br>4 - Cloth<br>5 - Other
39	Specify Other covering type	
40	OBSERVATION: How many windows are in the room?	Range 0-10
	For each window, repeat the following:	
41	OBSERVATION: Is the window covered?	1 - Completely Covered<br>2 - Partially Covered<br>3 - Open
42	OBSERVATION: Does the window open to the outside	1 - Yes<br>2 - No
43	OBSERVATION: Does the room have eaves?	1 - Yes<br>2 - No
44	OBSERVATION: If room has eaves, are the eaves covered?	1 - Completely Covered<br>2 - Partially Covered<br>3 - Open
45	OBSERVATION: Do the eaves open to the outside?	1 - Yes<br>2 - Partially<br>3 - No
46	Usually, how many people sleep in the same room as the study participant?	Range 1--20<br>98 - Don't know<br>99 - Refused to answer
47	How many of those people are under 5 years old?	Range 1--10<br>98 - Don't know<br>99 - Refused to answer
48	How many sleeping areas are in the room where the study participant sleeps?	Range 1--10<br>98 - Don't know<br>99 - Refused to answer
49	For these sleeping areas, how many do you usually use a bed net for?	Range 1--10<br>98 - Don't know<br>99 - Refused to answer
	Ask for each sleeping area with a bed net, ask the following question:	
50	Is the bed net treated with insecticide?	1 - Yes<br>2 - No<br>3 - Don't Know<br>4 - No Response

51	Where does the child usually sleep?	1 - Directly on the ground<br>2 - On a mattress/mat on the ground<br>3 - On a bed/platform raised off the ground<br>4 - Hanging hammock<br>5 - Other<br>8 - Don't Know<br>9 - No Response
52	Specify other sleeping area	
53	How many people sleep in the same bed/sleeping area as the child under the mosquito net?	Range 0-10<br>98 - Don't know<br>99 - No Response
54	How many of those people are under 5 years old?	Range 0-10<br>98 - Don't know<br>99 - No Response
55	Are the windows in this room usually open when the study participant sleeps?	1 - Yes<br>2 - No<br>3 - Don't Know<br>4 - No Response
56	Are there any holes in the net that the study participant sleeps under?	1 - Yes<br>2 - No<br>3 - Don't Know<br>4 - No Response

Primary Caregiver	Question	Responses
**Section 1: Identification**		
1	Date of interview	DD-MM-YYYY
2	Do you agree to participate in this questionnaire?	Yes<br>No
3	Participant ID Number	3001-3607
**Section 2: Respondent characteristics**		
4	OBSERVATION: Is respondent male or female	1 - male<br>2 - female
5	What is your relationship to the study participant?	1 - Parent<br>2 - Sibling<br>3 - Grandparent<br>4 - Aunt/Uncle<br>5 - Other family member<br>6 - Non-family member
6	How old are you?	999 - Refuse to answer
7	What is your current marital status?	1 - Married<br>2 - Widowed<br>3 - Divorced<br>4 - Separated<br>5 - Never married

8	Have you ever attended school?	1 - Yes<br>2 - No<br>9 - Refused to answer
9	What is the highest level of school you attended:<br>primary, '0' level, 'A' level, or university or tertiary?	1 - Primary<br>2 - 'O' level<br>3 - 'A' level<br>4 - University/tertiary<br>9 - Refused to answer
10	As you know, some women take up jobs for which they are paid in cash or kind. Others sell things, have a small business or work on the family farm or in the family business. Do you usually do work of this type or any other work?	1 - Yes<br>2 - No<br>9 - Refused to answer
11	Are usually you paid in cash or kind for this work or are you usually not paid at all?<br><br>IF PAID, ASK WHETHER CASH, IN-KIND, OR BOTH.	1 - Cash only<br>2 - Cash and kind<br>3 - In-kind only<br>4 - Not paid<br>9 - Refused to answer
12	What is your occupation, that is, what kind of work do you mainly do?<br><br>INTERVIEWER: PROBE TO OBTAIN DETAILED INFORMATION ON THE KIND OF WORK RESPONDENT DOES.	
13	What have you been doing for most of the time over the last 12 months?	1 - going to school/studying<br>2 - looking for work<br>3 - retired<br>4 - too ill to work<br>5 - handicapped, cannot work<br>6 - housework/child care<br>96 - other<br>99 - Refused to answer
14	Specify other non-work activity	
**Section 3: Care-taking of the Study Participant**		
15	Now I would like to ask you about the food your child (study participant) eats.<br><br>How many meals does the child normally have per day?	9 - Refused to answer/skipped

16	How often do you have problems in meeting the food needs for the child?	1 - never<br>2 - seldom<br>3 - sometimes<br>4 - often<br>5 - always<br>8 - Don't know<br>9 - Refused to answer/skipped
17	Using your best estimate, what time in the morning does the child leave the bed when s/he wakes up?	1 - Before 6AM<br>2 - Between 6AM and 8AM<br>3 - Between 8AM and 10AM<br>4 - After 10AM<br>8 - Don't Know<br>9 - No Response
18	Using your best estimate, when does the child leave the house in the morning?	1 - Before 6AM<br>2 - Between 6AM and 8AM<br>3 - Between 8AM and 10AM<br>4 - After 10AM<br>8 - Don't Know<br>9 - No Response
19	Using your best estimate, what time does the child return to the house in the evening?	1 - Before 6PM<br>2 - Between 6PM and 8PM<br>3 - Between 8PM and 10PM<br>4 - After 10PM<br>8 - Don't Know<br>9 - No Response
20	Using your best estimate, when does the child get under the mosquito net in the evening?	1 - Before 6PM<br>2 - Between 6PM and 8PM<br>3 - Between 8PM and 10PM<br>4 - After 10PM<br>8 - Don't Know<br>9 - No Response
**Section 4: Home improvement**		
21	Now I'd like to ask you some questions about your home. If money were not an issue, what type of improvement to your home would you be MOST interested in?	1 - Floor<br>2 - Roof<br>3 - Eaves<br>4 - Window(s)<br>5 - Door(s)<br>6 - Walls<br>7 - Toilet<br>8 - Kitchen<br>9 - Other<br>98 - Don't know<br>99 - Refused to answer
22	What is the main reason for making this change?	1 - It is broken<br>2 - It's not broken, but it is old<br>3 - It is too dirty / doesn't look good<br>4 - Keep animals/insects out<br>5 - Other<br>8 - Don't Know

		9 - No response
23	Specify other reason for desired change	
24	What is the main benefit you would get from this change?	1 - The house will be safer<br>2 - The house will be more comfortable to live in<br>3 - The house will look nicer<br>4 - The house will be cleaner<br>5 - Other<br>8 - Don't Know<br>9 - No Response
25	Specify other reason for benefit to home	

Appendix P. Household Questionnaire Verbal Consent script

My name is ___________________________, a research assistant. I work with the PROMOTE program with the MU-UCSF Research Collaboration based in Tororo District Hospital. We are conducting a questionnaire of all the households with a child enrolled in the PROMOTE-Chemoprevention study so that we may learn basic information about your household including questions such as the number of total rooms in the house, number of total bed nets in your house, the water source and food supply for the house. This information will help us to learn more about malaria transmission in your community.

We would like to ask these questions to the Primary Caregiver of the child who is in PROMOTE-Chemoprevention study. The questions will take 1 – 2 hours but will require some observation of your household. If the Primary Caregiver is not available at this time or needs more time to consider this questionnaire participation, we can return to your household.

The information will be collected on a computer that is password protected and will be locked in a secure office. The results of this questionnaire may be published in a medical journal or be made available to other health professionals, but we will not include your or any other household member's name or any other information that could identify you.

Your household's participation in this questionnaire is voluntary. You have the right to decline your household's participation in the questionnaire today, or withdraw your household's participation at any time. For any questions or concerns about the census activities, please call Dr. Victor Bigira at 0711787126.

At this time, do you want to ask me anything about the questionnaire? Would the Primary Caregiver like to participate in the questionnaire at this time or can we schedule a time to return to your household?

PRIMARY CAREGIVER AGREES TO PARTICIPATE: ☐ Yes ☐ No

Signature of Research Assistant: ____________________ Date: ____________
