# Supplementary material for: Protective Efficacy and Safety of Three Antimalarial Regimens for the Prevention of Malaria in Young Ugandan Children: A Randomized Controlled Trial
Source: PLoS Med. 2014 Aug 5;11(8):e1001689. doi: 10.1371/journal.pmed.1001689 (PMC4122345; doi:10.1371/journal.pmed.1001689)
Supplement: Text S3 — Institutional review board approval from the University of California, San Francisco. (PDF) [file pmed.1001689.s005.pdf]

University of California
San Francisco

Human Research Protection Program
Committee on Human Research

Notification of Full Committee Approval

Principal Investigator
Matthew G Dorsey

Co-Principal Investigator
Philip J Rosenthal

Type of Submission: Continuing Review Submission Form
Study Title: "PROMOTE – CHEMOPREVENTION": A randomized controlled trial of monthly dihydroartemisinin-piperaquine versus monthly sulfadoxine-pyrimethamine versus daily trimethoprim-sulfamethoxazole versus no therapy for the prevention of malaria

IRB #: 10-01489
Reference #: 072593

Reviewing Committee: San Francisco General Hospital Panel

Study Risk Assignment: Greater than minimal

Approval Date: 09/05/2013 **Expiration Date:** 09/07/2014

Regulatory Determinations Pertaining to This Approval:

This research satisfies the following condition(s) for the involvement of children:
45 CFR 46.405, 21 CFR 50.52: Research involving greater than minimal risk but presenting the prospect of direct benefit to the individual subjects.

Parental Permission and Assent:
The permission of one parent or guardian is sufficient.

This research is not subject to HIPAA rules.

All changes to a study must receive CHR approval before they are implemented. Follow the modification request instructions. The only exception to the requirement for prior CHR review and approval is when the changes are necessary to eliminate apparent immediate hazards to the subject (45 CFR 46.103.b.4, 21 CFR 56.108.a). In such cases, report the actions taken by following these instructions.

Expiration Notice: The iMedRIS system will generate an email notification eight weeks prior to the expiration of this study's approval. However, it is your responsibility to ensure that an application for continuing review approval has been submitted by the required time. In addition, you are required to submit a study closeout report at the completion of the project.

Approved Documents: To obtain a list of documents that were approved with this submission, follow these steps: Go to My Studies and open the study – Click on Submissions History – Go to Completed Submissions – Locate this submission and click on the Details button to view a list of submitted documents and their outcomes.

For a list of all currently approved documents, follow these steps: Go to My Studies and open the study – Click on Informed Consent to obtain a list of approved consent documents and Other Study Documents for a list of other approved documents.

San Francisco Veterans Affairs Medical Center (SFVAMC): If the SFVAMC is engaged in this research, you must secure approval of the VA Research & Development Committee in addition to CHR approval and follow all applicable VA and other federal requirements. The CHR website has more information.
