# Supplementary material for: Protective Efficacy and Safety of Three Antimalarial Regimens for the Prevention of Malaria in Young Ugandan Children: A Randomized Controlled Trial
Source: PLoS Med. 2014 Aug 5;11(8):e1001689. doi: 10.1371/journal.pmed.1001689 (PMC4122345; doi:10.1371/journal.pmed.1001689)
Supplement: Text S1 — Institutional review board approval from Makerere University School of Medicine Research and Ethics Committee. (PDF) [file pmed.1001689.s003.pdf]

MAKERERE UNIVERSITY

P.O. Box 7072 Kampala, Uganda
E-mail: rresearch9@gmail.com

Phone: 256 414 533541
Fax: 256 414 541036/0414 532204

COLLEGE OF HEALTH SCIENCES
SCHOOL OF MEDICINE

RESEARCH ETHICS COMMITTEE

February 14, 2013

Prof. Moses Kamya
Department of Internal Medicine

Category of review

[ ] Initial review
[X] Continuing review
[ ] Amendment
[ ] Termination of study
[ ] SAEs

Dear Prof. Kamya,

RE: REC REF 2009-077
Title: "A randomized controlled trial of monthly dihydroaartemisinin-piperaquine versus monthly sulfadoxine-pyrimethamine versus daily trimethoprim-sulfamethoxazole versus no therapy for the prevention of malaria"

Renewal Approval Date: 14th February, 2013
Project Expiration Date: 29th April, 2014

On behalf of the committee, I write to inform you that the **proposed extension has been approved.**

The School of Medicine Research and Ethics Committee (REC) initially reviewed and approved the above-referenced protocol on April 30, 2009. Previous approval of this protocol expires on 12th March, 2013.

The review by the committee has found that your renewal is consistent with the continued protection of the rights and welfare of human subjects which protection of human subjects is a partnership between the Research and Ethics Committee (REC) and the investigators. We look forward to working with you as we both fulfill our responsibilities.

Renewals: REC approval is valid until the expiration date given above. If you are continuing your project, you must submit an Application for renewal at least six (6) to eight (8) weeks before the

lapse date. If the project is completed, please submit an application for permanent closure.

Amendments: The REC must review any changes in the project, prior to initiation of the change. Please submit an Application for Amendments to have your changes reviewed and summarize the proposed change and the rationale for it in a letter to the School of Medicine Research and Ethics Committee. If changes are made at the time of renewal, please include an Application for Amendments with the renewal application.
In addition, submit three (3) copies of an updated version of your original protocol application-one showing all proposed changes in bold or 'track changes,' and the other without bold or track changes.

Adverse Events: If issues should arise during the conduct of the research, such as unanticipated problems, severe adverse events or any other problem that may increase the risk to the human subjects, notify the REC Chairman promptly. The forms are available to report these issues.

Please use the REC REF number listed above on any forms submitted which relate to this project/study.

Good luck in your research. If we can be of further assistance, please contact us at (+256) 0414 - 533541 or via email at research@chs.mak.ac.ug/rresearch9@gmail.com. Thank you for your cooperation.

Yours Sincerely,

MAKERERE UNIVERSITY
SCHOOL OF MEDICINE
APPROVED
VALID UNTIL
29 APR 2014
RESEARCH & ETHICS COMMITTEE
P.O.BOX 7072, KAMPALA

Prof. James Tumwine
Chairperson School of Medicine Research and Ethics Committee
