# Supplementary material for: Protective Efficacy and Safety of Three Antimalarial Regimens for the Prevention of Malaria in Young Ugandan Children: A Randomized Controlled Trial
Source: PLoS Med. 2014 Aug 5;11(8):e1001689. doi: 10.1371/journal.pmed.1001689 (PMC4122345; doi:10.1371/journal.pmed.1001689)
Supplement: Text S2 — Institutional review board approval from the Uganda National Council for Science and Technology. (PDF) [file pmed.1001689.s004.pdf]

Uganda National Council for Science and Technology

(Established by Act of Parliament of the Republic of Uganda)

UNCST

Our Ref: HS 580 **5th June 2013**

Prof Moses Kamya
MU-UCSF Malaria Research Collaboration
P O Box 7475
Kampala

Dear Prof. Kamya,

RE: RESEARCH PROJECT, "A RANDOMISED CONTROLLED TRIAL OF MONTHLY DIHYDROARTEMISININ-PIPERAQUINE VERSUS MONTHLY SULFADOXINE-PYRIMETHAMINE VERSUS DAILY TRIMETHOPRIM-SULFAMETHOXAZOLE VERSUS NO THERAPY FOR THE PREVENTION OF MALARIA"

This is to inform you that on 4th June 2013, Uganda National Council for Science and Technology (UNCST) reviewed and approved your request to continue with the above study.

The study is valid until 12th June 2014. If however, it is necessary to continue with the study beyond the expiry date, a request for continuation should be made to the Executive Secretary, UNCST

Yours sincerely,

Jane Nabbuto
for: Executive Secretary
UGANDA NATIONAL COUNCIL FOR SCIENCE AND TECHNOLOGY

LOCATION/CORRESPONDENCE

Plot 6 Kimera Road, Ntinda
P.O. Box 6884

COMMUNICATION

TEL: (256) 414 705500, (256) 312 314800
FAX: (256) 414-234579
